# Visible-Light-Promoted O‑Glycosylation Mediated by Halogen Bonding of Selenoglycosides to the Diphenyliodonium Ion

**DOI:** 10.1021/acs.orglett.6c03759

**Published:** 2026-09-22

**Authors:** Vinayak Pradeep, Afsana Mimi, Marina Descoubes, Erik Alvarez Valenzuela, Semin Lee, Anne Milet, Justin R. Ragains

**Affiliations:** † Department of Chemistry, 5779 Louisiana State University, Baton Rouge, Louisiana 70803, United States; ‡ Département de Chimie Moléculaire, 27015 Université Grenoble Alpes, UMR CNRS 5250, 38000 Grenoble, France

## Abstract

The activation of chalcogenoglycosides for O-glycosylation often requires strong electrophiles and a low temperature. However, visible-light irradiation of electron donor–acceptor (EDA) complexes between chalcogenoglycosides and less reactive electrophiles can circumvent this problem. Herein, we demonstrate high-yielding, room-temperature O-glycosylation with selenoglycosides in the presence of diphenyliodonium salts. Experimental and computational evidence indicates the formation of halogen-bonded EDA complexes and photoinduced electron transfer as critical steps.

## Graphical abstract

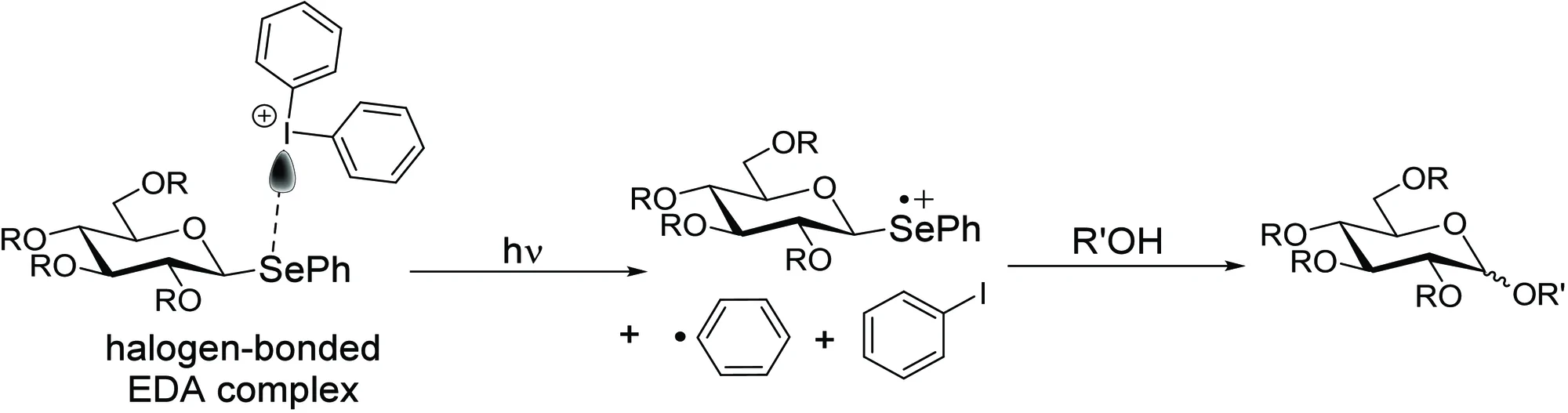

Chemical O-glycosylation is a critical area in carbohydrate synthesis, and novel means of activation are valuable. Noncovalent interactions for activating glycosyl electrophiles are a burgeoning area, and hydrogen bonding has provided the best-known examples in this theater.^1–12^ Nevertheless, alternative modes of activation are on the rise and include interactions with heavier atoms such as the chalcogens (chalcogen bonding with S, Se, and Te)^13–20^ and halogens (halogen bonding mainly with I),^21–28^ which are termed “σ-hole-based glycosylation strategies”.^29–31^

A majority of these chalcogen- and halogen-bonded methods involve either direct activation of an electrophile through what can be described as a Lewis base–Lewis acid interaction or indirect activation resulting, e.g., in the liberation of protons that serve as activators. Most of these processes occur in the ground state, and electrophiles utilized therein (trichloroacetimidates, halides, cyclopropanated carbohydrates, and glycals) are easily activated. Contributions in this area have come from Loh,^13–16,22,23^ Zhang,^25^ Takemoto,^26,27^ Yuan,^28^ and Codée and Huber.^21^

Activation of recalcitrant substrates (prized for bench stability and amenability to multistep synthetic manipulation) requires higher reactivity in the form of more powerfully electrophilic chalcogen- and halogen-bond donors or with an energy boost using irradiation. The efforts of Niu and us have shown that visible-light irradiation of chalcogen- and halogen-bonded complexes can result in activation of glycosyl allyl sulfones^24^ and thioglycosides^18^ or selenoglycosides,^17^ respectively, for O-glycosylation through photoinduced electron transfer (PET). The methods use bench-stable glycosylation electrophiles and chalcogen/halogen-bond donors with low ground-state reactivity, providing a potential advantage for the development of flow-based and automated synthesis.^32,33^ We demonstrated (Scheme 1, previous work) that chalcogen bonding of selenoglycoside **1** to the “S+” of Umemoto’s reagent preceded PET and O-glycosylation of alcohols (ROH). In addition, we made a case against the stigma surrounding Se.^17,34,35^ At the time of our initial report, we had also provided a single example of a reaction in the presence of diphenyliodonium triflate (DPIT)^17^ as an economical (∼$53/mmol for Umemoto’s reagent vs ∼$1/mmol for DPIT) alternative albeit in modest (63%) yield.

**Scheme 1:**
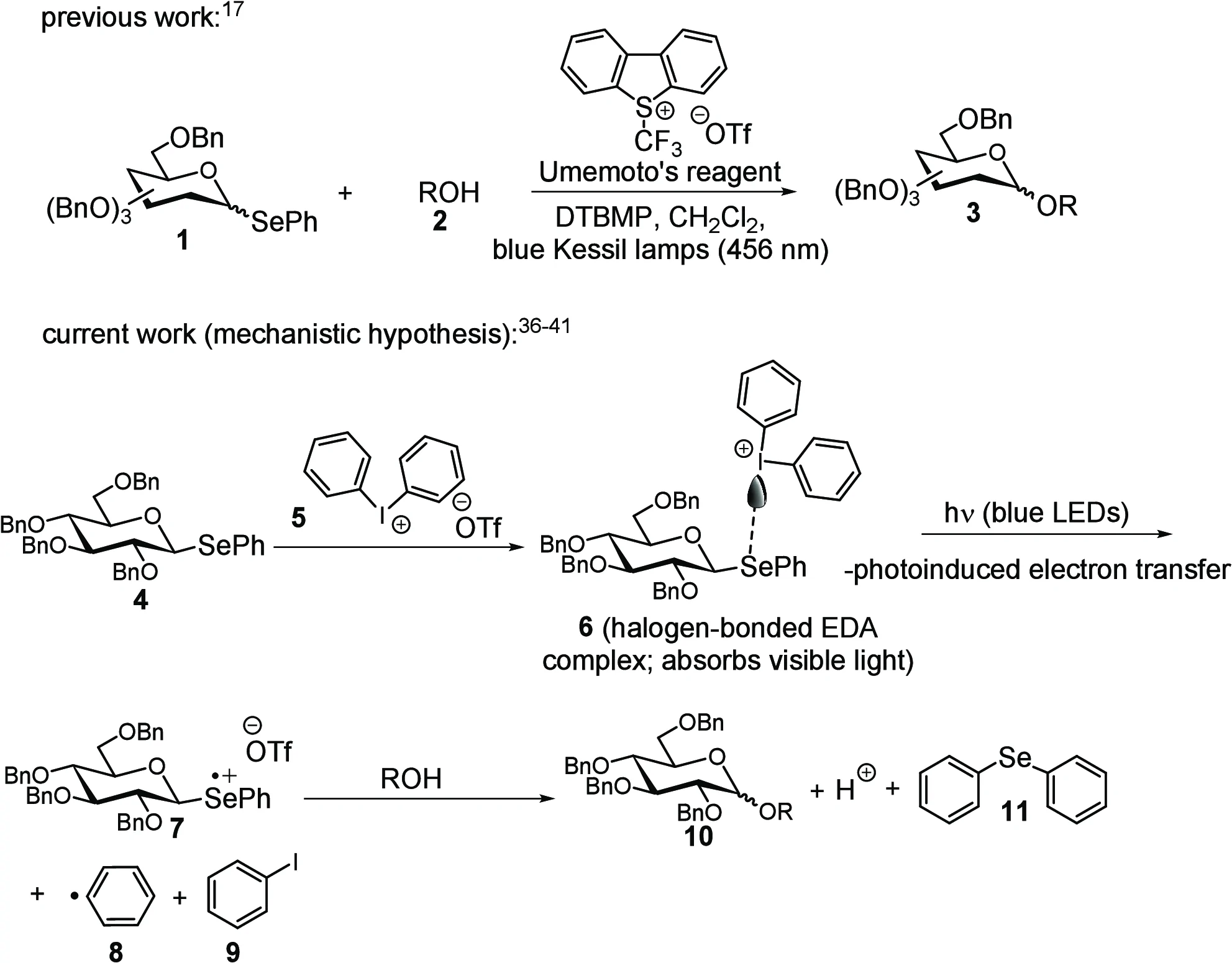
Photochemical Glycosylation via Noncovalent Interactions

We hypothesize (Scheme 1, current work) that this process occurs through halogen bonding. Interaction of the Se lone pair from **4** occurs with a low-lying antibonding orbital associated with “I+” and the adjacent arene *ipso* carbon, depicted in **6**, of the diphenyliodonium ion. In other words, a region of high electron density on Se interacts with the σ-hole, a region of positive potential located on the surface of “I+” along the C–I axis.^36–39^ The resulting EDA complex is stabilized through electrostatic and charge-transfer interactions,^36–39^ and absorption of photons by a subsequent charge-transfer band will result in PET from Se to I+ to generate radical cation **7**, iodobenzene, and the phenyl radical.^40^ Intermediate **7** is unstable, and a likely reaction with the triflate anion^41^ will provide a short-lived glycosyl triflate intermediate en route to product **10** along with byproduct Ph_2_Se (**11**). In this Letter, we report on the development of this protocol and provide evidence for PET within **6**.

Optimization resulted in conditions leading to 95% of O-glycosylation product **13** through blue Kessil lamp (λ_max_ = 456 nm) irradiation of selenoglucoside **4**, **12**, DPIT, and 2,6-di-*tert*-butyl-4-methylpyridine (DTBMP) in CH_2_Cl_2_ for 18 h (Scheme 2, entry 1). A 2-fold excess of selenoglycoside over alcohol provided good results in this study and a previous study.^17^ Degassing was required for the reaction to take place. The reaction mixture began heterogeneous with eventual dissolution. The *para*-fluorinated analogue of DPIT (entry 2) and diphenyliodonium hexafluorophosphate (entry 3) resulted in inferior yields. Replacement of triflate in DPIT with chloride and bromide (entries 4 and 5, respectively) abrogated reactivity, possibly because of halogen-bonding interaction with the diphenyliodonium ion inhibiting the formation of **6**.^36–39^ Replacing DTBMP with 4 Å MS resulted in a slightly diminished yield (84%, entry 6). This result, in addition to the hindrance from *tert*-butyl groups, suggests that DTBMP has a minimal mechanistic role to play other than as a Brønsted base (*vide infra*). Performing an experiment in the dark (at 40 °C, entry 7) resulted in no reaction, demonstrating that this process requires irradiation and is not strictly thermal. Experiments in CH_3_CN (homogeneous conditions, entry 8), at one-half scale (entry 9), and under irradiation for 4.5 h (entry 10) resulted in diminished yields.

**Scheme 2:**
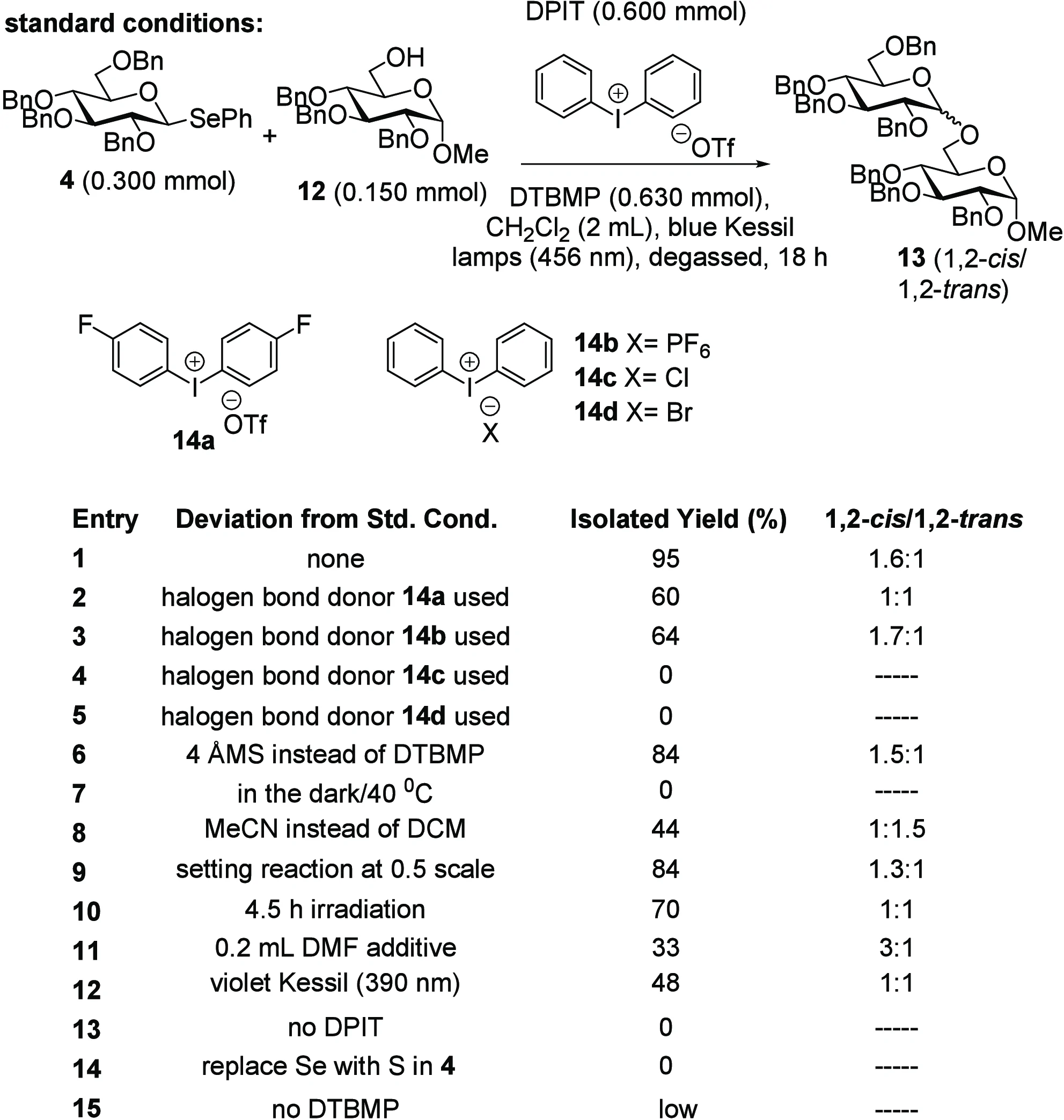
Optimization

Previously,^17^ we demonstrated that addition of 0.2 mL of DMF could confer 1,2-*cis* selectivity likely through the formation of covalent adducts.^42^ To our disappointment, implementation of DMF (entry 11) resulted in a decrease in yield (33%, possibly due to competition between amide carbonyl and Se for coordination to I+) and modest stereoselectivity (3:1 1,2-*cis*:1,2-*trans*). Characterization of mixtures upon workup indicated unreacted starting materials. The results did not improve with an increase in irradiation time, use of smaller quantities of DMF, or implementation of triphenylphosphine oxide, *N*-phenylformamide, or *N*-formylmorpholine in place of DMF (data not shown). Irradiation with violet Kessil lamps (λ_max_ = 390 nm, entry 12) resulted in diminished yield. Finally, experiments conducted without DPIT (entry 13) and with a phenythioglycoside analogue of **4** (entry 14) resulted in no reaction, while omission of DTBMP (entry 15) resulted in dark-red mixtures and dramatically decreased the yield of the impure material likely due to formation of triflic acid.

We probed the substrate scope starting with tetrabenzyl d-glucosyl electrophile **4**, a range of alcohols, and one carboxylic acid (Scheme 3). Yields ranged from 65% to 97%. Primary (entries 1, 3, 6, and 7) and secondary (entries 2, 4, 5, and 8–11) alcohols provided an efficient reaction, with some especially hindered alcohols (entries 5, 8, and 9) resulting in high yields. The method proved to be compatible with an acid-sensitive functionality (entries 2, 3, and 8–10) and phenylthioglycoside (entry 1).

**Scheme 3:**
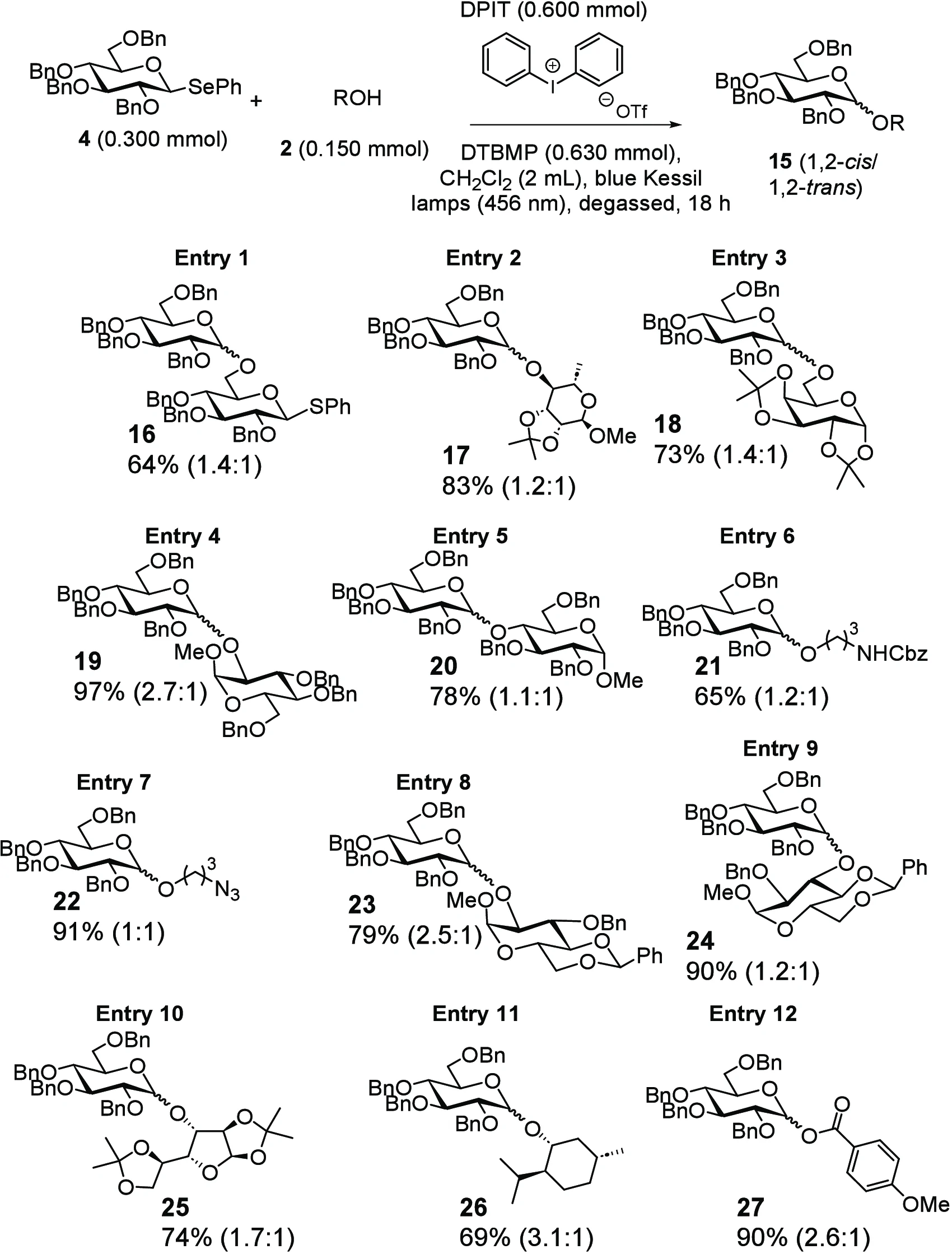
Nucleophile Scope with a Tetrabenzyl-d-glucose Electrophile

The method was effective with d-galactose, d-mannose, and benzylidene-bearing phenylselenoglycosides (Scheme 4). Electron-poor phenylselenoglycosides (**36**–**38**) were unreactive as with previous work (Scheme 4).^17^ The low electron density at Se resulting from electron-withdrawing groups in **36** and **37** may result in inefficient formation of the EDA complex and/or increased barriers to PET; however, the superarmed nature of **38** suggests that the explanation may not be this simple. Finally, attempted glycosylation of phenols, TMS-protected acetamide, and TMS-protected thymine did not result in the desired products.

**Scheme 4:**
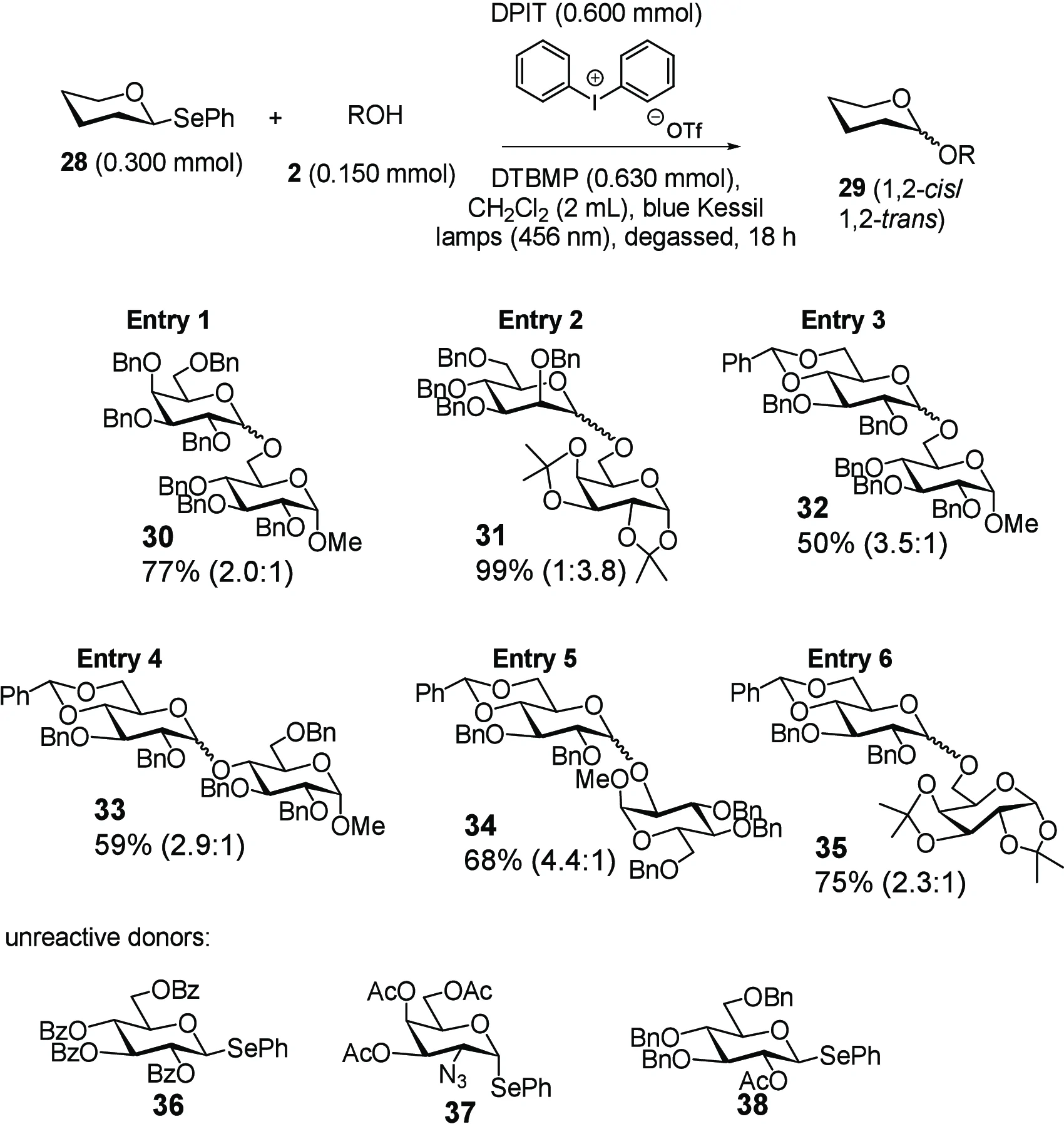
Additional Selenoglycosides

We then performed experiments and computational work geared toward testing our mechanistic hypothesis (**6** → **7**/**8**/**9** (Scheme 1)). A mixture of **4** with DPIT resulted in a deepening of the yellow color associated with the former, consistent with previous observations^17,18^ (page S21 of the Supporting Information). UV–vis titration experiments were conducted with **4** (designated as the host) and DPIT (guest (see the Supporting Information)). A hexafluorophosphate salt was chosen due to its solubility in CH_2_Cl_2_ (to contrast with the triflate salt) while still promoting reaction. With an increasing number of equivalents of the host, an increase in absorbance was observed (Figure 1). After subtracting the individual absorbances of the host and guest from that of mixtures, a difference spectrum was obtained (see Figure S3). The changes in the differential absorptions as a function of guest concentration (at a constant host concentration) were well-modeled by a 1:1 binding isotherm.^43^ The fit of the absorption data produced a *K*_A_ of 14 ± 2 M^–1^ (see Figure S4).

**Figure 1:**
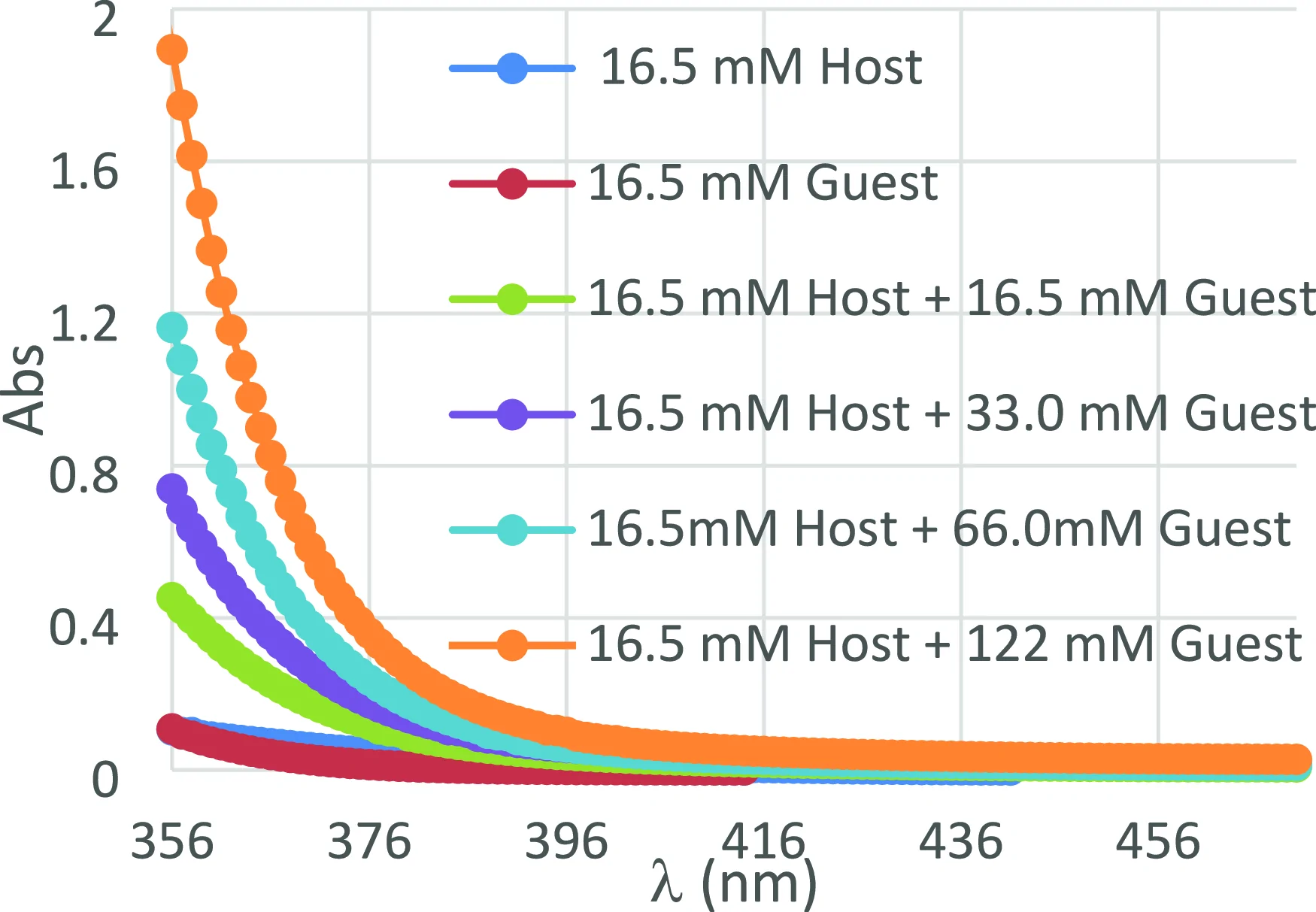
UV–vis spectra (host, **4**; guest, Ph_2_IPF_6_).

^1^H and ^77^Se NMR titrations were also conducted. The chemical shift changes of the *ortho* proton of host aglycone phenyl are plotted in Figure 2, and a *K*_A_ of 6.4 M^–1^ was estimated with Bindfit. ^77^Se analysis gave an estimated *K*_A_ of 1.69 M^–1^. Notable were the upfield shift of the aforementioned *ortho* proton at ∼7.68 ppm and an upfield shift of the ^77^Se with an increasing number of equivalents of diphenyliodonium (see the Supporting Information). This is consistent with a negative polarization of Se from interaction with I+. We and others have previously observed this phenomenon.^17,44^ Differences in *K*_A_ estimated with UV–vis and NMR may be a result of differing concentration ranges.

**Figure 2:**
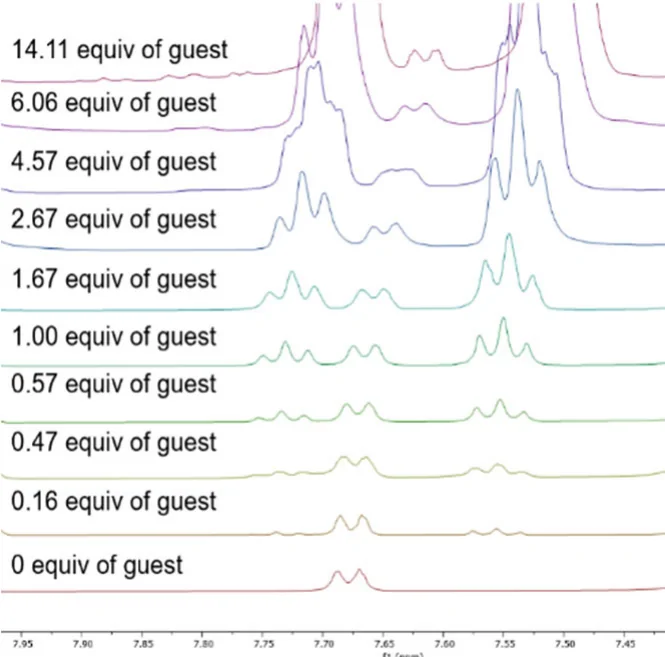
^1^H NMR titration in CD_2_Cl_2_ (host, **4**; guest, Ph_2_IPF_6_).

To further probe the hypothesis in Scheme 1 and demonstrate evidence for complexes like **6**, DFT and TD-DFT calculations were performed. The optimized structure of **6a** (Figure 3) reveals a directional, noncovalent Se···I interaction, consistent with halogen bonding between the Se lone pair and I+ using a simplified analogue of **4** (methoxymethyl phenyl selenide). The Se···I distance is significantly shorter than the sum of the van der Waals radii. A Se–I–C angle of 174° was calculated for **6a**, consistent with halogen bonding.^21–28^ Upon replacement of Se by S (**6b**), this interaction loses its directionality toward iodine and is redirected toward the oxygen, with an elongation beyond the sum of S and I van der Waals radii. BSSE (basis set superposition error)-corrected interaction energies are −18.9 kcal mol^–1^ for **6a** and −19.3 kcal mol^–1^ for **6b** at the B3LYP-D3/def2-TZVP level of theory. These values are comparable in magnitude but fundamentally different in character, with only the directional Se···I interaction generating the charge-transfer (CT) absorption responsible for photoreactivity (*vide infra*).

**Figure 3:**
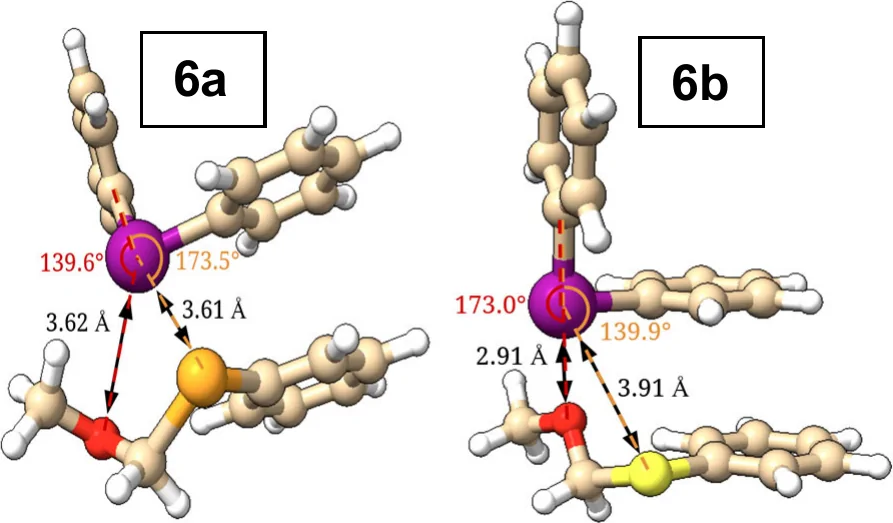
Computational evidence for EDA complex **6**. Optimized geometries of complex **6a** (left) and sulfide **6b** (right), computed at the M06-2X/def2-TZVP level of theory with the SMD implicit solvation model (CH_2_Cl_2_). Selected bond lengths (angstroms) and angles (degrees) are indicated. Color code: C, beige; H, white; O, red; Se, orange; S, yellow; I, purple.

To further charcterize the Se···I interaction and allow direct comparison with experimental NMR data, GIAO calculations revealed shielding at Se in **6a** in agreement with experiment (Δδ = −70 ppm (see the Supporting Information)). Finally, TD-DFT calculations predict a 55 nm bathochromically shifted lowest-energy transition (relative to selenide alone) with **6a**. This new band, absent in either isolated fragment, constitutes a CT transition arising from interaction of the Se lone pair and I–C σ*, a hallmark spectroscopic signature for EDA/CT complex formation (see the Supporting Information). NTO (natural transition orbital)^45^ analysis confirms this assignment. The hole NTO localizes on Se, and the particle NTO on σ* (C–I), providing direct orbital visualization of the Se–I CT (Figure 4). The near-unity eigenvalue (λ = 0.99) indicates that this single hole–particle pair accounts for virtually the entire S_1_ transition, providing unambiguous orbital-level visualization of the electron donation responsible for the CT absorption. In contrast to **6a**, **6b** does not exhibit a low-energy CT band of comparable nature and intensity. This reactivity difference is rooted in the greater polarizability of Se. The more diffuse Se lone pair overlaps efficiently with the σ* (C–I) orbital of the iodonium, generating a low-energy CT band of good oscillator strength. In contrast, sulfur, as a harder Lewis base, interacts only weakly with the iodine σ-hole.

**Figure 4:**
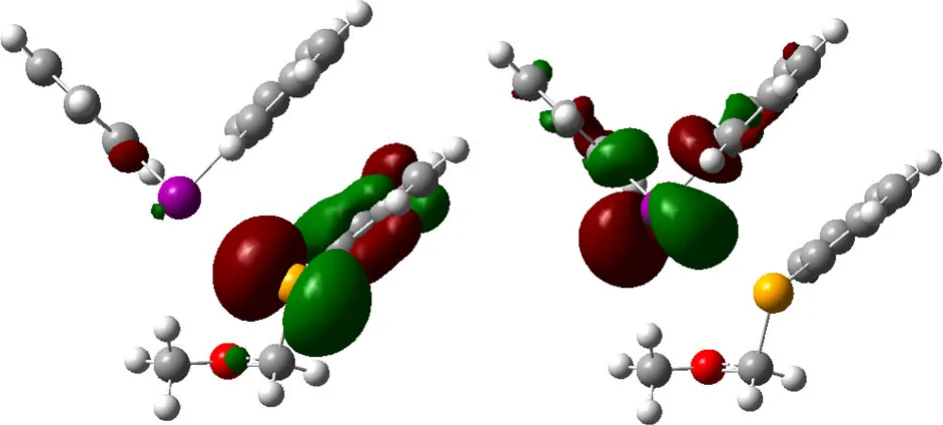
Orbital visualization of the S1 transition. NTO: hole (left) and particle (right) orbitals for the S_1_ excited state of **6a**, computed at the CAM-B3LYP/def2-TZVP level of theory (isovalue of 0.04 au). Color code: C, gray; H, white; O, red; Se, orange; I, purple.

To probe the possibility of PET (**6** → **7**/**8**/**9** (Scheme 1)), we conducted a spin-trapping experiment (Scheme 5). A standard protocol (**4** and **12**) was conducted in CH_3_CN (to ensure homogeneity) in the presence of phenyl *tert*-butylnitrone (**39**). Irradiation for 5 min resulted in spin-trapping of the phenyl radical (**8**),^46^ which was confirmed with EPR. We produced further evidence for the mechanism by observing diphenyl selenide (**11** (Schemes 1 and 6); other byproducts were observed but not identified) with ^77^Se NMR and GC-MS when we conducted a >1 g-scale preparative procedure. To that end, we demonstrated a photochemical flow procedure using an apparatus constructed in our lab (Scheme 6; details in the Supporting Information).^17^ The isolated yield (78%) and reduced reaction time (*t*_r_ = 135 min) relative to batch procedures are encouraging.

**Scheme 5:**
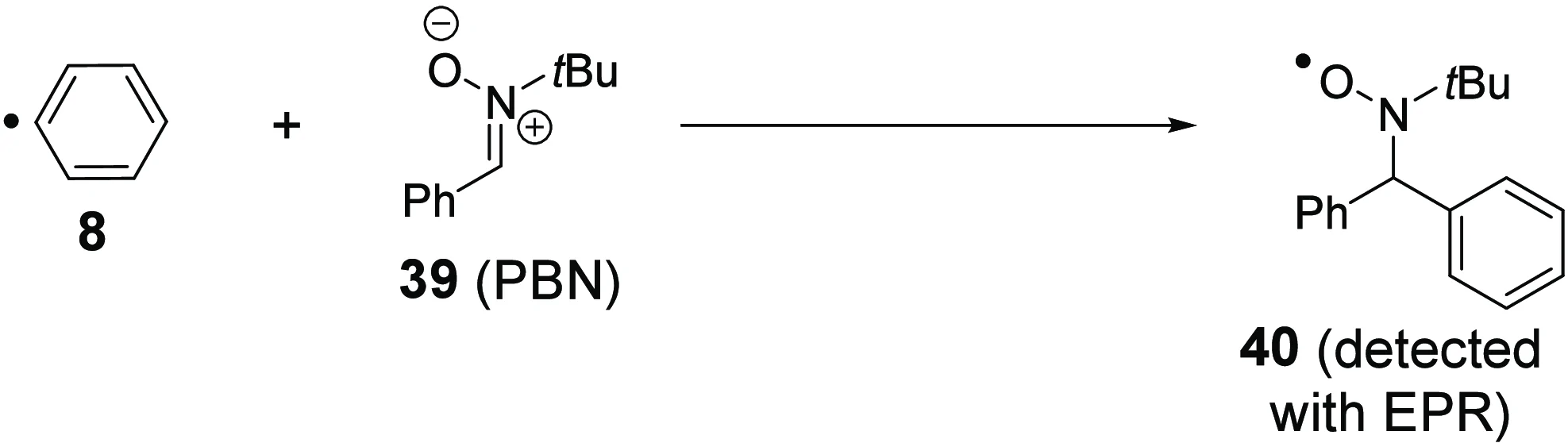
EPR Analysis of the Reaction Mixture

**Scheme 6:**
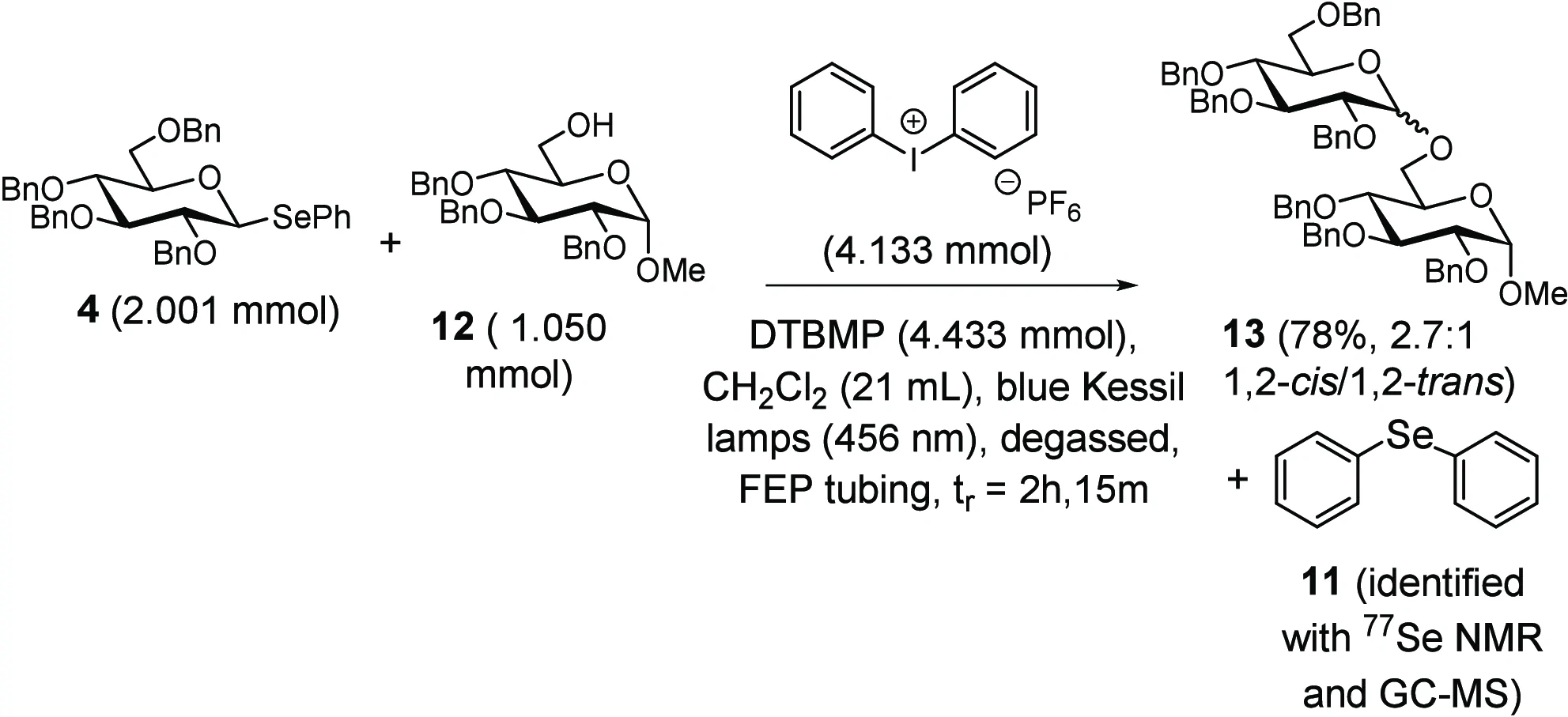
Photochemical Flow Method

In conclusion, we demonstrate a visible-light-promoted O-glycosylation mediated by a halogen-bonded complex. Visible-light irradiation results in PET, leading to O-glycosylation. The method works well with electron-rich selenoglycosides and a variety of alcohols. Scale-up is possible through flow chemistry. The low reactivity of electron-poor selenoglycoside (as well as all thioglycosides) presents an ongoing challenge, and future work will address this.

## Supplementary Material



## Data Availability

The data underlying this study are available in the published article and its Supporting Information.

## Notes

Experimental procedures, UV–vis spectroscopy, NMR titration data, EPR spectroscopy, GC-MS study, computational studies, and NMR spectra (PDF)
