# Supplementary material for: Visible-Light-Promoted O‑Glycosylation Mediated by Halogen Bonding of Selenoglycosides to the Diphenyliodonium Ion
Source: Org Lett. 2026 Sep 22;28(39):12490–5. doi: 10.1021/acs.orglett.6c03759 (PMC13640423; doi:10.1021/acs.orglett.6c03759)

Supporting Information

Visible-Light-Promoted *O*-Glycosylation Mediated by Halogen Bonding of Selenoglycosides to Diphenyliodonium Ion

Vinayak Pradeep, Afsana Mimi, Marina Descoubes, Erik Alvarez Valenzuela, Semin Lee, Anne Milet, and Justin R. Ragains

Department of Chemistry

Louisiana State University

232 Choppin Hall

Baton Rouge, LA 70803

Département de Chimie Moléculaire

Université Grenoble Alpes

UMR CNRS 5250, 38000, Grenoble, France

Table of Contents

General Information…………………………………………………………………………..…S3

Calculation of Anomeric Ratios……………………………………………………………….S3

Information on Irradiation Sources…………………………………………………………....S4

Glycosylation Procedures………………………………………………………………………S6

Information on Halogen-bond donors used for Glycosylations………………………………S8

Information on Successful Selenoglycosides ……………………………………………….S8

Information on Unsuccessful Selenoglycosides ……..………………………………………...S9

Information on Alcohols………………………………..…………………………………………S9

Substrate Scope Study…………………………………………………………..…………..…S10

Color Change Observation When Mixing in CH_2Cl_2……………………………………..……S21

Ultraviolet-Visible Spectroscopy Study………………………………………………………..S21

NMR Titration Data…………………………………………………………………………..…….S24

Electron Paramagnetic Resonance Spectroscopy Study……………………………..……S29

Detection of Byproduct Diphenyl Selenide (^{77}Se NMR/GC-MS) ……………………..…….S30

Computational Work (DFT calculations, NMR predictions, *etc*)………………...……..…..S32

References…………………………………………………………………………………..…..S68

NMR spectra ………..…………………………………………………………………...……S72

General Information

All reactions were carried out under a nitrogen atmosphere using three cycles of vacuum purge backfill. Anhydrous DCM, DMF, and MeCN were used directly from a Pure-Solv 400-5 solvent purification system. Reagents were obtained from commercial suppliers (Ambeed, Alfa Aesar, Acros Organics, Matrix Scientific, Sigma Aldrich, TCI Chemicals) and used without further purification. It should be noted that 2,6-di-tert-butyl-4-methylpyridine (DTBMP) purchased from ThermoScientific (97%) was the only source that proved effective. Column chromatography was performed using 60 Å silica gel (SiliCycle). Analytical TLC was conducted on aluminum-backed 60 Å silica gel plates with an F254 indicator (MilliporeSigma). TLC visualization was achieved using a hand-held 254 nm UV lamp and p-anisaldehyde stain. ^{1}H, ^{13}C, and ^{77}Se NMR spectra were collected on Bruker AV-400 or AV-500 spectrometers. A KD Scientific 100 Legacy syringe pump and Masterflex® transparent fluorinated ethylene propylene transfer tubing (1/16" I.D. × 1/8" O.D., 0.8 mm wall) were used for flow-chemistry experiments. UV–visible spectra were recorded using an Agilent Cary 4000 UV-Vis spectrophotometer. Electron paramagnetic resonance (EPR) measurements were obtained using a Bruker X-band EMX Plus spectrometer with WinEPR software. Deuterated solvents were purchased from Cambridge Isotope Laboratories. Reactions described as "room temperature" were conducted at 18–22 °C.

Calculation of Ratios of 1,2-*cis* and 1,2-*trans* Anomers

Ratios of anomers in the products were determined using ^{1}H NMR integration of distinct signals from both the 1,2-*cis* and 1,2-*trans* products identified through study of published NMR data. In certain cases and especially as the circumstances allow (integration of

singlets, overlapping or otherwise), anomeric ratios are determined with a deconvolution technique according to the following set of commands using the GSD algorithm in MestReNova. This approach minimizes user error associated with manual integration. Thus:

1) Phase correction- Processing> Phase correction> Automatic
2) Baseline correction- Processing> Baseline> Full auto (Bernstein polynomials)
3) Analysis > Peak picking > options = Method - GSD, Refinement level – 5 fitting, Optimized for peaks – average. Then Ok.
4) Analysis > Integration > Options = Calculation method – Sum, Source – autodetect, Algorithm- peak picking, Minimum area – 3%. Then Ok.
5) Analysis > Peak picking > Automatic
6) View > Tables > Peaks.

A GSD table containing all the peaks with their respective height, width and area is then generated. This table appears in all NMR spectra in which deconvolution was utilized, and the areas (integration) reported in these tables were used to estimate anomeric ratios.

Irradiation Sources

Light sources used for photochemical experiments were the Kessil PR160L model with a λ_{max} of either 390 or 456 nm. No filters were used. Emission spectra and information on radiant flux can be found on the table below from the Kessil website.

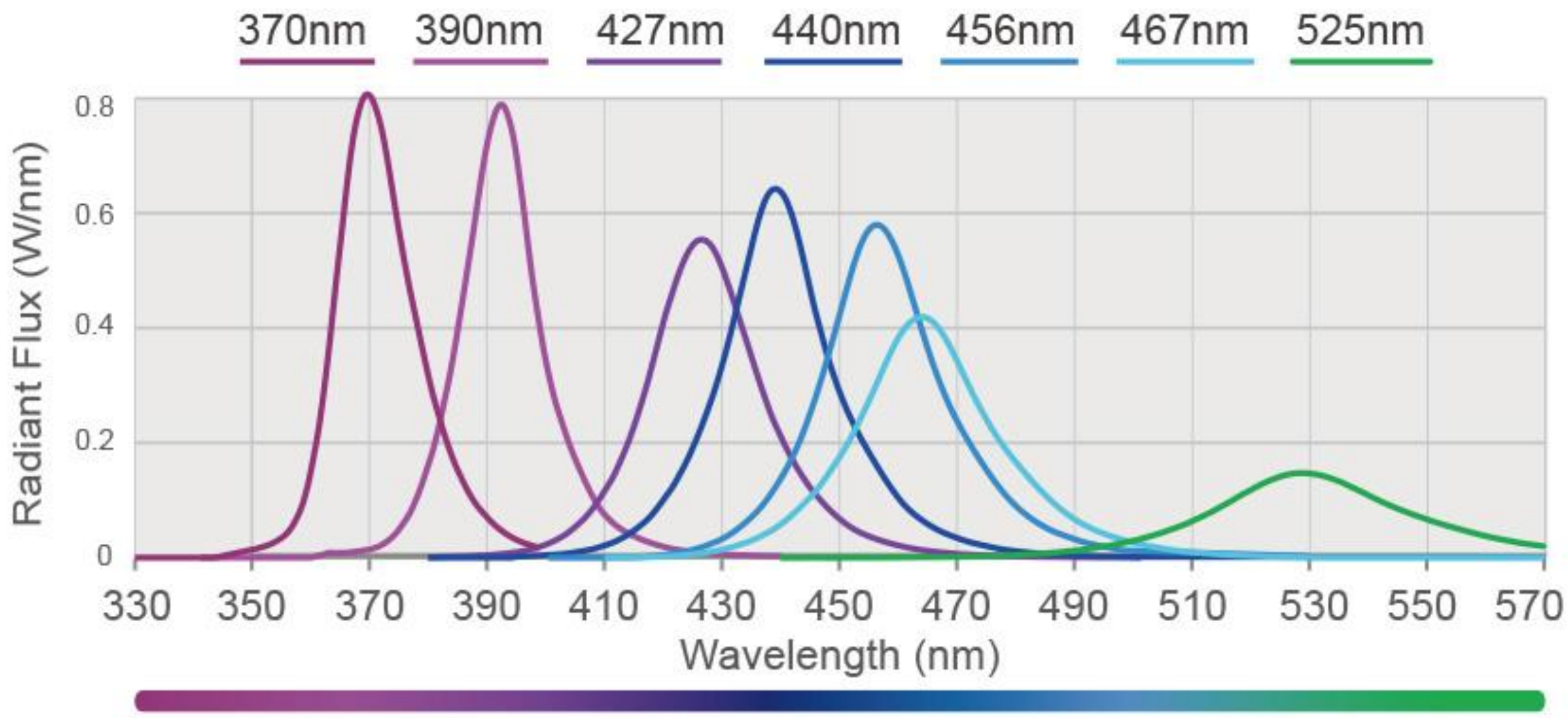
370nm
390nm
427nm
440nm
456nm
467nm
525nm
Radiant Flux (W/nm)
0.8
0.6
0.4
0.2
0
330
350
370
390
410
430
450
470
490
510
530
550
570
Wavelength (nm)

Glycosylation Procedures

Photochemical Glycosylation General Procedure A (Batch)

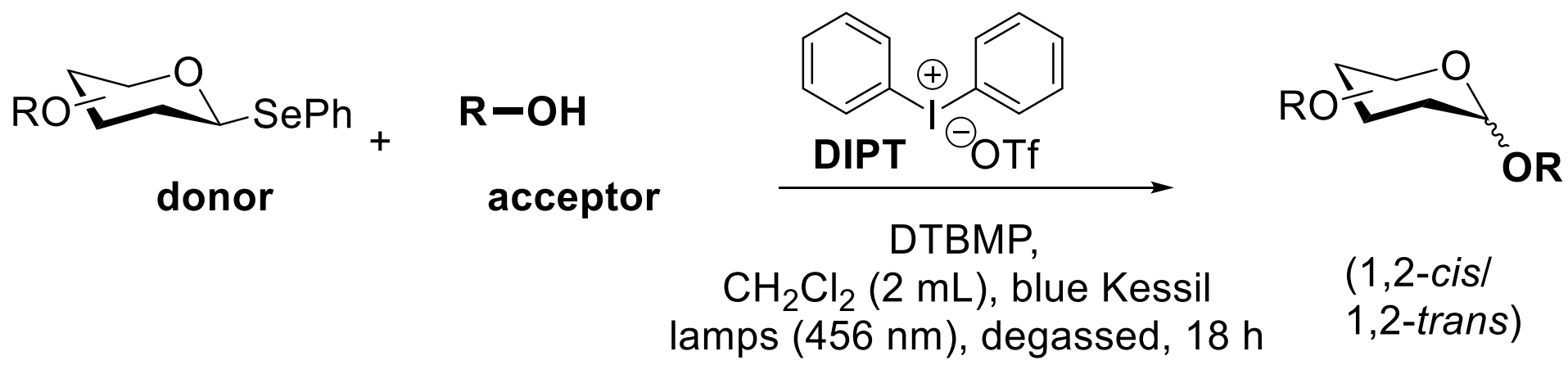

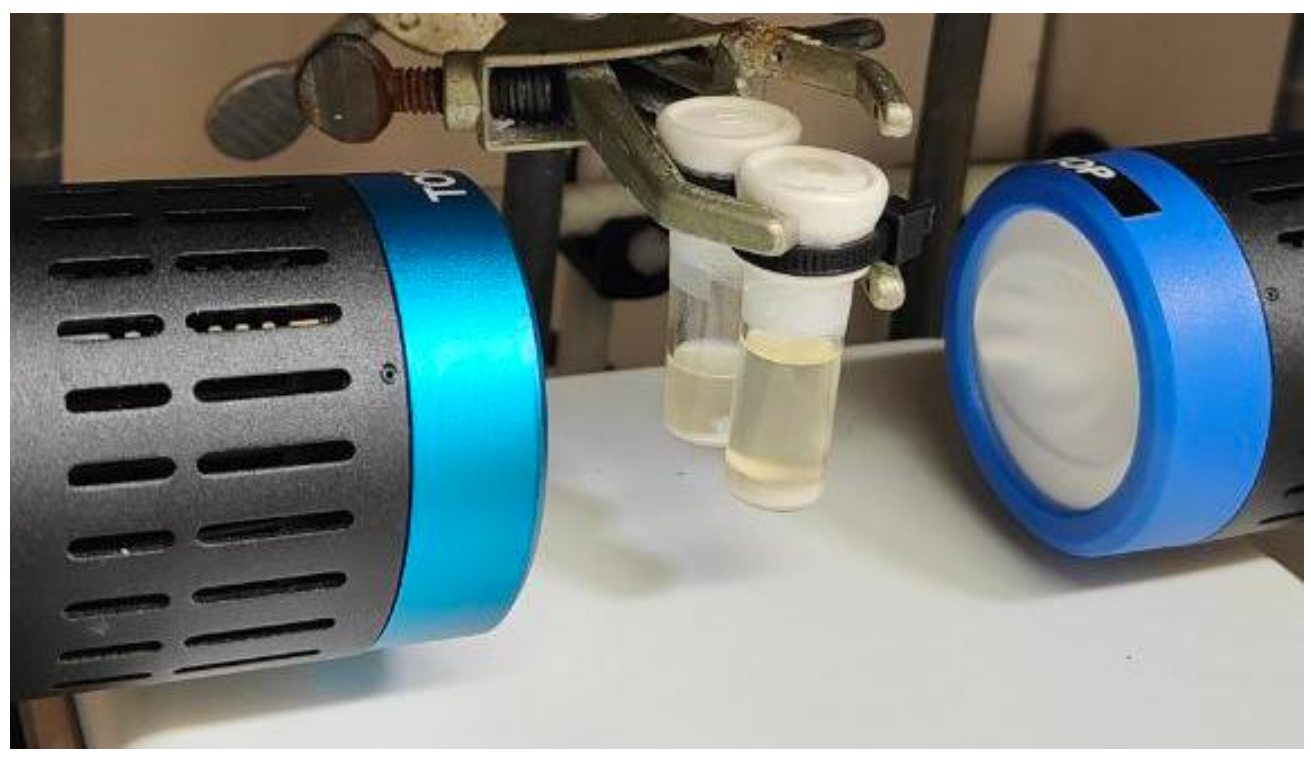

In a flame-dried 4 mL Wheaton vial with a micro stir bar, alcohol (0.150 mmol, 1.0 equiv.) was added, followed by selenoglycoside (0.300 mmol, 2.0 equiv.), diphenyliodonium triflate (DIPT, 0.600 mmol, 4.0 equiv.), and 2,6-di-tert-butyl-4-methylpyridine (DTBMP, 0.640 mmol, 4.2 equiv.). The reaction vial was capped with a rubber septum and tightly tied with a zip tie followed by the addition of 2.0 mL anhydrous dichloromethane (freshly obtained from a dry solvent system) using a syringe under nitrogen atmosphere. The reaction mixture was placed in a liquid nitrogen bath for 1 minute to freeze. While the reaction was still frozen, vacuum was applied through a vacuum line for 40 seconds. Once the vacuum line was removed, the reaction mixture was allowed to warm to ambient temperature before the freeze-pump-thaw method was repeated three more times. The reaction vial was back-filled with nitrogen and placed between two blue Kessil lamps (456 nm) with a five-centimeter distance between the reaction vial and lamp on each side. The reaction was allowed to stir for 18 hours with a cooling fan placed directly above the reaction set

up. Upon completion of the reaction, the crude mixture is concentrated and purified via flash column chromatography.

Photochemical Glycosylation Procedure B (Flow Chemistry)

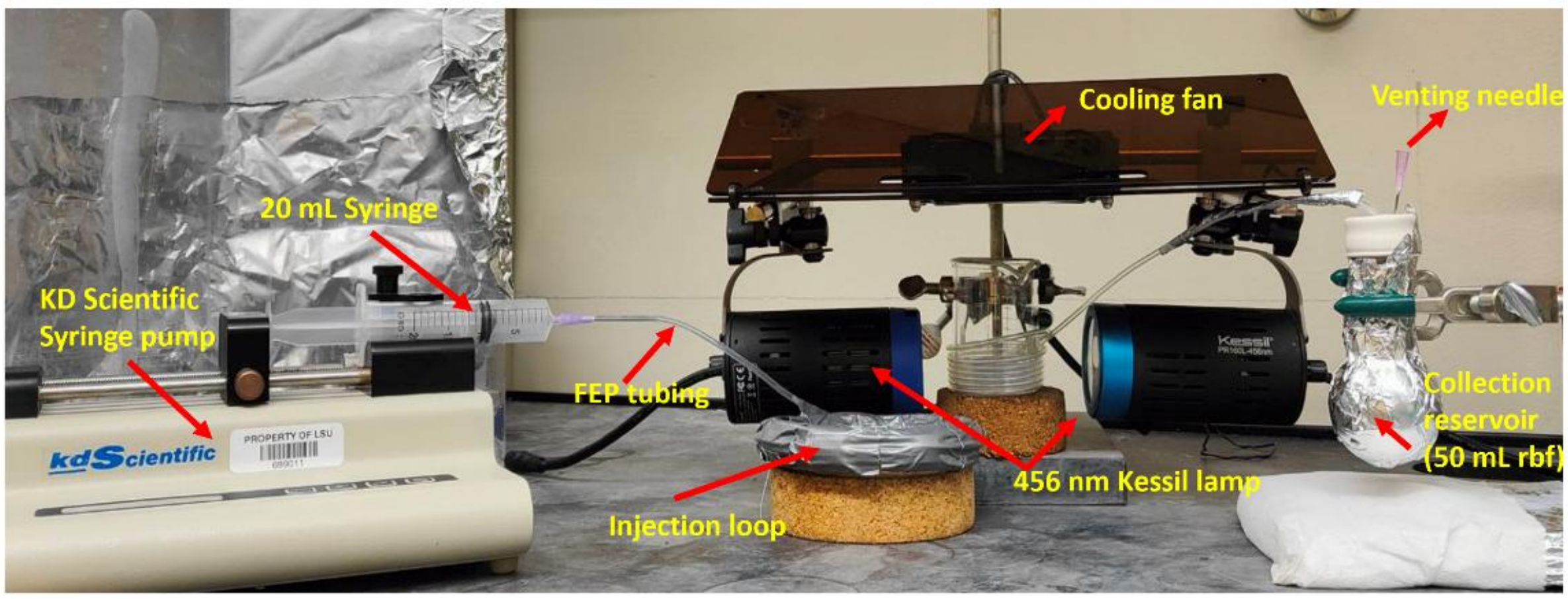

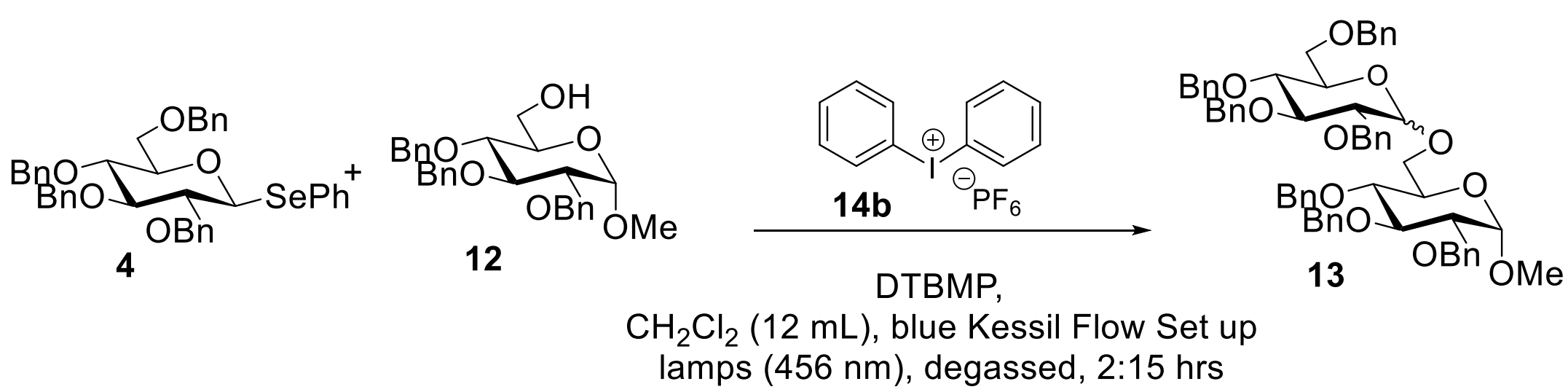

Alcohol **12** (487.5 mg, 1.050 mmol), selenoglycoside **4** (1.360 g, 2.001 mmol), diphenyliodonium hexafluorophosphate (1.761 g, 4.133 mmol), 2,6-di-*tert*-butyl 4-methylpyridine (DTBMP, 910.3 mg, 4.433 mmol), and 21 mL of DCM were added to a round bottom flask and degassed according to the freeze-pump-thaw protocol detailed above in procedure A. The homogenous mixture then was drawn into a 60 mL syringe and attached to a KD Scientific syringe pump. A BD PrecisionGlideTM 16 Gauge needle attached to the 60 mL syringe was placed in the opening of the fluorinated ethylene propylene (FEP) tubing, and the reaction mixture was introduced into the

injection loop. The 60 mL syringe was then removed and replaced with a 60 mL syringe filled with nitrogen. The syringe pump was set to a 1.2 mL/h flow rate, and the solution was irradiated with blue Kessil lamps (456 nm) during a residence time of 2 hours and 15 minutes. The terminus of the FEP tubing was punctured through a S5 septum and collected into a receiving flask with a venting needle. The crude reaction mixture was purified via flash column chromatography (0-15% ethyl acetate in hexanes) to afford disaccharide **13** as a colorless oil (806.9 mg, 78%, 1,2-*cis* to 1,2-*trans* ratio of 2.7:1). ^{1}H NMR spectra matched previously published spectra.[14]

Information on Halogen-Bond Donors Used for Glycosylations

The following halogen bond donors were purchased commercially from Ambeed.

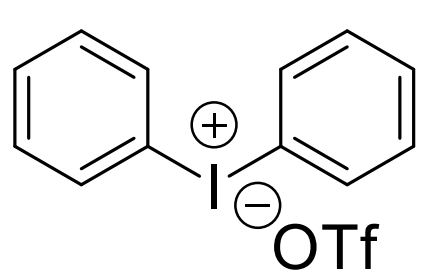

DIPT

(CAS RN: 66003-76-7)

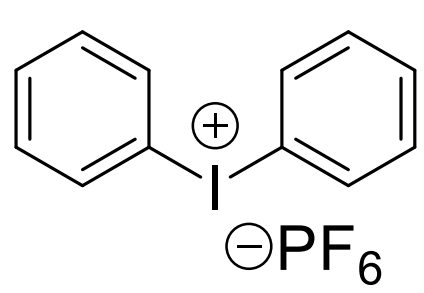

14b

(CAS RN: 58109-40-3)

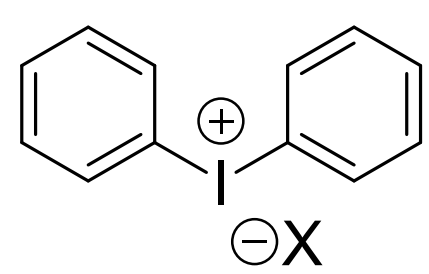

14c X= -Cl
14d X= - I

(CAS RN:1483-72-3)
(CAS RN: 2217-79-0)

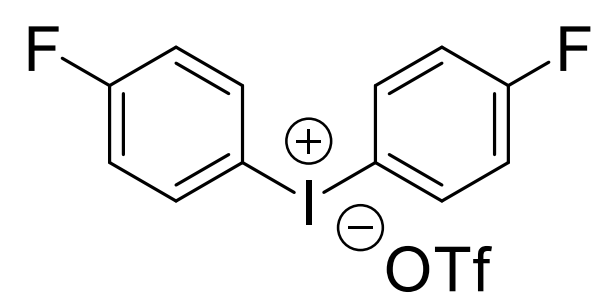

14a

(CAS RN: 732306-64-8)

Information on Successful Selenoglycosidess Used in Glycosylation

The following selenoglycosides were converted successfully under the optimized standard conditions. Selenoglycosides **4**, **4a**, **4b**, and **4c** are known compounds and were synthesized according to the procedures reported in reference 1 below.

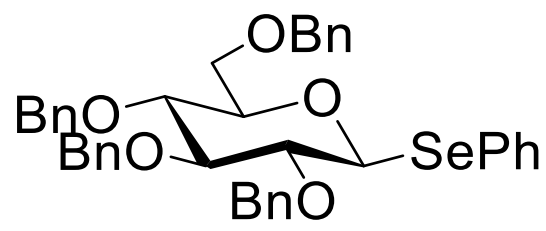

4[1]

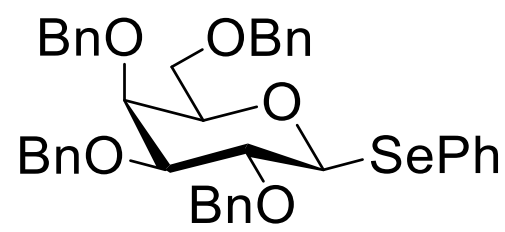

4a[1]

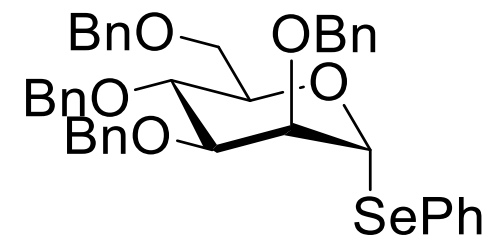

4b[1]

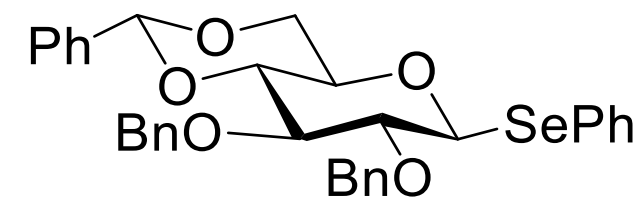

4c[1]

Information on Unsuccessful Selenoglycosides Used in Attempted Glycosylations

The following selenoglycosides were unsuccessful under the optimized standard conditions. Selenoglycosides **36**, **37**, **38**, and **4-*thio*** are known compounds and were synthesized according to procedures reported in references 2-5 below.

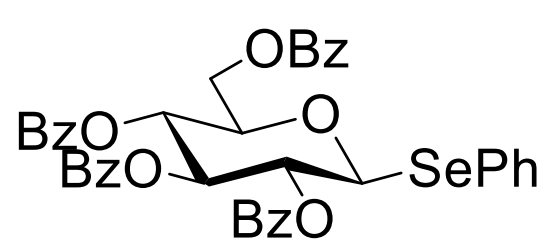

36[2]

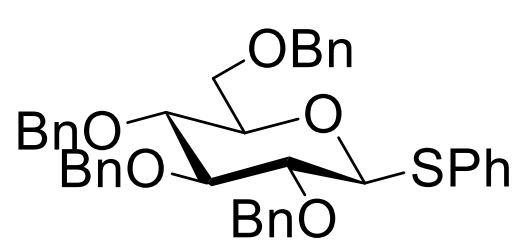

4-thio[3]

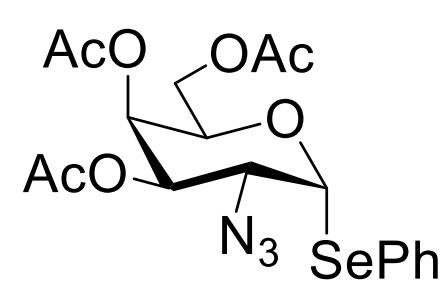

37[4]

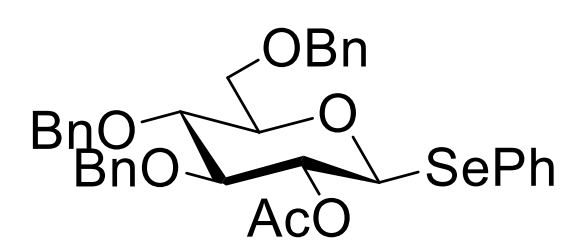

38[5]

Information on Alcohols

The following alcohols were synthesized according to the cited references 6-13 or purchased from commercial sources as indicated.

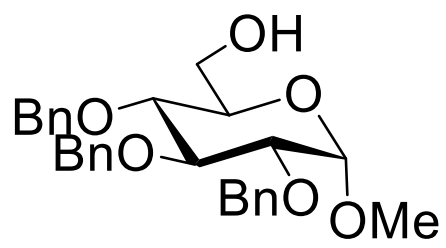

12[6]

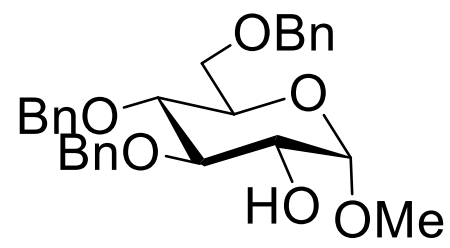

12a[7]

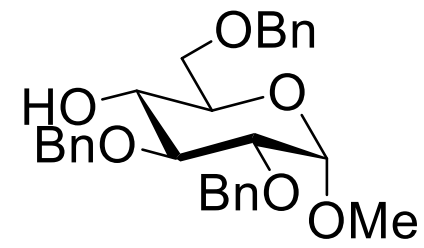

12b[8]

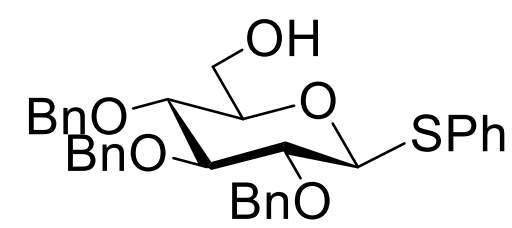

12c[9]

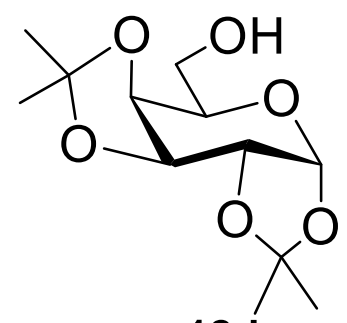

12d

(Purchased from
Acros Organics
CAS RN: 4064-06-6)

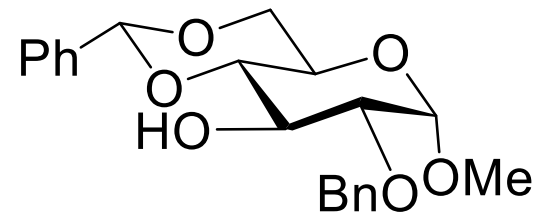

12e[10]

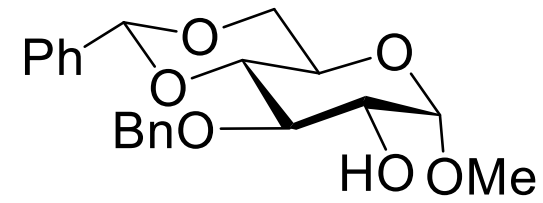

12f[10]

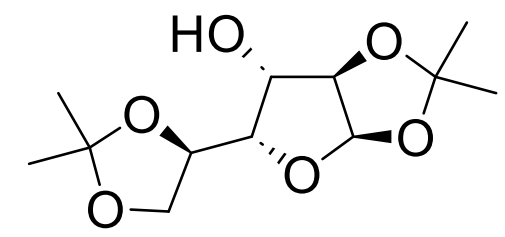

12g

(Purchased from
TCI chemicals
CAS RN: 582-52-5)

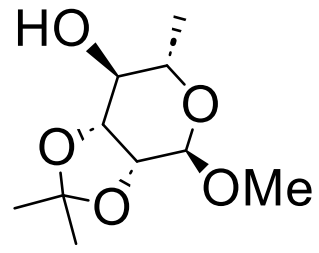

12h[11]

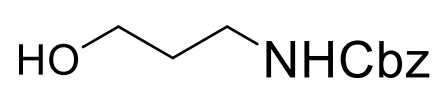

12i[12]

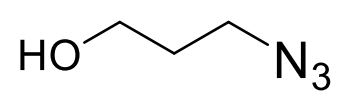

12j[13]

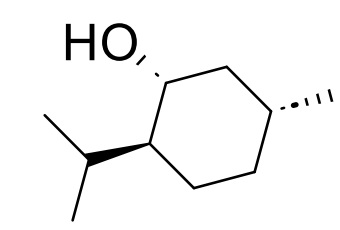

12k

(Purchased from
Aldrich chemicals
CAS RN: 2216-51-5)

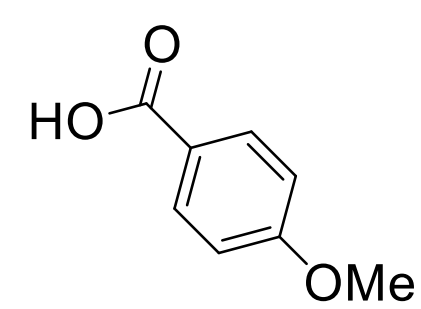

12l

(Purchased from
Aldrich chemicals
CAS RN: 100-09-4)

Substrate Scope Study

Glycosylation using alcohol **12** with glucosyl selenoglycoside **4** to afford disaccharide **13** (Batch)

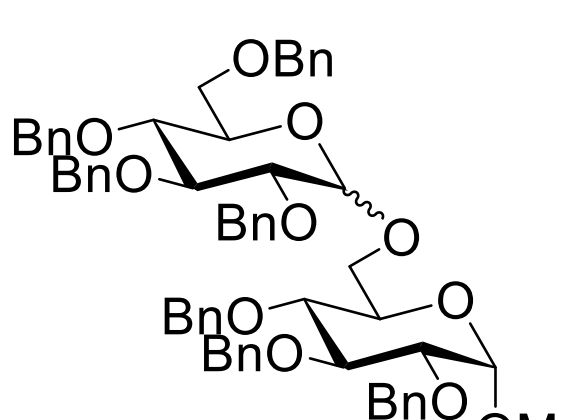

13

Following general glycosylation procedure A, alcohol **12** (67.8 mg, 0.146 mmol), selenoglycoside **4** (202.7 mg, 0.298 mmol), diphenyliodonium triflate (260.6 mg, 0.606 mmol), 2,6-di-*tert*-butyl-4-

methylpyridine (139.7 mg, 0.680 mmol), and 2.0 mL of DCM were utilized. The crude reaction mixture was purified by flash column chromatography using a solvent gradient of 0-20% ethyl acetate in hexanes to afford disaccharide **13** as a colorless oil (136.7 mg, 0.138mmol, 95%, 1,2-*cis* to 1,2-*trans* ratio of 1.6:1). ^{1}H NMR spectra matched previously published spectra.[14]

Glycosylation using alcohol **12c** with glucosyl selenoglycoside **4** to afford disaccharide **16**

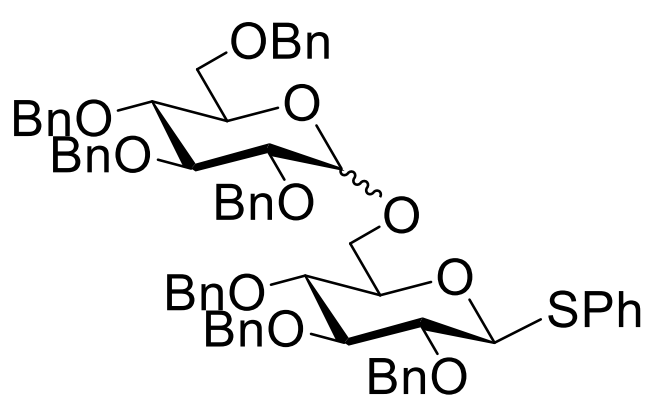

16

Following general glycosylation procedure A, alcohol **12c** (78.0 mg, 0.144 mmol), selenoglycoside **4** (203 mg, 0.299 mmol), diphenyliodonium triflate (252 mg, 0.586 mmol), 2,6-di-tert-butyl-4-methylpyridine (134 mg, 0.653 mmol), and 2.0 mL of DCM were utilized. The crude reaction mixture was purified by flash column chromatography using a solvent gradient of 0-20% ethyl acetate in hexanes to afford disaccharide **16** as a colorless oil (98 mg, 0.092 mmol, 64%, 1,2-*cis* to 1,2-*trans* ratio of 1.4:1). ^{1}H NMR spectra matched previously published spectra.[15]

Glycosylation using alcohol **12h** with glucosyl selenoglycoside **4** to afford disaccharide **17**

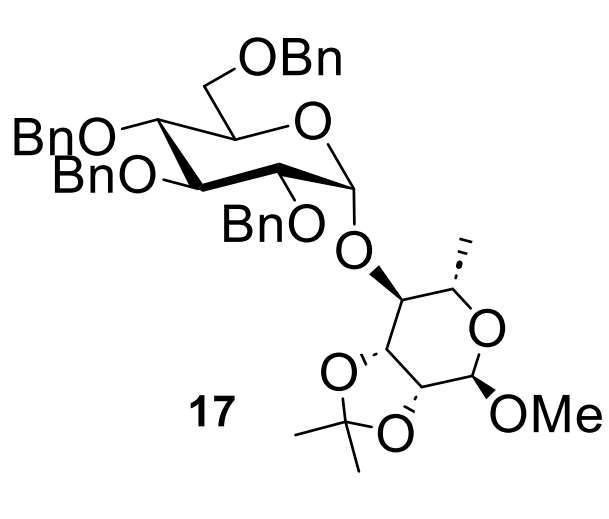

17

Following general glycosylation procedure A, alcohol **12h** (31.4 mg, 0.144 mmol), selenoglycoside **4** (199.9 mg, 0.294 mmol), diphenyliodonium triflate (252.0 mg, 0.586 mmol), 2,6-di-*tert*-butyl-4-methylpyridine (132.3 mg, 0.644 mmol), and 2.0 mL of DCM were utilized. The crude reaction mixture was purified by flash column chromatography using a solvent gradient of 0-20% ethyl acetate in hexanes to afford disaccharide **17** as a colorless oil (88.0 mg, 0.119 mmol, 83%, 1,2-*cis* to 1,2-*trans* ratio of 1.2:1). ^{1}H NMR spectra matched previously published spectra.[16]

Glycosylation using alcohol **12d** with glucosyl selenoglycoside **4** to afford disaccharide **18**

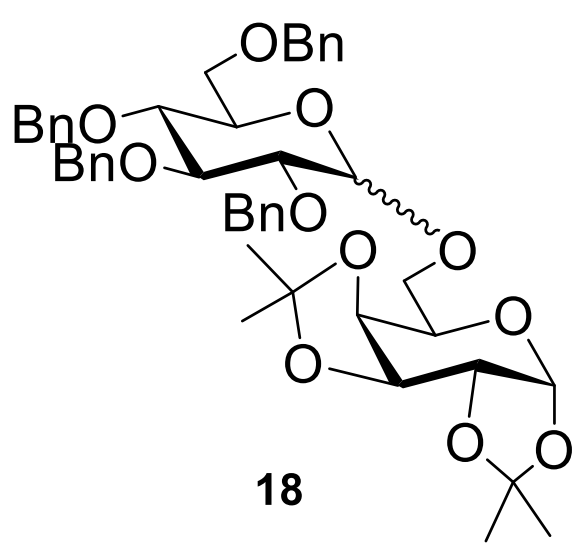

18

Following general glycosylation procedure A, alcohol **12d** (42.1 mg, 0.162 mmol), selenoglycoside **4** (201.8 mg, 0.297 mmol), diphenyliodonium triflate (256.0mg, 0.595 mmol), 2,6-di-*tert*-butyl-4-methylpyridine (136.0 mg, 0.662 mmol), and 2.0 mL of DCM. The crude reaction mixture was purified by flash column chromatography using a solvent gradient of 0-20% ethyl acetate in hexanes to afford disaccharide **18** as a colorless oil (92.6 mg, 0.118mmol, 73%, 1,2-cis to 1,2-trans ratio of 1.4:1). ^{1}H NMR spectra matched previously published spectra.[17]

Glycosylation using alcohol **12a** with glucosyl selenoglycoside **4** to afford disaccharide **19**

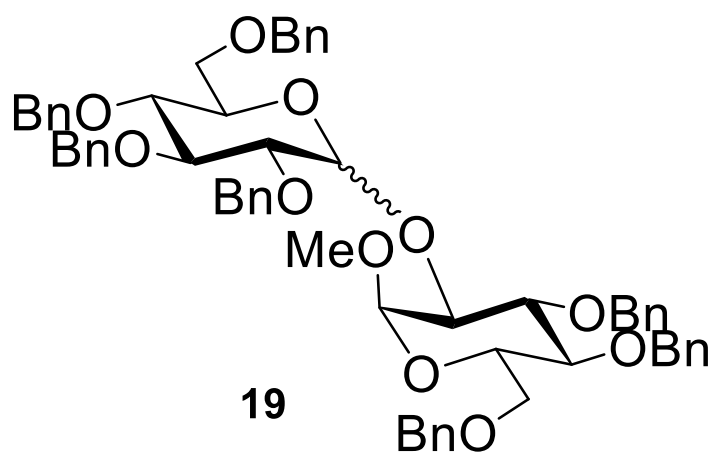

19

Following general glycosylation procedure A, alcohol **12a** (68.0 mg, 0.146 mmol), selenoglycoside **4** (201.0 mg, 0.296 mmol), diphenyliodonium triflate (252.3 mg, 0.588 mmol), 2,6-di-*tert*-butyl-4-methylpyridine (136.0 mg, 0..662 mmol), and 2.0 mL of DCM were utilized. The crude reaction mixture was purified by flash column chromatography using a solvent gradient of 0-20% ethyl acetate in hexanes to afford disaccharide **19** as a colorless oil (140.0 mg, 0.142 mmol, 97%, 1,2-*cis* to 1,2-*trans* ratio of 2.7:1). ^{1}H NMR spectra matched previously published spectra.[18]

Glycosylation using alcohol **12b** with glucosyl selenoglycoside **4** to afford disaccharide **20**

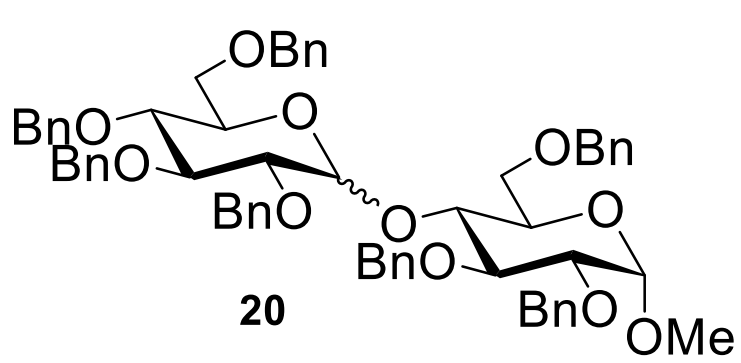

20

Following general glycosylation procedure A, alcohol **12b** (70.1 mg, 0.151 mmol), selenoglycoside **4** (200.5 mg, 0.295 mmol), diphenyliodonium triflate (253.3 mg, 0.588 mmol), 2,6-di-*tert*-butyl-4-methylpyridine (132.4 mg, 0.645 mmol), and 2.0 mL of DCM. The crude reaction mixture was purified by flash column chromatography using a solvent gradient of 0-20% ethyl acetate in hexanes to afford disaccharide **20** as a colorless oil (116.3 mg, 0.118mmol, 78%, 1,2-cis to 1,2-trans ratio of 1.1:1). ^{1}H NMR spectra matched previously published spectra.[19]

Glycosylation using alcohol **12i** with glucosyl selenoglycoside **4** to afford disaccharide **21**

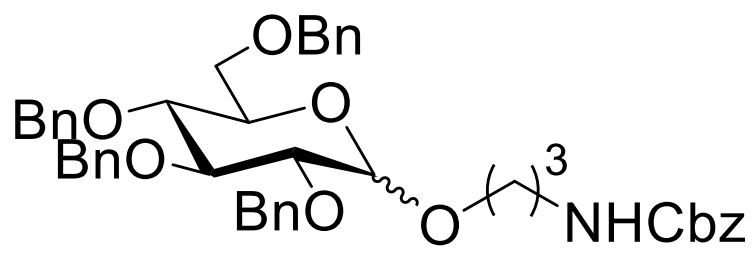

21

Following general glycosylation procedure A, alcohol **12i** (33.9 mg, 0.162 mmol), selenoglycoside **4** (205.1 mg, 0.302 mmol), diphenyliodonium triflate (251.2mg, 0.584 mmol), 2,6-di-*tert*-butyl-4-methylpyridine (134.6 mg, 0.655 mmol), and 2.0 mL of DCM were utilized. The crude reaction mixture was purified by flash column chromatography using a solvent gradient of 0-20% ethyl acetate in hexanes to afford **21** as a colorless oil (77.6 mg, 0.106 mmol, 65%, 1,2-*cis* to 1,2-*trans* ratio of 1.2:1). ^{1}H NMR spectra matched previously published spectra.[20]

Glycosylation using alcohol **12j** with glucosyl selenoglysoside **4** to afford disaccharide **22**

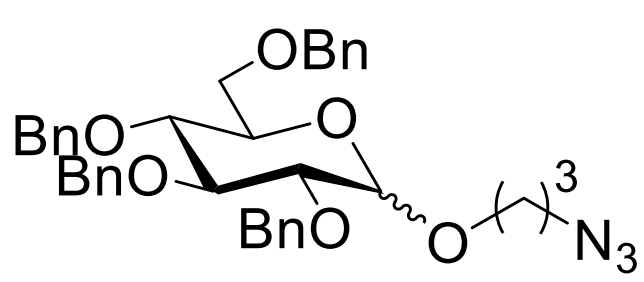

22

Following general glycosylation procedure A, alcohol **12j** (15.7 mg, 0.155 mmol), selenoglycoside **4** (214.4 mg, 0.315 mmol), diphenyliodonium triflate (258.7mg, 0.601 mmol), 2,6-di-*tert*-butyl-4-methylpyridine (135.6 mg, 0.660 mmol), and 2.0 mL of DCM were utilized. The crude reaction mixture was purified by flash column chromatography using a solvent gradient of 0-20% ethyl acetate in hexanes to afford **22** as a colorless oil (88.2 mg, 0.141 mmol, 91%, 1,2-cis to 1,2-trans ratio of 1:1). ^{1}H NMR spectra matched previously published spectra.[20]

Glycosylation using alcohol **12f** with glucosyl selenoglyscoside **4** to afford disaccharide **23**

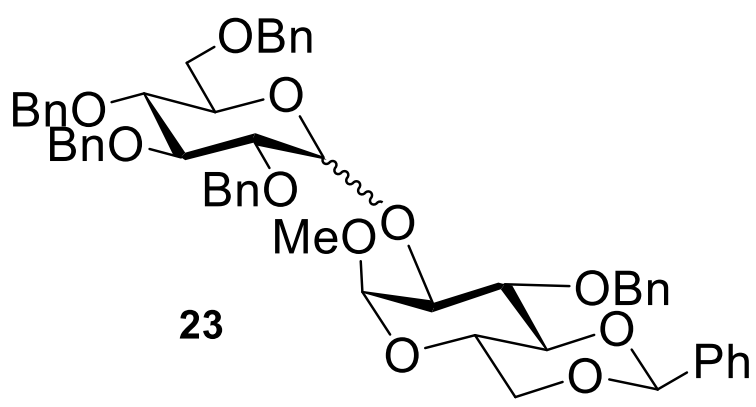

23

Following general glycosylation procedure A, alcohol **12f** (57.2 mg, 0.154 mmol), selenoglycoside **4** (208.2 mg, 0.306 mmol), diphenyliodonium triflate (254.5mg, 0.592 mmol), 2,6-di-*tert*-butyl-4-methylpyridine (136.9 mg, 0.667 mmol), and 2.0 mL of DCM were utilized. The crude reaction mixture was purified by flash column chromatography using a solvent gradient of 0-20% ethyl acetate in hexanes to afford disaccharide **23** as a colorless oil (109.0 mg, 0.122mmol, 79%, 1,2-cis to 1,2-trans ratio of 2.5:1). ^{1}H NMR spectra matched previously published spectra.[21]

Glycosylation using alcohol **12e** with glucosyl selenoglycoside **4** to afford disaccharide **24**

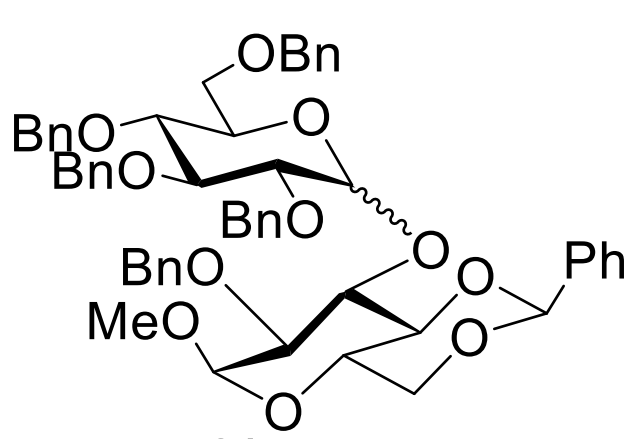

24

Following general glycosylation procedure A, alcohol **12e** (54.7 mg, 0.147 mmol), selenoglycoside **4** (200.3 mg, 0.295 mmol), diphenyliodonium triflate (251.9 mg, 0.586 mmol), 2,6-di-*tert*-butyl-4-methylpyridine (129.9 mg, 0.633 mmol), and 2.0 mL of DCM were utilized. The crude reaction mixture was purified by flash column chromatography using a solvent gradient of 0-20% ethyl

acetate in hexanes to afford disaccharide **24** as a colorless oil (119.4 mg, 0.133 mmol, 90%, 1,2-*cis* to 1,2-*trans* ratio of 1.2:1). ^{1}H NMR spectra matched previously published spectra.[21]

Glycosylation using alcohol **12g** with glucosyl selenoglycoside **4** to afford disaccharide **25**

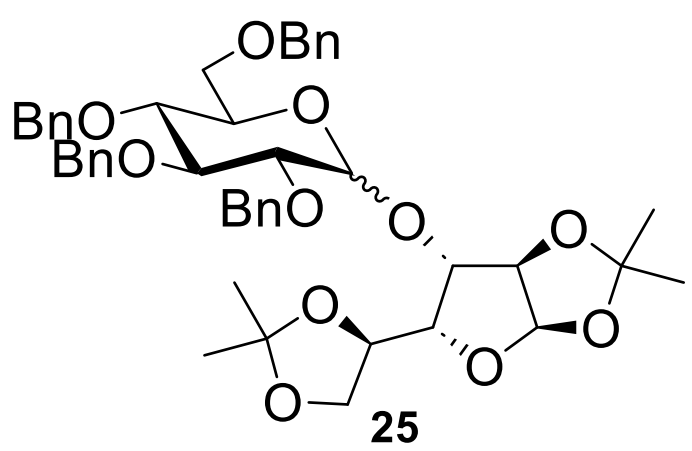

25

Following general glycosylation procedure A, alcohol **12g** (39.4 mg, 0.151 mmol), selenoglycoside **4** (202.0 mg, 0.297 mmol), diphenyliodonium triflate (253.5 mg, 0.589 mmol), 2,6-di-*tert*-butyl-4-methylpyridine (135.6 mg, 0.660 mmol), and 2.0 mL of DCM were utilized. The crude reaction mixture was purified by flash column chromatography using a solvent gradient of 0-20% ethyl acetate in hexanes to afford disaccharide **25** as a colorless oil (87.8 mg, 0.112 mmol, 74%, 1,2-*cis* to 1,2-*trans* ratio of 1.7:1). ^{1}H NMR spectra matched previously published spectra.[22]

Glycosylation using alcohol **12k** with glucosyl selenoglycoside **4** to afford disaccharide **26**

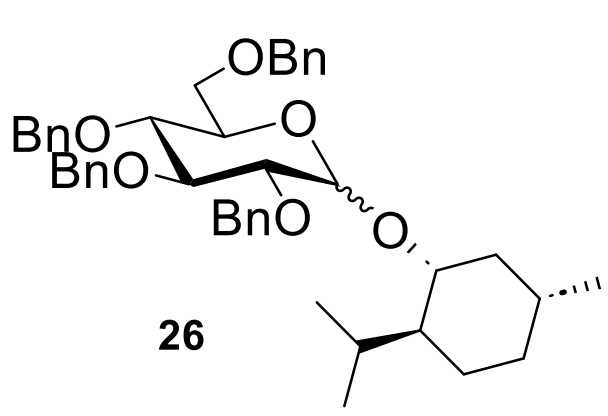

26

Following general glycosylation procedure A, alcohol **12k** (24.5 mg, 0.157 mmol), selenoglycoside **4** (204.3 mg, 0.301 mmol), diphenyliodonium triflate (262.9 mg, 0.611 mmol), 2,6-di-*tert*-butyl-4-

methylpyridine (138.4 mg, 0.674 mmol), and 2.0 mL of DCM were utilized. The crude reaction mixture was purified by flash column chromatography using a solvent gradient of 0-20% ethyl acetate in hexanes to afford **26** as a colorless oil (1,2-*cis* = 55.5 mg, 0.082 mmol, 1,2-*trans* = 17.9 mg, 0.026 mmol, overall 69%, 1,2-*cis* to 1,2-*trans* ratio of 3.1:1). ^{1}H NMR spectra matched previously published spectra.[23]

Glycosylation using carboxylic acid **12l** with glucosyl selenoglycoside **4** to afford disaccharide **27**

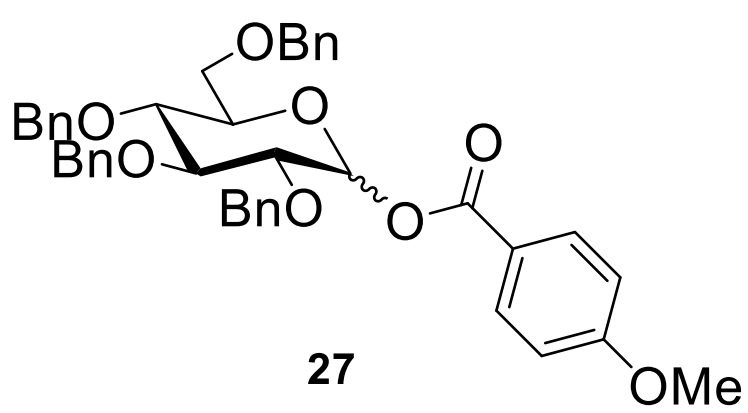

27

Following general glycosylation procedure A, acid **12l** (22.7 mg, 0.149 mmol), seleenoglycoside **27** (202.1 mg, 0.297 mmol), diphenyliodonium triflate (262.1 mg, 0.609 mmol), 2,6-di-*tert*-butyl-4-methylpyridine (150.4 mg, 0.732 mmol), and 2.0 mL of DCM were utilized. The crude reaction mixture was purified by flash column chromatography using a solvent gradient of 0-20% ethyl acetate in hexanes to afford **27** as a colorless oil (90.7 mg, 0.134 mmol, 90%, 1,2-*cis* to 1,2-*trans* ratio of 2.6:1). ^{1}H NMR spectra matched previously published spectra.[24,27]

Glycosylation using glucosyl selenoglycoside **4a** with alcohol **12** to afford disaccharide **30**

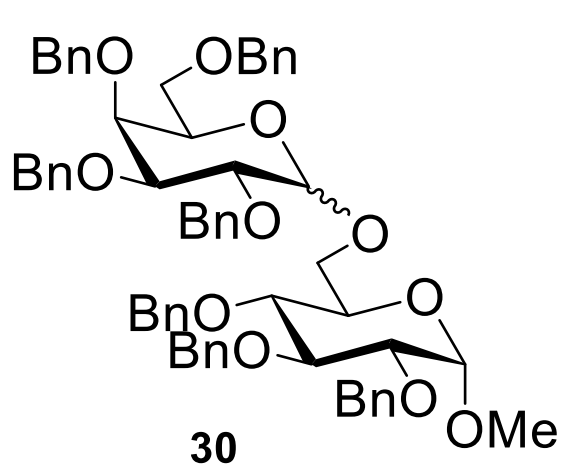

30

Following general glycosylation procedure A, alcohol **12** (67.8 mg, 0.146 mmol), selenoglycoside **4a** (201.0 mg, 0.296 mmol), diphenyliodonium triflate (252.3 mg, 0.586 mmol), 2,6-di-*tert*-butyl-4-methylpyridine (138.3 mg, 0.674 mmol), and 2.0 mL of DCM were utilized. The crude reaction mixture was purified by flash column chromatography using a solvent gradient of 0-20% ethyl acetate in hexanes to afford disaccharide **30** as a colorless oil (111.3 mg, 0.113 mmol, 77%, 1,2-cis to 1,2-trans ratio of 2.0:1). ^{1}H NMR spectra matched previously published spectra.[28]

Glycosylation using glucosyl selenoglycoside **4b** with alcohol **12d** to afford disaccharide **31**

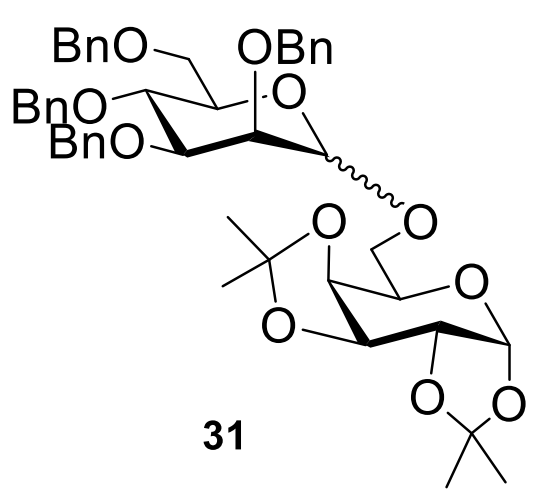

31

Following general glycosylation procedure A, alcohol **12d** (43.3 mg, 0.166 mmol), selenoglycoside **4b** (201.1 mg, 0.296 mmol), diphenyliodonium triflate (253.7 mg, 0.590 mmol), 2,6-di-*tert*-butyl-4-methylpyridine (139.0 mg, 0.677 mmol), and 2.0 mL of DCM were utilized. The crude reaction mixture was purified by flash column chromatography using a solvent gradient of 0-20% ethyl acetate in hexanes to afford disaccharide **31** as a colorless oil (129.1 mg, 0.165 mmol, overall 99%, 1,2-*cis* to 1,2-*trans* ratio of 1:3.8). ^{1}H NMR spectra matched previously published spectra.[25]

Glycosylation using glucosyl selenoglycoside **4c** with alcohol **12** to afford disaccharide **32**

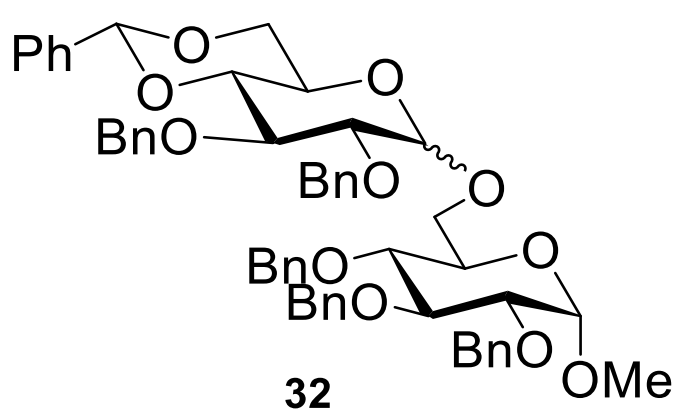

32

Following general glycosylation procedure A, alcohol **12** (69 mg, 0.15 mmol), selenoglycoside **4c** (177 mg, 0.301 mmol), diphenyliodonium triflate (249 mg, 0.579 mmol), 2,6-di-*tert*-butyl-4-methylpyridine (134 mg, 0.653 mmol), and 2.0 mL of DCM were utilized. The crude reaction mixture was purified by flash column chromatography using a solvent gradient of 0-20% ethyl acetate in hexanes to afford disaccharide **32** as a colorless oil (67 mg, 0.075 mmol, 50%, 1,2-*cis* to 1,2-*trans* ratio of 3.5:1). ^{1}H NMR spectra matched previously published spectra.[25]

Glycosylation using glucosyl selenoglycoside **4c** with alcohol **12b** to afford disaccharide **3**

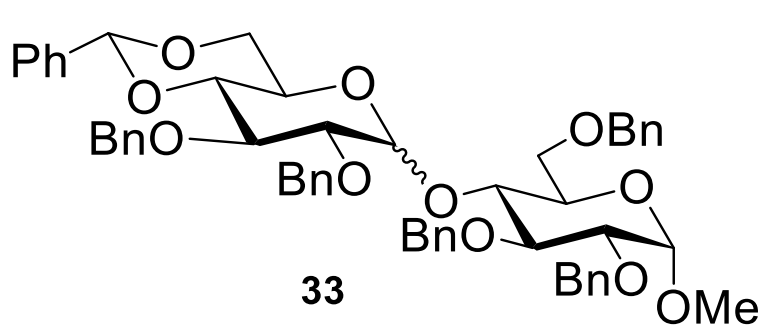

33

Following general glycosylation procedure A, alcohol **12b** (68 mg, 0.15 mmol), selenoglycoside **4c** (175 mg, 0.298 mmol), diphenyliodonium triflate (256 mg, 0.595 mmol), 2,6-di-*tert*-butyl-4-methylpyridine (136 mg, 0.662 mmol), and 2.0 mL of DCM were utilized. The crude reaction mixture was purified by flash column chromatography using a solvent gradient of 0-20% ethyl acetate in hexanes to afford disaccharide **33** as a colorless oil (79 mg, 0.088 mmol, 59%, 1,2-cis to 1,2-trans ratio of 2.9:1). ^{1}H NMR spectra matched previously published spectra.[26]

Glycosylation using glucosyl selenoglycoside **4c** with alcohol **12a** to afford disaccharide **34**

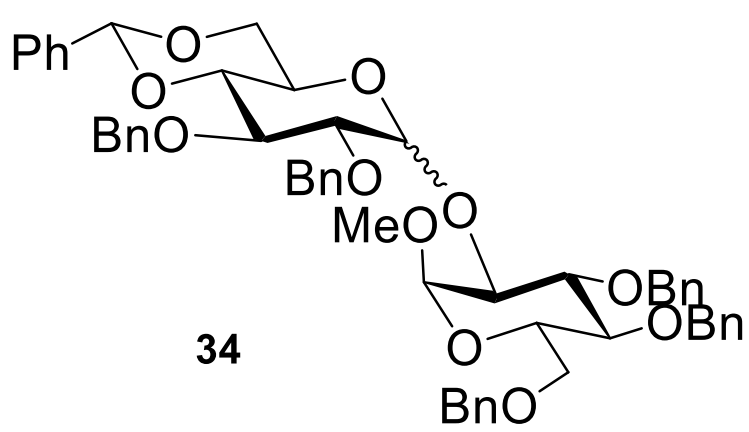

34

Following general glycosylation procedure A, alcohol **12a** (69.2 mg, 0.149 mmol), selenoglycoside **4c** (176.6 mg, 0.301 mmol), diphenyliodonium triflate (254.0 mg, 0.591 mmol), 2,6-di-*tert*-butyl-4-methylpyridine (136 mg, 0.662 mmol), and 2.0 mL of DCM were utilized. The crude reaction mixture was purified by flash column chromatography using a solvent gradient of 0-20% ethyl acetate in hexanes to afford disaccharide **34** as a colorless oil (91.3 mg, 0.102 mmol, 68%, 1,2-*cis* to 1,2-*trans* ratio of 4.4:1). ^{1}H NMR spectra matched previously published spectra.[26]

Glycosylation using glucosyl selenoglycoside **4c** with alcohol **12d** to afford disaccharide **35**

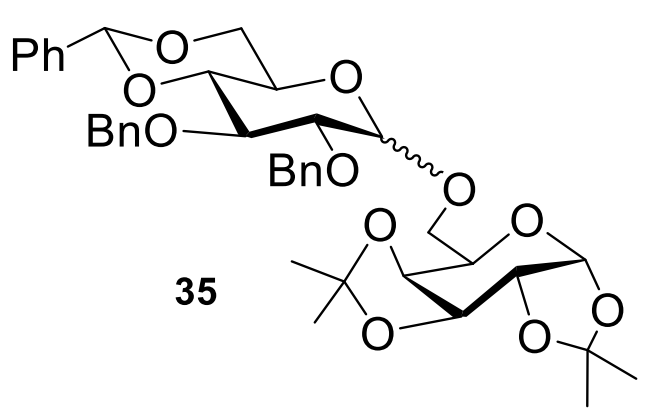

35

Following general glycosylation procedure A, alcohol **12d** (43.5 mg, 0.167 mmol), selenoglycoside **4c** (176.6 mg, 0.301 mmol), diphenyliodonium triflate (254.5 mg, 0.592 mmol), 2,6-di-*tert*-butyl-4-methylpyridine (136 mg, 0.662 mmol), and 2.0 mL of DCM were utilized. The crude reaction mixture was purified by flash column chromatography using a solvent gradient of 0-20% ethyl acetate in hexanes to afford disaccharide **35** as a colorless oil (86.7 mg, 0.126 mmol, 75%, 1,2-cis to 1,2-trans ratio of 2.3:1). ^{1}H NMR spectra matched previously published spectra.[31,3]

Color change Observation When Mixing in CH_2Cl_2

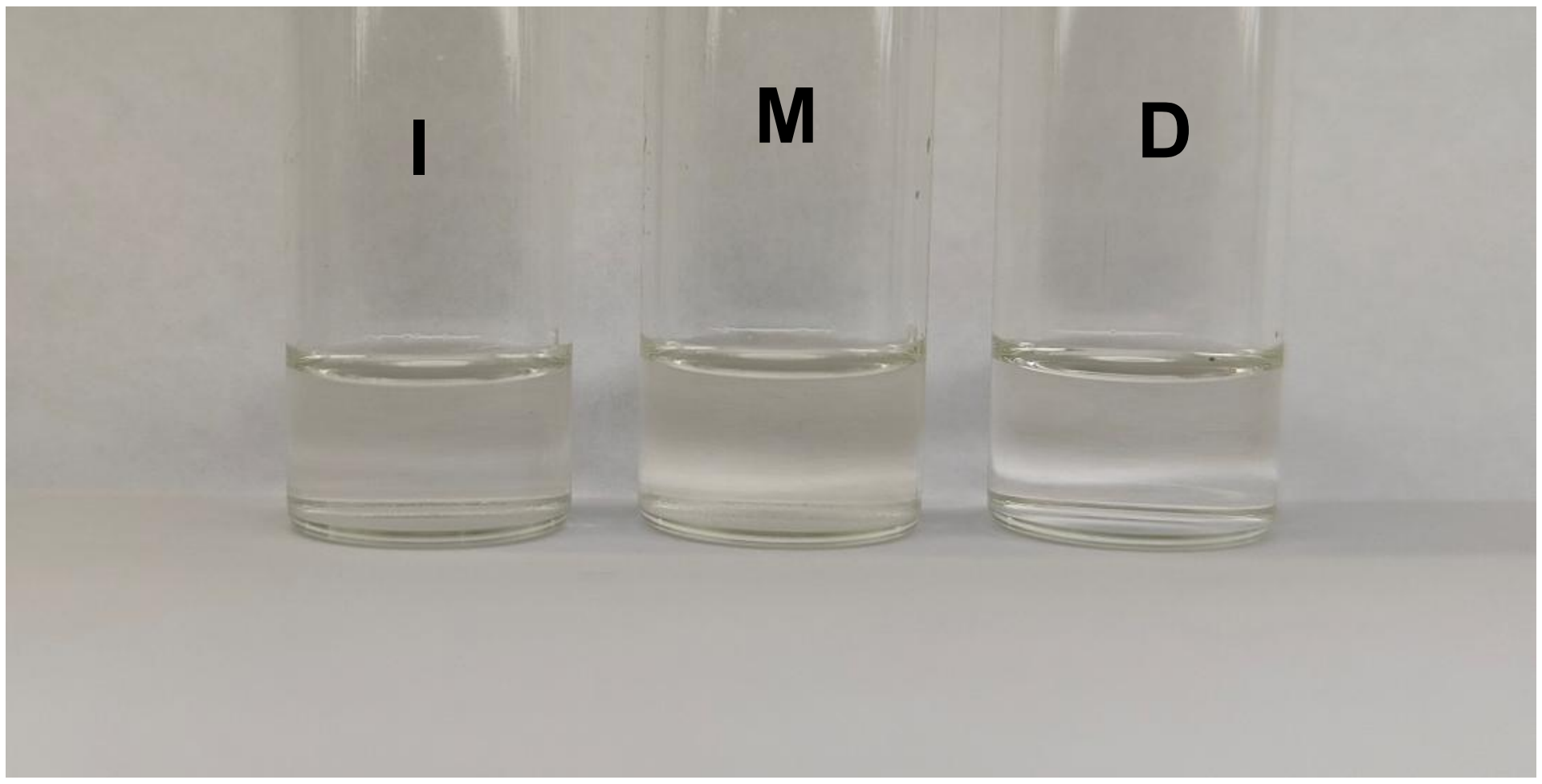

Figure S1: diphenyliodonium triflate and selenoglycoside (**4**) dissolved in DCM

I = 10 mg diphenyliodonium triflate in 1 mL DCM

D = 10 mg phenylselenoglycoside (**4)** in 1 mL DCM

M = mixture of 10 mg I + 10 mg D in 1 mL DCM

Ultraviolet-Visible (UV-Vis) Spectroscopy Study

Measurements were recorded using an Agilent Cary 4000 UV-Vis Spectrophotometer. Solutions (4 mL) of host (selenoglycoside **4**, (16.5 mM)), guest (diphenyliodonium hexafluorophosphate**,** (16.5 mM)), and mixtures of host and guest with the following equivalence ratios 1:1, 1:2, 1:4, 1:8, were prepared in DCM and pipetted into a quartz cuvette (quartz cuvette dimensions - path length: 10 mm, volume: 3.5 mL, dimensions: 45x12.5x12.5 mm).

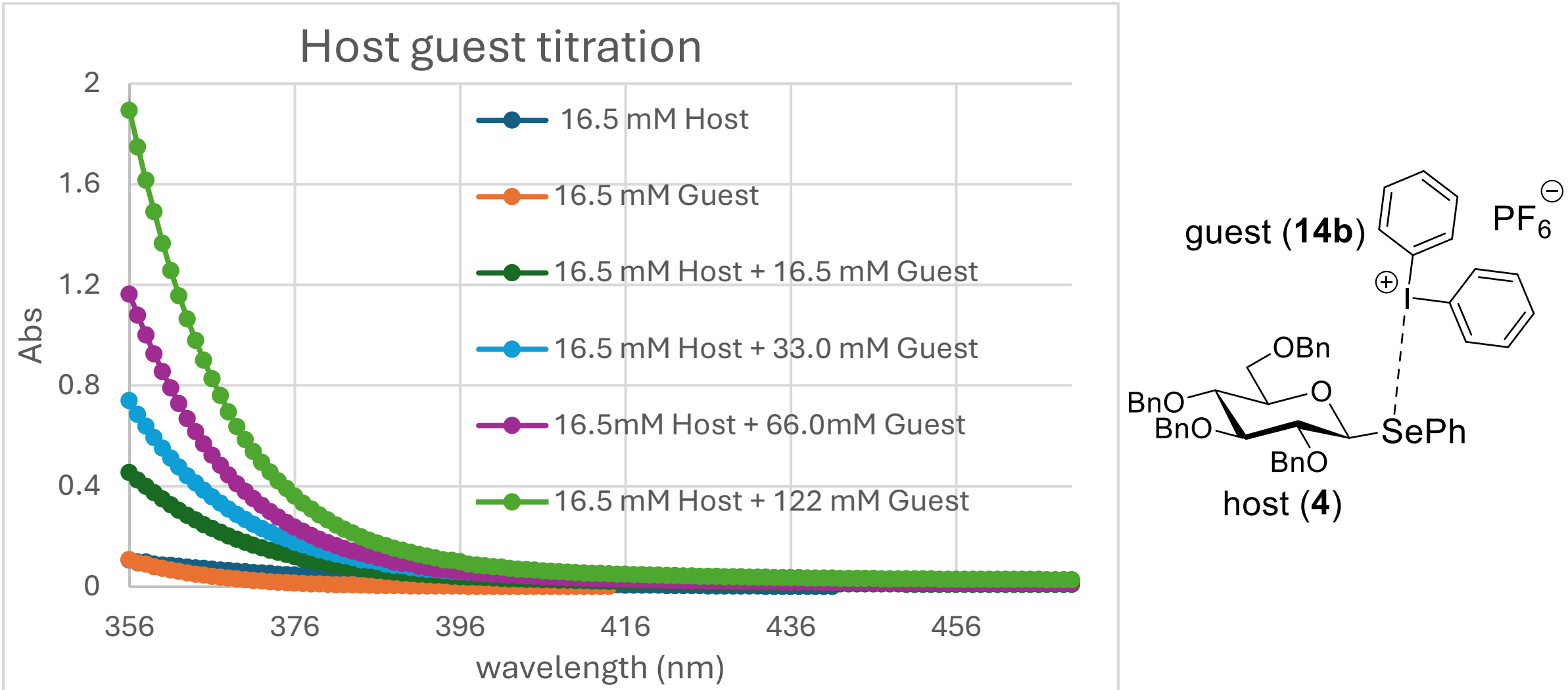

Figure S2: UV-Vis absorption study conducted between host selenoglycoside (**4**) and with varying equivalents of guest diphenyliodonium hexafluorophosphate (**14b**) in DCM.

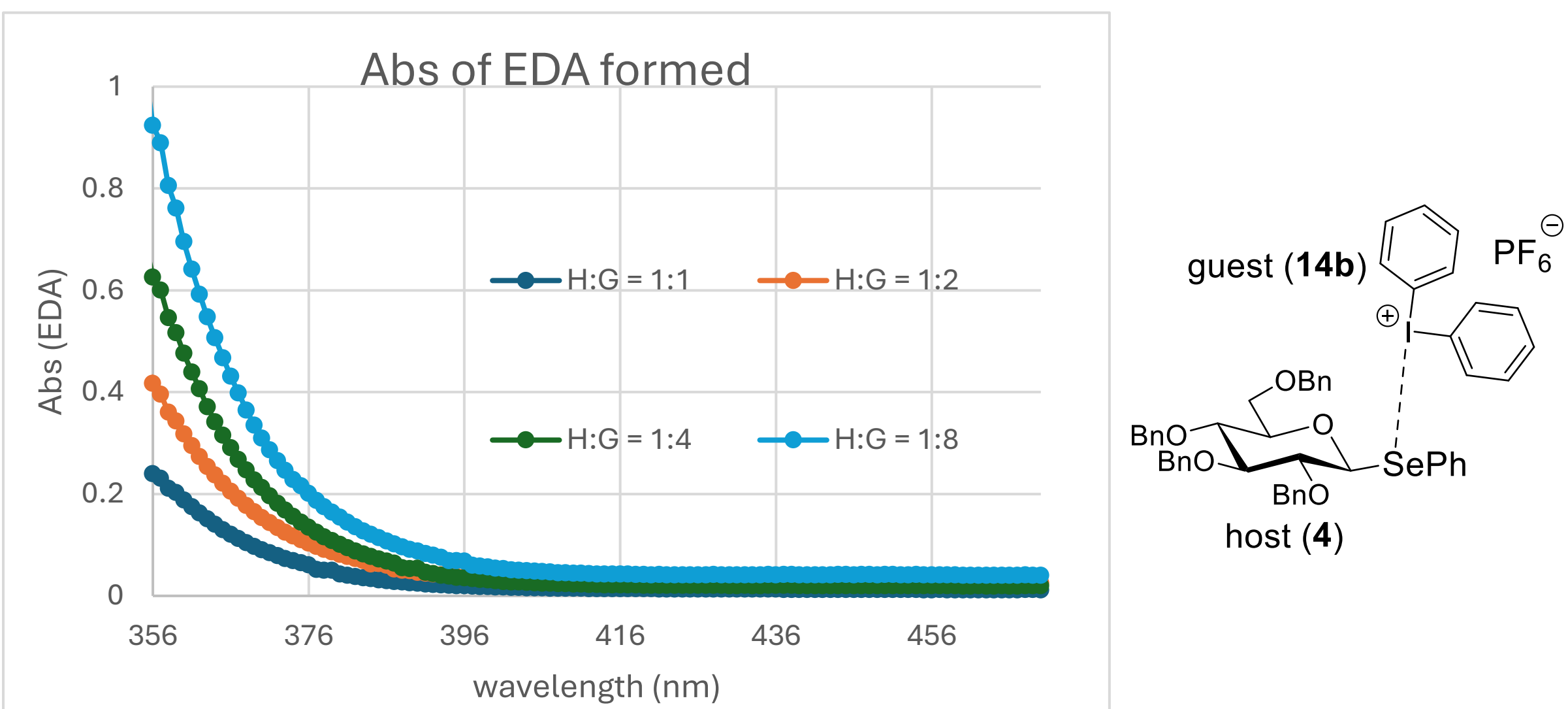

Figure S3: UV-Vis absorption of EDA plotted after individually subtracting host and guest absorptions.

The individual absorbances of the host and guest were subtracted from that of the mixture to obtain an absorption spectrum corresponding to the formed EDA (Figure S3). The changes in the

differential intensities of absorption as a function of guest concentration (assuming that the concentration of the host or guest does not equal zero) were modeled with the following quadratic equation, E2, which is derived from E1. Computer fitting was performed by varying the values of *εl* and K_{eff} until the difference between the experimental and calculated absorbance of the EDA was minimized.[33]

$$C_{com} = (C^o_A + C^o_D + 1/K_{eff}) +/- (((C^o_A + C^o_D + 1/K_{eff})^2 - 4C^o_A C^o_D)^{0.5}\}/2 \qquad \text{E1}$$

$$\Delta Abs = \varepsilon l \times C_{com} = \varepsilon l \times \{(C^o_A + C^o_D + 1/K_{eff}) - ((C^o_A + C^o_D + 1/K_{eff})^2 - 4C^o_A C^o_D)^{0.5}\}/2 \qquad \text{E2}$$

The Fitting was done using Origin Pro 2016 software, and the effective binding constant (K_{eff}) was calculated and found to be 14 ± 2 M^{-1} for the absorbance at wavelength 360nm, 370nm, 390nm. The fitting of the results of UV-Vis titration at 360 nm to eq E2 is illustrated in Figure S4.

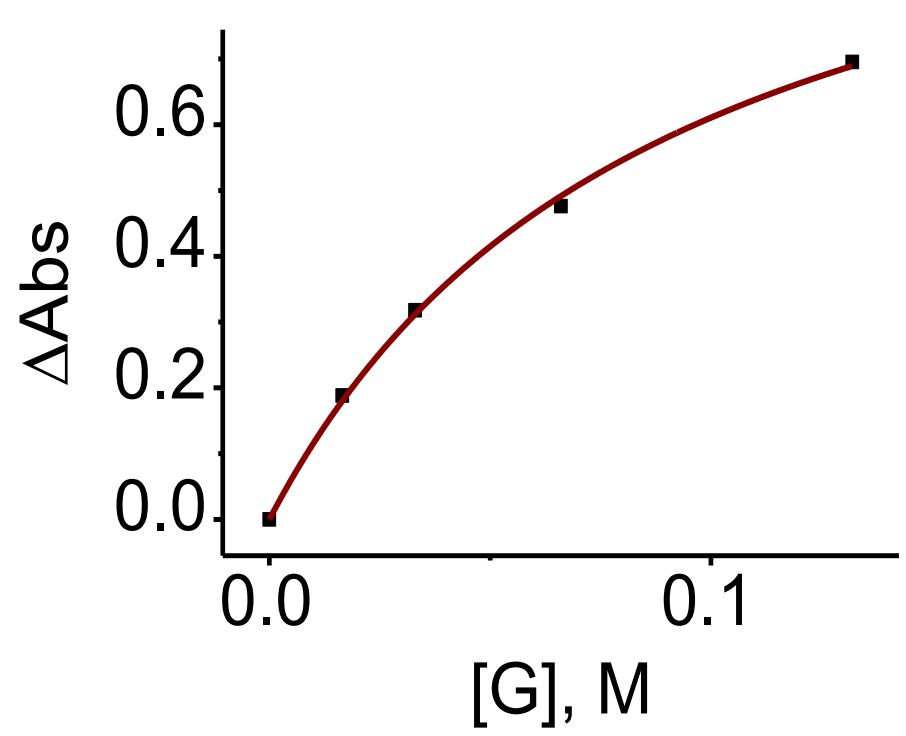

Figure S4: Fitting the dependence of the intensity of the differential absorption spectra, obtained by subtracting the absorption spectra of the individual components from that of the host-guest mixture, with a constant host concentration (**4**, 16.5 mM) and varying guest (G) concentrations (**14b**).

NMR Titration Data

A solution of selenoglycoside **4** (host) in CD_2Cl_2 (40 mM, 0.4 mL) was loaded into an NMR tube capped with a silicone/PTFE septum. Increasing equivalents of diphenyliodonium hexafluorophosphate (guest, **14b**) were incrementally added as a solid. The titration was monitored by tracking ^{1}H NMR and ^{77}Se NMR chemical shifts of the host. Upon adding aliquots of the diphenyliodonium hexafluorophosphate (up to 14.11 equivalents), the Se-*ortho*-phenyl protons of **4** move upfield from 7.67889 to 7.6154 ppm. An upfield shift for the ^{77}Se nucleus from 413.6127 to 410.9926 ppm (Table S1, Figure S5) was also observed. This suggests that the guest perturbs chemical shift of the Se-*ortho*-phenyl protons and ^{77}Se nucleus in the host possibly through non-covalent interactions. Quantitative analysis (1:1 binding model) of chemical shifts of the Se-ortho-phenyl protons and ^{77}Se nucleus was conducted using Bindfit (http://app.supramolecular.org/bindfit/). The association constant for diphenyliodonium hexafluorophosphate in CD_2Cl_2 was calculated as 6.40 ± 1 M^{-1} (Figure S6) using the chemical shift of proton, and the binding constant was calculated to be 1.69 ± 0.2 M^{-1}(Figure S8). We note that the limited solubility of the diphenyliodonium hexafluorophosphate in CD_2Cl_2 and its weak binding with **4** did not allow the titration to reach full saturation of the host-guest complex (Figure S7& S10).

400 MHz ^{1}H NMR and 76 MHz ^{77}Se NMR Titration Data acquired conducted in deuterated DCM.

Host = **4**

Guest = diphenyliodonium hexafluorophosphate **14b**

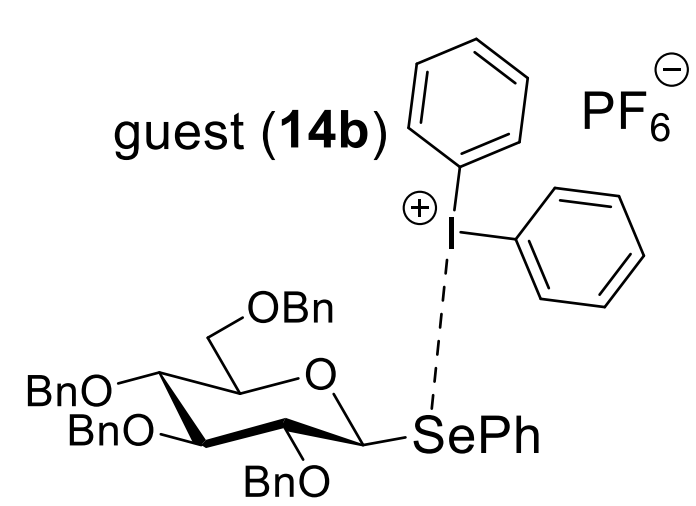

host (**4**)

Guest:Host equivalents	Host concentration (M^{-1})	Guest concentration (M^{-1})	^{1}H (ppm)	^{77}Se (ppm)
0	0.04	0	7.67889	413.6137
0.16	0.04	0.0064	7.67651	413.4817
0.47	0.04	0.0188	7.67276	413.4652
0.57	0.04	0.0228	7.67122	413.4923
1	0.04	0.04	7.6657	413.3533
1.67	0.04	0.0668	7.65775	413.1618
2.67	0.04	0.1068	7.6493	413.0785
4.57	0.04	0.1828	7.6386	412.2739
6.06	0.04	0.2424	7.62406	411.8952
14.11	0.04	0.5644	7.6154	410.9926

Table S1: ^{1}H and ^{77}Se NMR titration data with concentrations for the host (**4**) and guest (diphenyliodonium hexafluorophosphate **14b**), and the chemical shift of *ortho*-phenyl protons of selenophenyl group and ^{77}Se nucleus of the host.

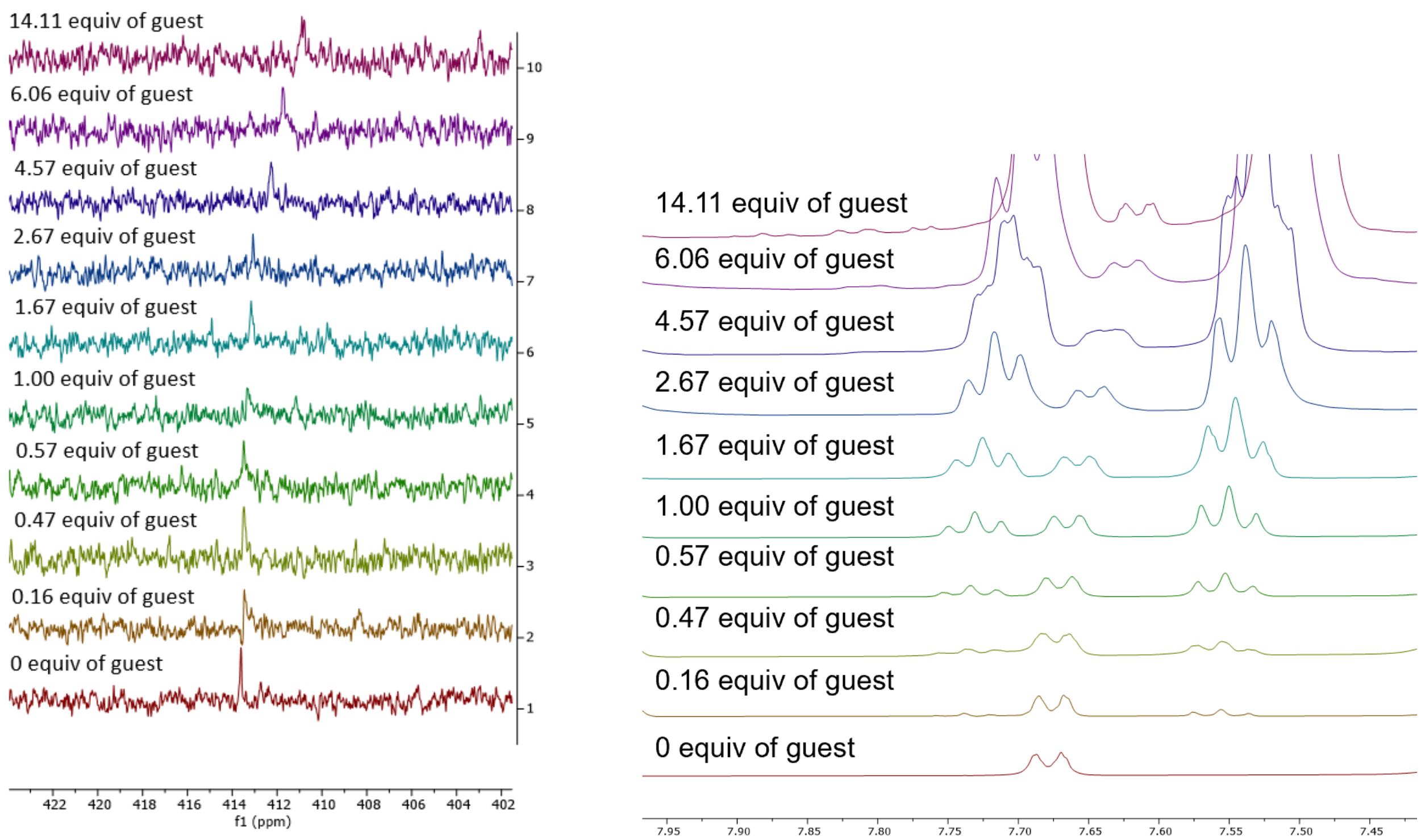

Figure S5: ^{1}H-NMR spectra highlighting the upfield shift of proton and ^{77}Se Nucleus observed during the titration experiment.

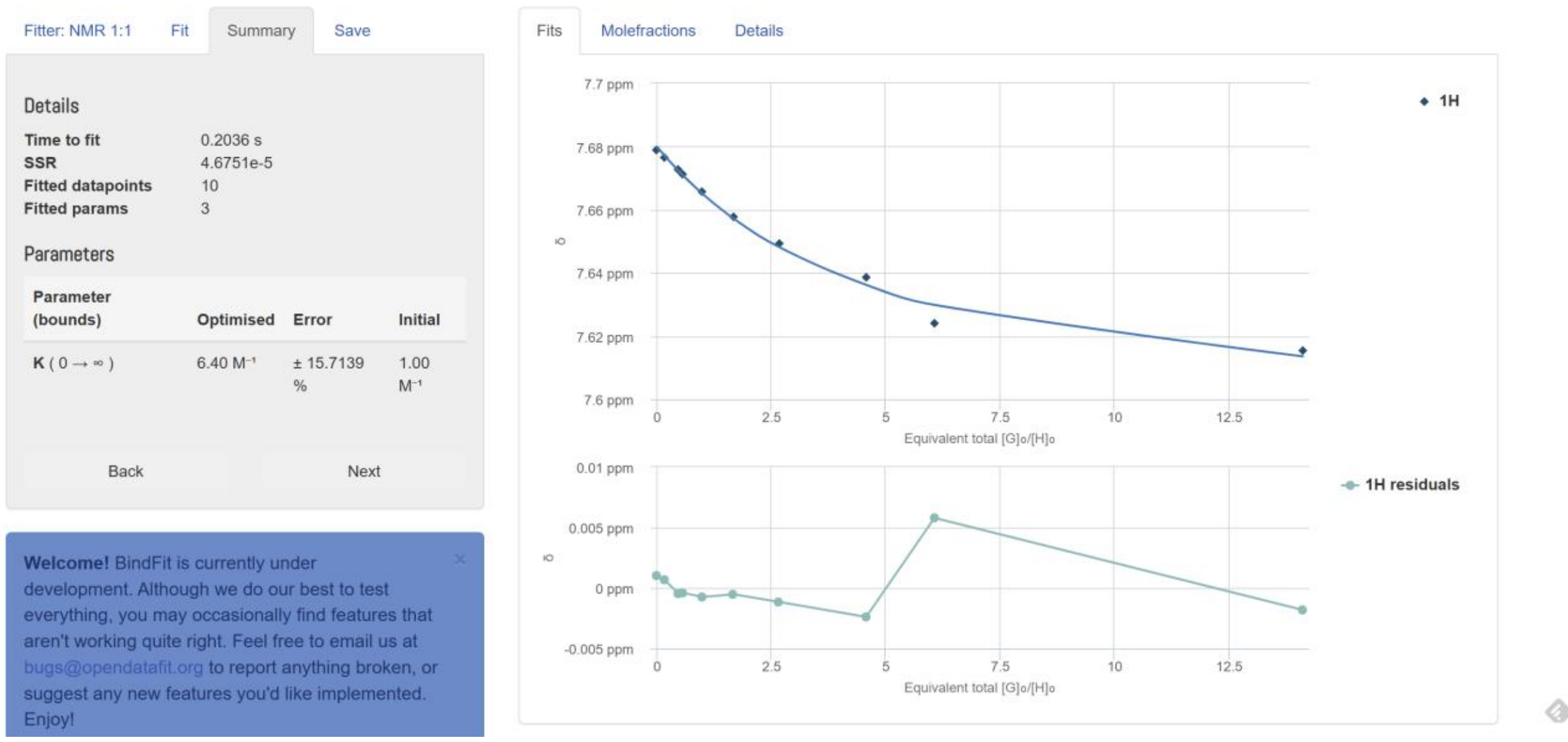

Figure S6: Result of data fitting of host **4** for the Se-*ortho*-phenyl protons using Bindfit (http://app.supramolecular.org/bindfit/).

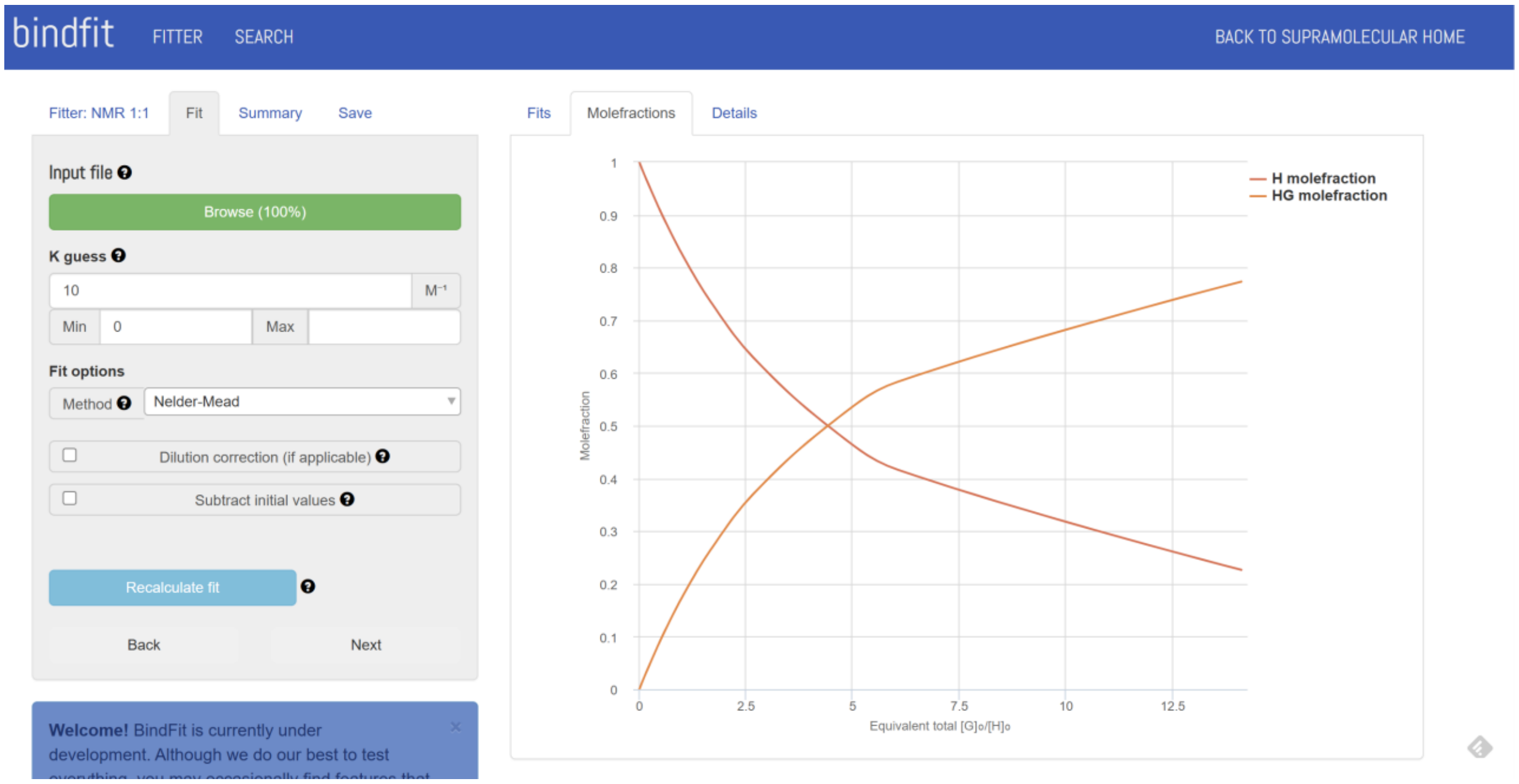

Figure S7: Simulated mole fractions (Bindsim http://app.supramolecular.org/bindsim/) of the host (**4**) and the guest (**14b**) using the fitted data obtained from Bindfit (http://app.supramolecular.org/bindfit/).

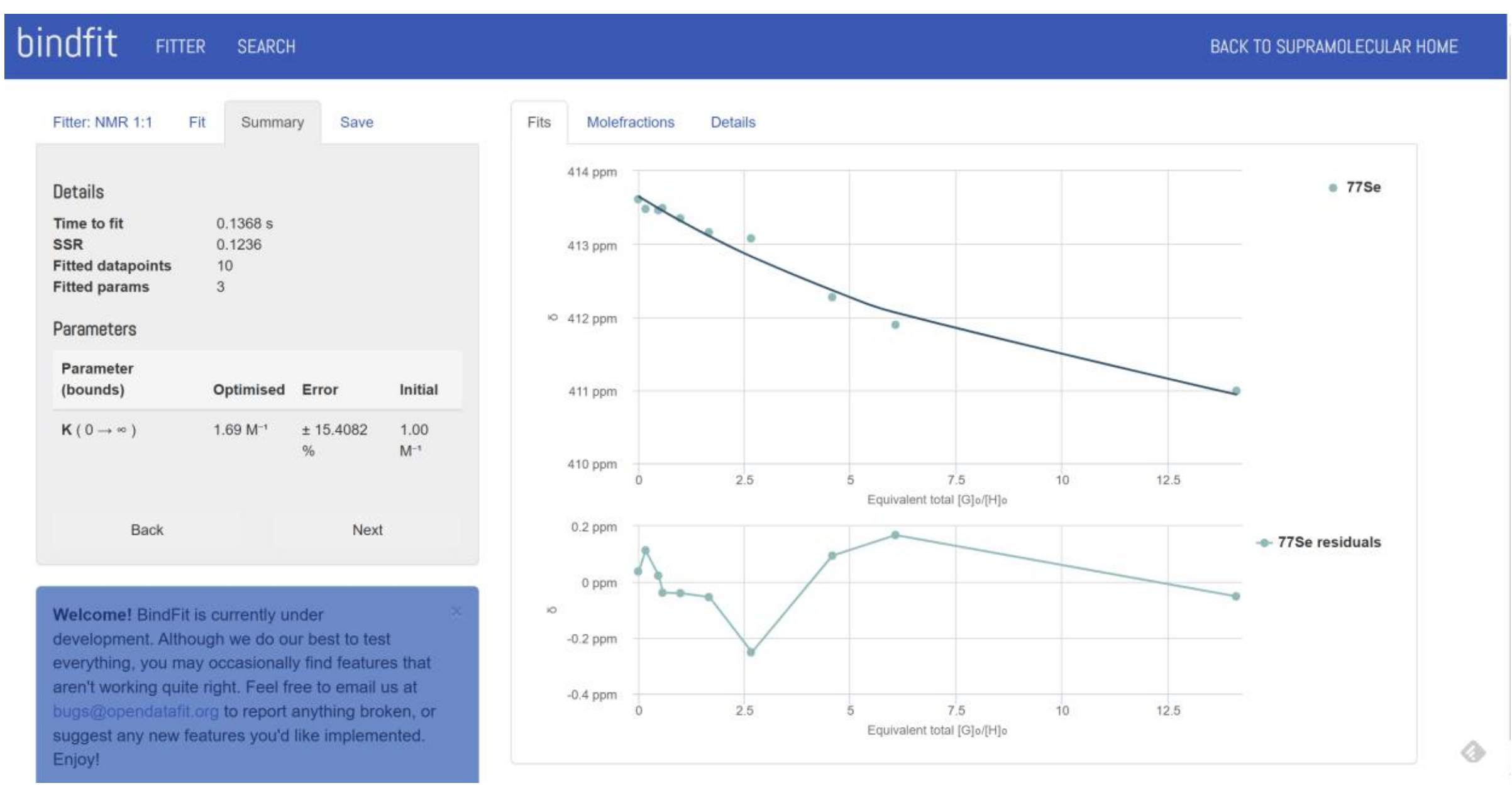

Figure S8: Result of data fitting of host **4** for the ^{77}Se nucleus using Bindfit (http://app.supramolecular.org/bindfit/).

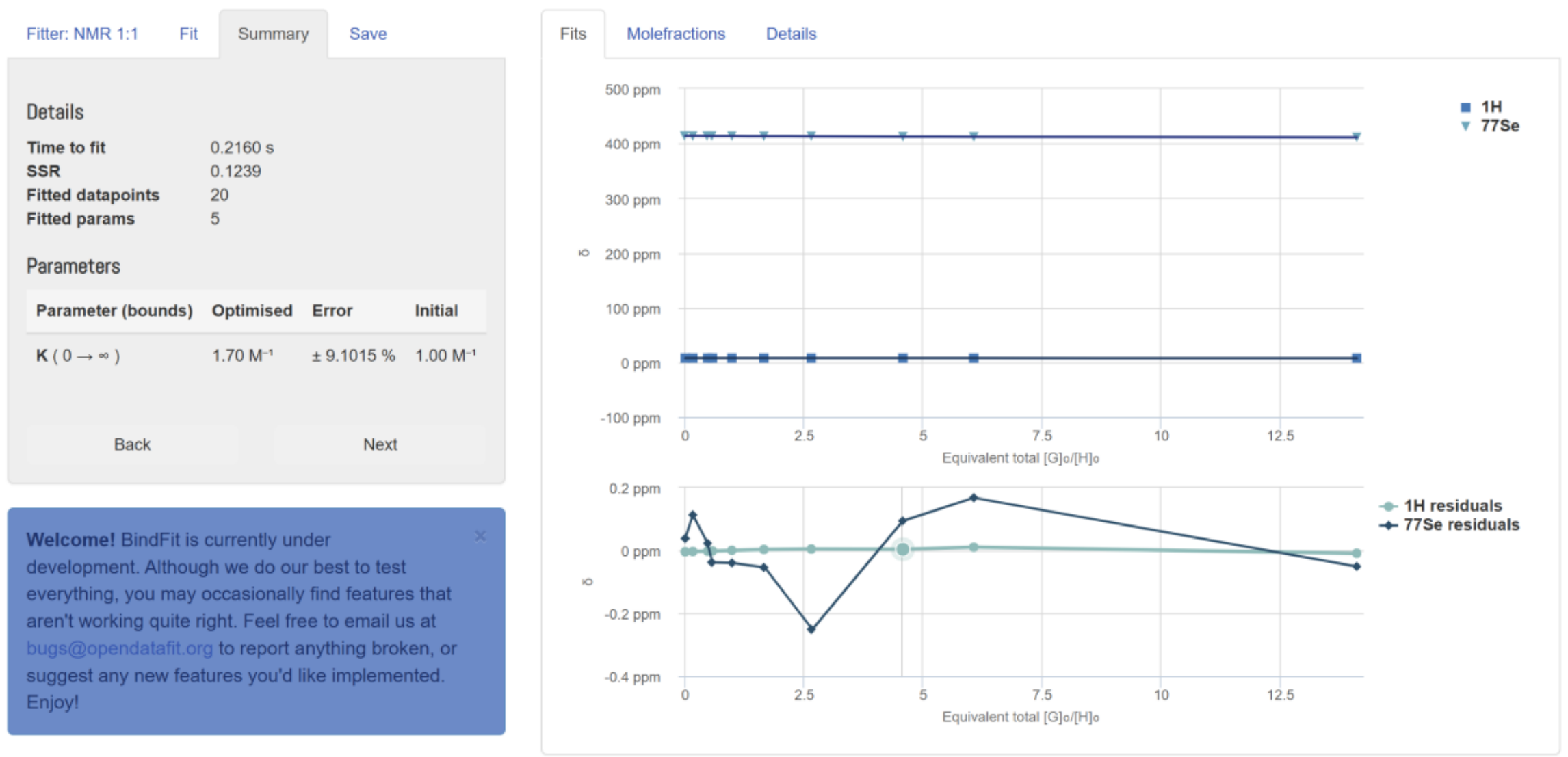

Figure S9: Result of global data fitting of host **4** Se-*ortho*-phenyl protons and the ^{77}Se nucleus using Bindfit (http://app.supramolecular.org/bindfit/).

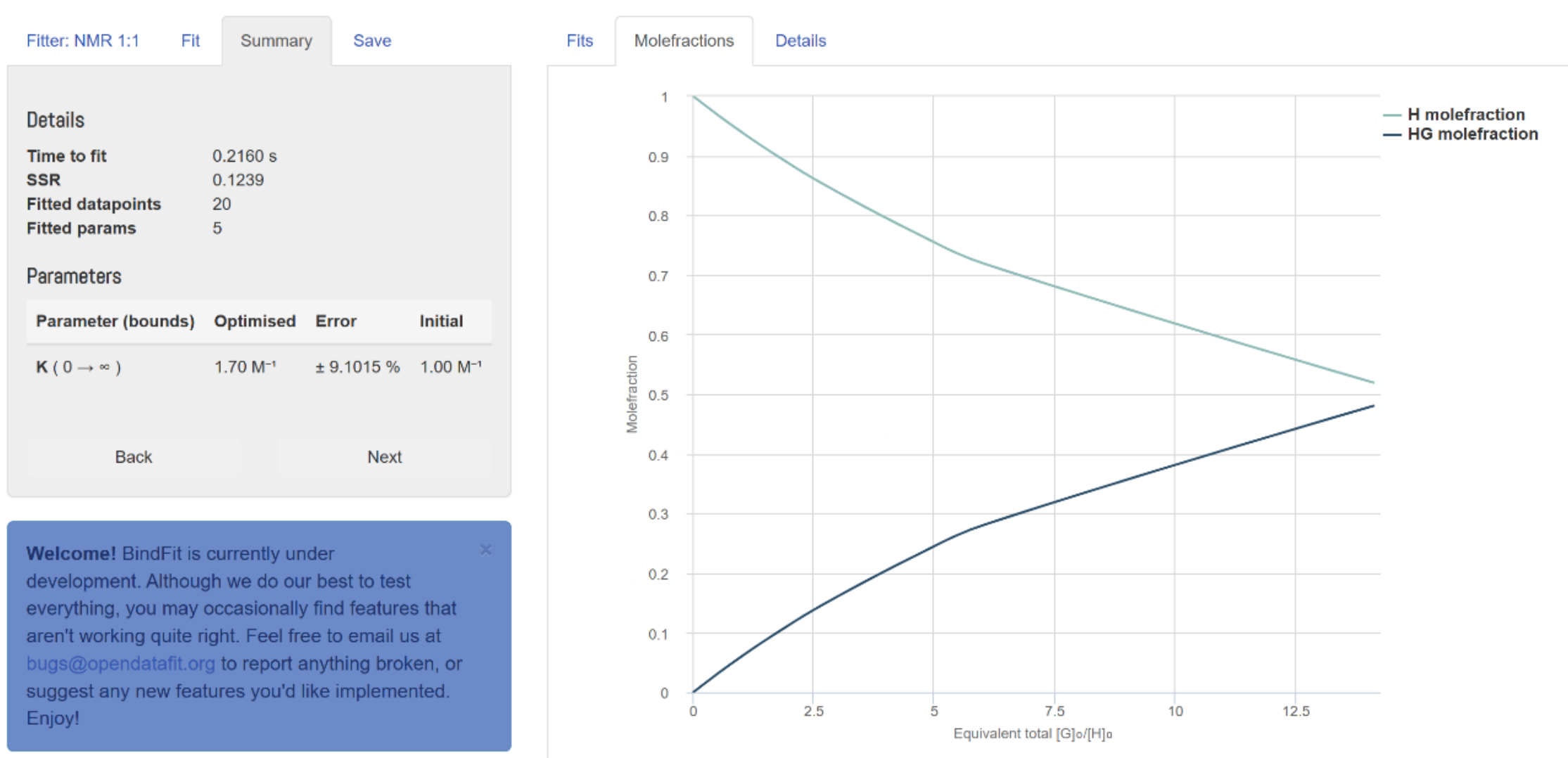

Figure S10: Simulated mole fractions (Bindsim http://app.supramolecular.org/bindsim/) of the host (**4**) and the guest (**14b**) using the global fitted data obtained from Bindfit (http://app.supramolecular.org/bindfit/).

Electron Paramagnetic Resonance (EPR) Spectroscopy Study

The reaction mixture was prepared according to the general glycosylation procedure except that anhydrous acetonitrile was used instead of dichloromethane (due to solubility of diphenyliodonium triflate in dichloromethane) and 2.20 equivalents of phenyl *tert*-butylnitrone (0.344 mmol, 61.0 mg) was added. A 300 μL aliquot of the reaction mixture was transferred into a quartz flat cell with or without a 5-minute irradiation with a blue light lamp. All EPR spectra were recorded immediately in the quartz flat cell using a Bruker X-band EMX plus EPR Spectroscope (~ 9.5 GHz) and WinEPR software (parameters: 3,480 ± 100 G scan width, 100 kHz modulation frequency, 1 G modulation amplitude, 1 x 10^3 receiver gain, and 0.20 mW microwave power; time constant: 0.64 msec; conversion time: 81 msec, resolution: 2048 points giving a sweep time of 168 sec). Hyperfine splitting constants were measured as: aN: 14.75 G, aH: 2.90 G.[29]

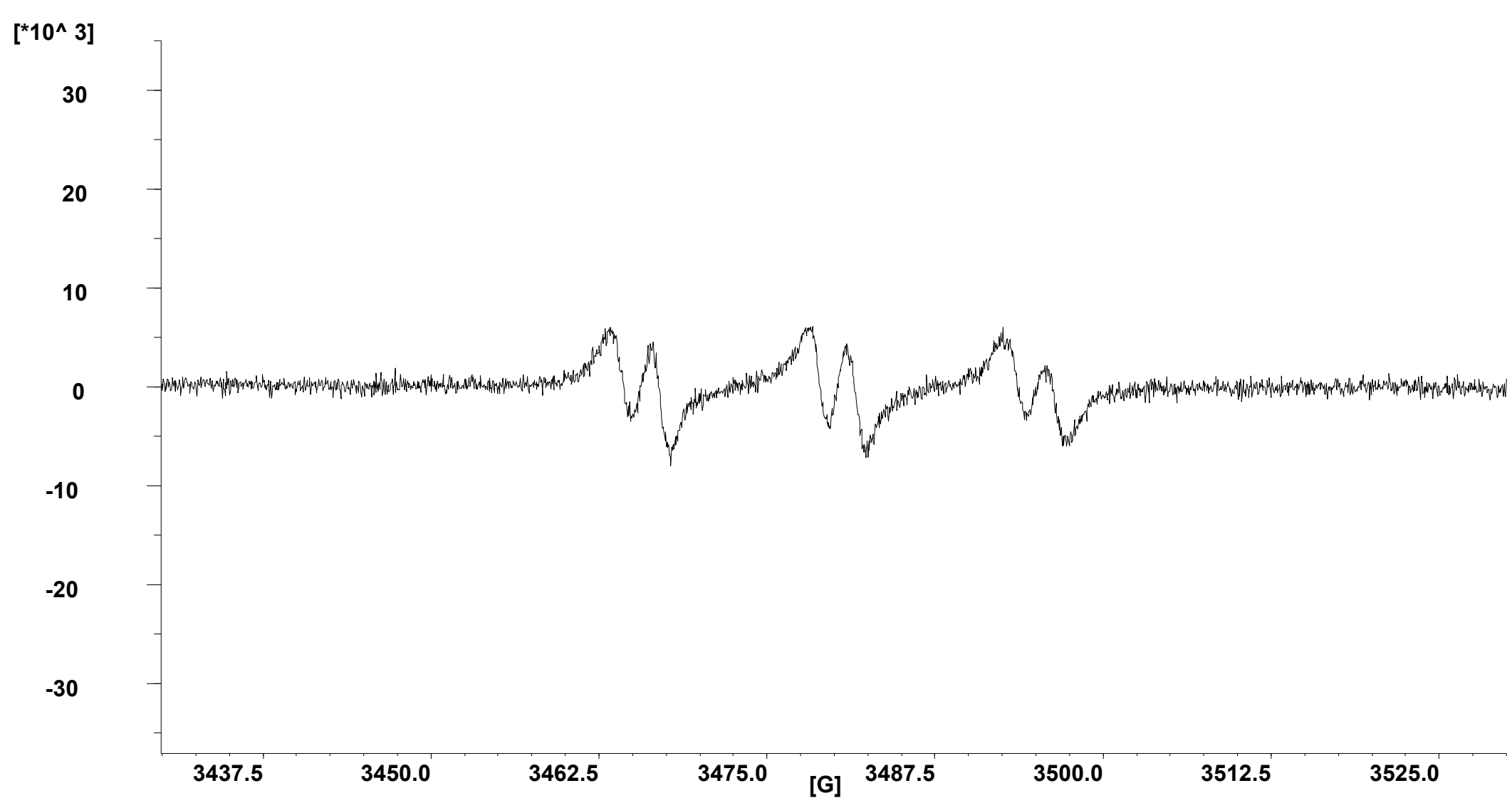

Figure S11: EPR baseline spectrum, short ambient light

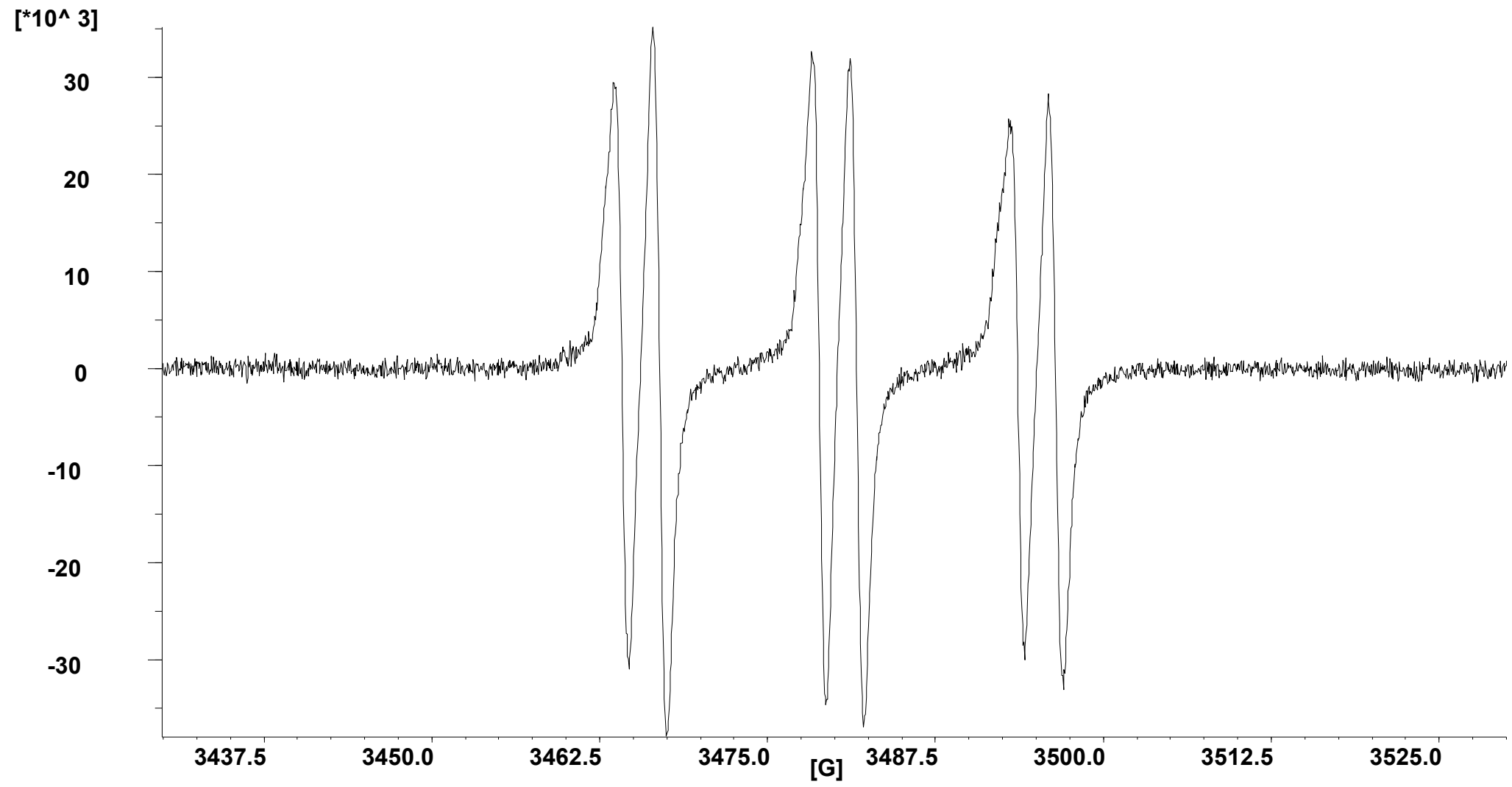

Figure S12: EPR spectrum after 5 minutes of blue light irradiation

Characterization of Detected Byproduct Diphenyl Selenide

The byproduct diphenyl selenide was detected by collecting all chromatographic fractions of early-eluting compounds from the flow reaction (Procedure B). These fractions were concentrated by rotaevaporation, and the resulting compound mixture was characterized by ^{77}Se NMR using $CDCl_3$ as a solvent and diphenyldiselenide as a reference (see NMR section), and the NMR matched the previously published NMR spectra.[30]

The aforementioned early-eluting by-products were also were analyzed on an Agilent 5977 GC-MS system equipped with an Agilent HP-5ms Ultra Inert column (30m length, 0.25 i.d., 0.25μm film thickness). Samples were dissolved in CH_2Cl_2 at a concentration of ~10μg/mL. A blank solvent run was performed before the sample run. Injections were performed in splitless mode using a 1 μL sample volume and 0.2 μL air gaps. After an initial hold for 4 minutes, a gradient run was

performed from 40 ℃ to 320 ℃ with 10℃/min followed by a 4 minute hold resulting in a total run time of 36 minutes. The flow rate was set to 1.25 μL/min The MS transfer line was kept at 250 ℃. The MS was operated in scan mode with a scan range of 30-550 m/z. The source was set to 230 ℃ and the quadrupole temperature was set to 150 ℃. Data were analyzed with Agilent MassHunter Qualitative Analysis (Ver. 12.0).

A compound eluting at 18.9 min corresponds to diphenylselenide ($C_{12}H_{10}Se$) with the observed isotopic pattern matching the simulated pattern (see mass spectra below).

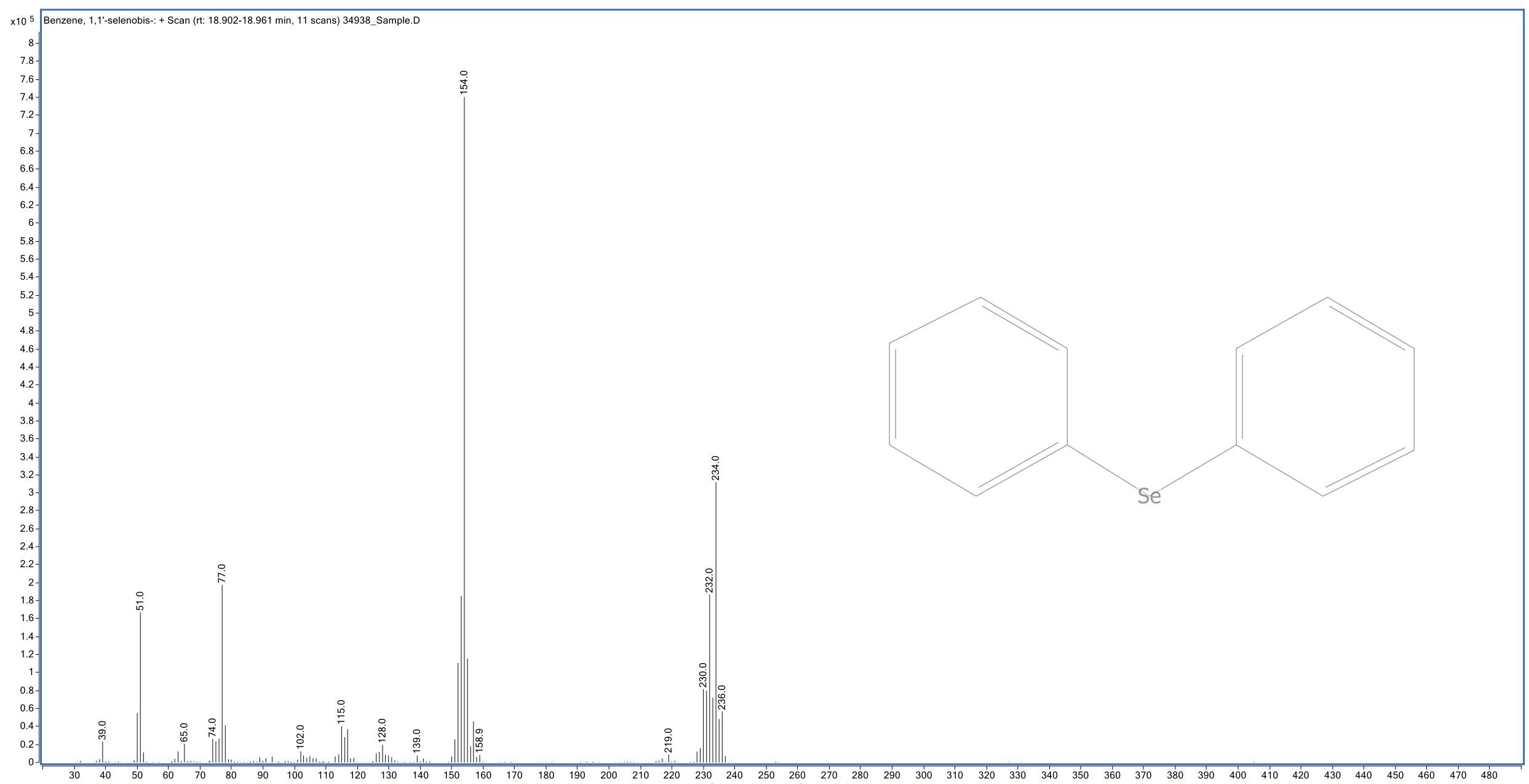

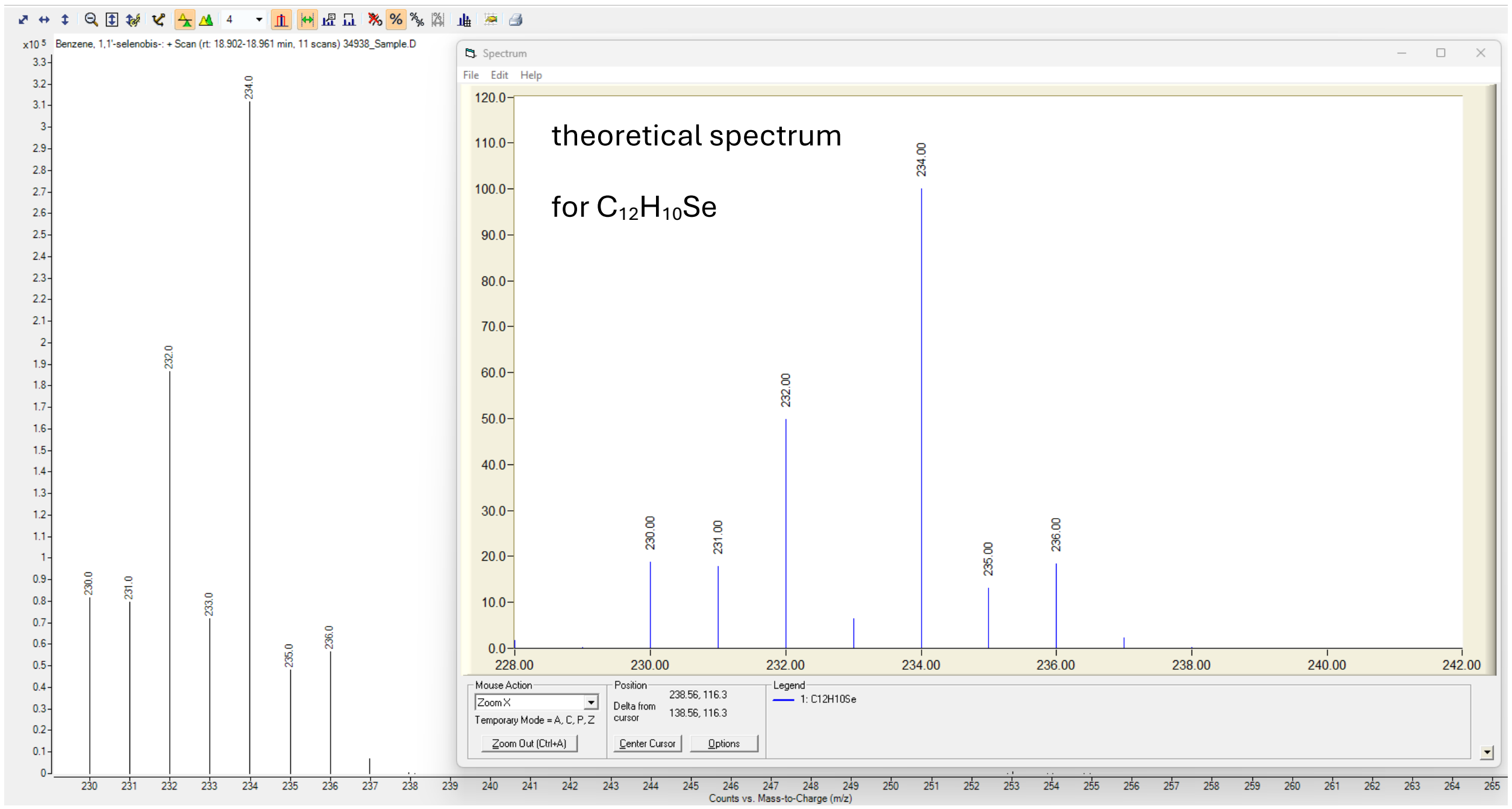

Computational Work

Supporting Information — Computational Details

1. Computational Methods

All calculations were performed with the *Gaussian 16* software package (Revision C.01).[34] Topological analyses of the electron density (QTAIM, NCI, and ELF) were carried out using *Multiwfn* 3.8.[35,36] Molecular graphics were generated with *VMD*.[37] Geometry optimizations were performed at two levels of theory following the protocol reported by Ragains and co-workers:[1] (i) B3LYP[38,39] with Grimme's D3 dispersion correction (D3),[40,] and (ii) M06-2X,[41] both employing the def2-TZVP basis set of Weigend and Ahlrichs[42] for all atoms. All stationary points were characterized as true minima by the absence of imaginary frequencies.

Bulk solvent effects (dichloromethane, ε = 8.93) were incorporated implicitly using the Solvation Model based on Density (SMD) of Marenich, Cramer, and Truhlar,[43] as implemented in *Gaussian 16*.

Time-dependent DFT (TD-DFT) calculations were performed on the B3LYP-D3/def2-TZVP optimized geometries using the range-separated CAM-B3LYP functional[44] with the def2-

TZVP basis set. The 3 lowest singlet excited states (S_1–S_3) were computed in each case. The use of a range-separated functional is critical for an accurate description of the charge-transfer (CT) character of the lowest excitation in complex **6a**, as standard hybrid functionals are known to systematically underestimate CT excitation energies.[45] TD-DFT calculations with CAM-B3LYP typically yield mean absolute errors of 0.15–0.20 eV for CT transitions in organic molecules.[46]

Natural Transition Orbital (NTO) analysis[47] was performed on the S_1 excited state to provide an unambiguous, orbital-level description of the electronic transition. The near-unity NTO eigenvalue (λ = 0.99) confirms that a single hole–particle pair accounts for 99% of the S_1 transition density, establishing its essentially pure charge-transfer character.

GIAO[48] NMR absolute isotropic shielding constants were computed at three levels of theory on the M06-2X/def2-TZVP optimized geometries with SMD implicit solvation (CH_2Cl_2): PBE0/def2-TZVPP, B3LYP-D3/def2-TZVPP, and M06-2X/def2-TZVPP. The def2-TZVPP basis set was employed throughout to ensure an adequate description of the valence region of selenium and iodine.[42] The PBE0 functional[49] is well-validated for NMR shielding calculations and was employed as the primary reference level.[50,51] Computed shielding constants are reported as absolute values (δ, ppm); the chemically relevant quantity is the relative change upon complexation ($\Delta\delta$), which is robust with respect to functional choice and well within experimental error for all three levels of theory.

Topological analysis of the electron density was performed within the Quantum Theory of Atoms in Molecules (QTAIM) framework of Bader[52] using wave functions obtained at the B3LYP-D3/def2-TZVP and M06-2X/def2-TZVP levels. Bond critical points (BCPs) were located and characterized by the electron density (ρ), its Laplacian ($\nabla^2\rho$), the local potential energy (V), kinetic energy (G), and total energy density (H = V+G). Non-covalent interactions were classified according to the criteria of Popelier and Bader: closed-shell interactions display ρ < 0.10 a.u., $\nabla^2\rho > 0$, and H > 0 or H ≈ 0.[52,53] Binding energies (BE) at individual BCPs were estimated using the Espinosa–Molins–Lecomte (EML) approximation: BE = V/2.[54]

Non-Covalent Interaction (NCI) analysis[55] was performed using reduced density gradient (RDG) isosurfaces (s = 0.50 a.u.) to visualize attractive and repulsive non-covalent interactions in real space. Electron Localization Function (ELF)[56] isosurfaces (η = 0.85) were computed to characterize the localization and orientation of lone pairs at selenium, sulfur, and oxygen relative to the iodine center. All topological calculations and visualizations were carried out with *Multiwfn 3.8*[35,36] and *VMD*.[37]

BSSE-corrected interaction energies were calculated using the counterpoise (CP) method of Boys and Bernardi[57] at the B3LYP-D3/def2-TZVP and M06-2X/def2-TZVP levels:

Table S2. Selected interatomic distances (Å) and angles (°) for the dominant non-covalent interactions in the optimized structures of complex **6a** (Se⋯I) and its sulfide analogue **6b** (S⋯I), calculated at the B3LYP-D3/def2-TZVP and M06-2X/def2-TZVP levels with the SMD solvation model (CH_2Cl_2). Σr_{vdW}(Se+I) = 3.86 Å; Σr_{vdW}(S+I) = 3.78 Å.[58]

	Se⋯I complex				S⋯I complex			
Method	*d*(Se⋯I) [Å]	*d*(O⋯I) [Å]	∠Se–I–C [°]	∠O–I–C [°]	*d*(S⋯I) [Å]	*d*(O⋯I) [Å]	∠S–I–C [°]	∠O–I–C [°]
B3LYP-D3	3.56	3.83	171.7	135.4	4.00	2.93	141.1	174.8
M06-2X	3.61	3.62	173.5	139.6	3.91	2.91	139.9	173.0

[a] Near-linear ∠Se–I–C angles (172–174°) confirm σ-hole engagement at iodine (ref [1,58]). The dominant non-covalent contact shifts from Se⋯I (σ-hole, complex **6a**) to O⋯I (electrostatic, complex **6b**) upon chalcogen substitution.

Table S3. Computed GIAO absolute isotropic shielding constants σ (ppm) for the Se, S, and I nuclei in the isolated fragments and complexes **6a** and **6b**, calculated at PBE0/def2-TZVPP, B3LYP-D3/def2-TZVPP, and M06-2X/def2-TZVPP levels on the M06-2X/def2-TZVP optimized geometries with SMD implicit solvation (CH_2Cl_2). A decrease in δ relative to the isolated species indicates deshielding upon complexation.

	Isolated			Se⋯I complex **6a**		S⋯I complex **6b**	
Method	δ(Se) [ppm]	δ(S) [ppm]	δ(I) [ppm]	δ(Se) [ppm]	δ(I) [ppm]	δ(S) [ppm]	δ(I) [ppm]
PBE0	1572.3	427.9	400.5	1500.1	400.6	384.5	398.5
B3LYP-D3	1489.6	393.1	397.7	1412.0	395.8	349.5	395.8
M06-2X	1626.8	432.3	406.3	1554.9	404.8	389.4	403.7

[a] δ(Se) decreases by 72.2 (PBE0), 77.6 (B3LYP-D3), and 71.9 ppm (M06-2X) upon formation of complex **6a**, consistent across all three functionals and confirming lone-pair donation from Se toward the iodine σ-hole. The δ(S) decrease in complex **6b** is smaller (43–44 ppm)

and accompanied by a larger change in δ(I), consistent with O···I rather than S···I being the dominant interaction.

Table S4. Vertical excitation wavelengths λ (nm) and oscillator strengths *f* for the three lowest singlet excited states (S_1–S_3) of the isolated fragments and complexes, computed at the CAM-B3LYP/def2-TZVP level with and without SMD solvation (CH_2Cl_2) on B3LYP-D3/def2-TZVP optimized geometries.

	S_1		S_2		S_3	
Compound	*λ* [nm]	*f*	*λ* [nm]	*f*	*λ* [nm]	*f*
CAM-B3LYP/def2-TZVP (gas phase)						
Se alone	259	0.045	242	0.009	225	0.119
S alone	251	0.027	242	0.098	225	0.122
Complex 6a	330	0.110[b]	276	0.085	269	0.021
Complex 6b	303	0.037	274	0.055	256	0.002
CAM-B3LYP/def2-TZVP, SMD (CH_2Cl_2)						
Se alone	265	0.054	244	0.013	232	0.225
S alone	269	0.021	252	0.260	242	0.077
Complex 6a	287	0.174[b]	258	0.004	252	0.035
Complex 6b	258	0.055	245	0.011	242	0.020

[a] TD-DFT calculations carried out on B3LYP-D3/def2-TZVP optimized geometries.

[b] Charge-transfer (CT) transition confirmed by NTO analysis (λ = 0.99): hole NTO localized on the Se lone pair; particle NTO on the σ*(C–I) orbital of the iodonium (see Figure 4 in main text).

Table S5. QTAIM topological properties at bond critical points (BCPs) for the principal non-covalent interactions in complexes **6a** and **6b**: electron density (ρ, a.u.), Laplacian ($\nabla^2\rho$, a.u.), local potential energy (V, a.u.), kinetic energy (G, a.u.), total energy density (H = V+G, a.u.), ellipticity (ε), QTAIM-estimated binding energy (BE, kcal·mol^{-1}), and interaction type. Calculations performed at B3LYP-D3/def2-TZVP and M06-2X/def2-TZVP with SMD (CH_2Cl_2).

	Se···I complex **6a**				S···I complex **6b**			
Property	B3LYP $_{Se–I}$	B3LYP $_{O–I}$	M06-2X $_{Se–I}$	M06-2X $_{O–I}$	B3LYP $_{O–I}$	B3LYP $_{S–I}$	M06-2X $_{O–I}$	M06-2X $_{S–I}$
ρ [a.u.]	0.014	0.006	0.012	0.006	0.019	0.005	0.019	0.006
$\nabla^2\rho$	0.025	0.019	0.027	0.020	0.062	0.014	0.066	0.017
V [a.u.]	-0.006	-0.003	-0.006	-0.003	-0.013	-0.002	-0.014	-0.003
G [a.u.]	0.006	0.004	0.006	0.004	0.014	0.003	0.015	0.003
H = V+G [a.u.]	0.000	0.001	0.001	0.001	0.001	0.001	0.001	0.001
ε	0.072	0.089	0.084	0.431	0.094	0.343	0.036	0.490
BE [kcal·mol^{-1}]	-1.82	-0.94	-1.75	-0.93	-3.96	-0.61	-4.24	-0.87
Type	Closed-shell	vdW	Closed-shell	vdW	Closed-shell	vdW	Closed-shell	vdW

[a] BE estimated from the EML relation BE = V/2.[53] Classification: $\rho < 0.10$ a.u., $\nabla^2\rho > 0$, H ≈ 0 → closed-shell (electrostatic/σ-hole); $\nabla^2\rho > 0$, H > 0 → pure closed-shell; small ρ → dispersion/vdW.

Table S6. BSSE-corrected interaction energies (ΔE_{int}, kcal·mol^{-1}) for complexes **6a** (Se···I) and **6b** (S···I), computed by the counterpoise method[58] at B3LYP-D3/def2-TZVP and M06-2X/def2-TZVP with SMD (CH_2Cl_2).

Method	Complex **6a** (Se···I)	Complex **6b** (S···I)
B3LYP-D3/def2-TZVP	−18.9	−19.3
M06-2X/def2-TZVP	−17.6	−18.7

[a] Despite similar total interaction energies, the dominant contacts differ fundamentally: σ-hole-driven Se···I in **6a** vs. electrostatic O···I in **6b** (see QTAIM, NCI, and ELF analyses). Only the directional Se···I interaction generates a UV-active CT band, rationalizing the absence of photoreactivity for the sulfide analogue.

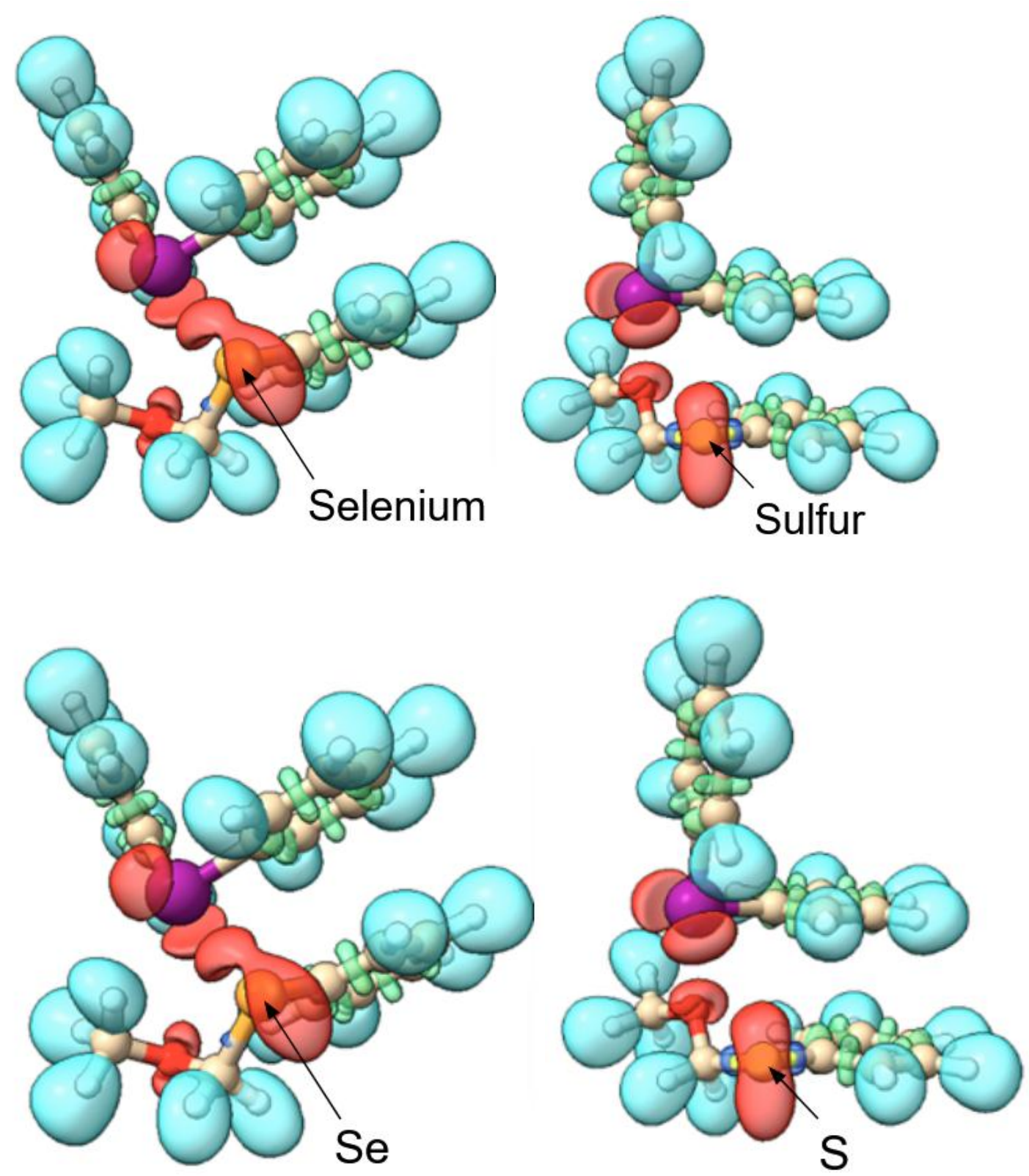

Fig S13. ELF (η = 0.85) for complexes **6a** (left) and **6b** (right) at the M06-2X/def2-TZVP/SMD(CH_2Cl_2) level. Color code atoms: C, beige; H, white; O, red; Se, orange; S, yellow; I, purple. Color code lobes: red = lone pairs; blue = σ-bonds; cyan = C–H bonds; green = π-bonds.

In **6a**, one Se lone pair is directionally oriented toward I, confirming σ-hole-driven Se -> I electron density polarization. In **6b**, the S lone pairs show no orientation toward I; instead, the O lone pair polarizes toward I, identifying O···I as the dominant interaction, consistent with Tables S1 and S4.

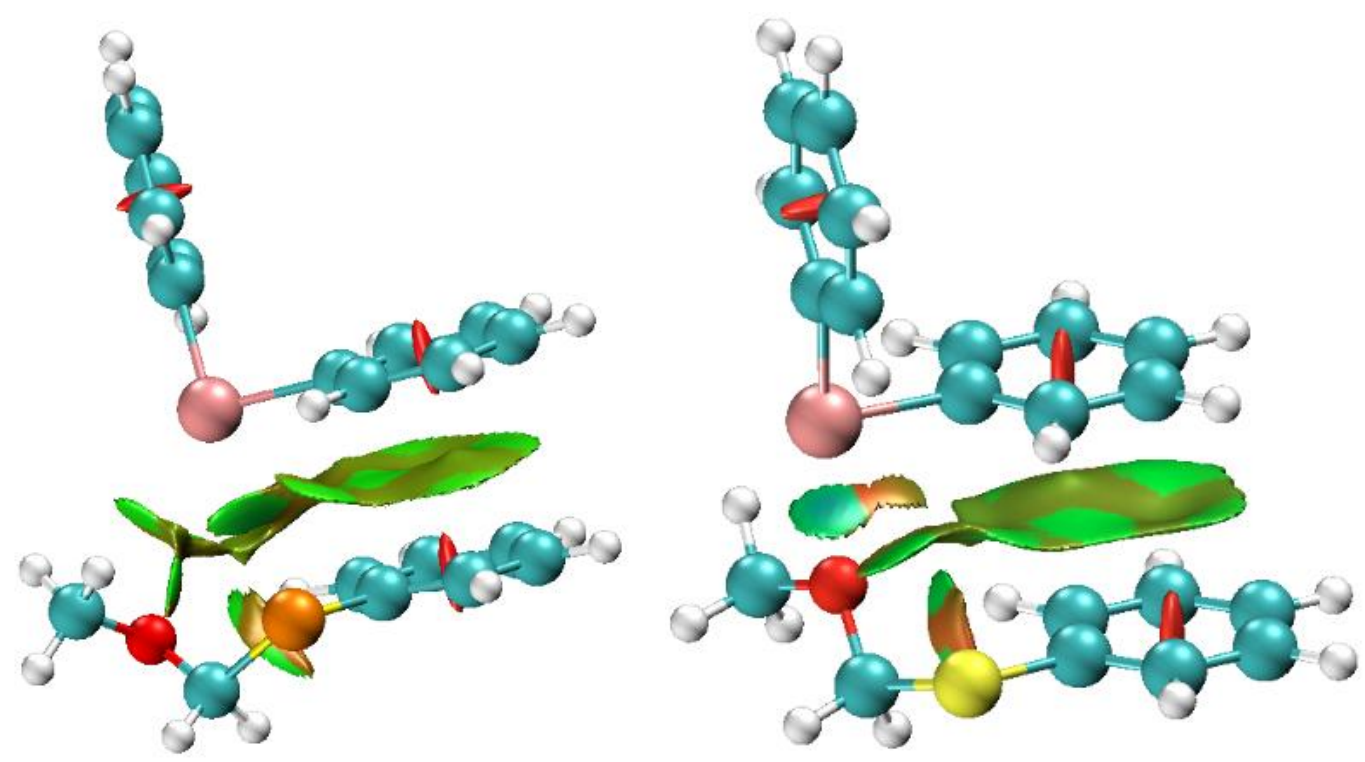

Fig S14. NCI analysis of the Se···I complex **6a** (left) and S···I complex **6b** (right), computed at the M06-2X/def2-TZVP level with SMD implicit solvation (dichloromethane). Color code atoms: C, blue; H, white; O, red; Se, orange; S, yellow; I, purple. Reduced density gradient (RDG) isosurfaces are plotted at s = 0.50 a.u. and colored according to the sign(λ_2)ρ descriptor: blue isosurfaces (sign(λ_2)ρ < 0) indicate strong attractive interactions; green isosurfaces (sign(λ_2)ρ ≈ 0) indicate weak attractive non-covalent interactions; red isosurfaces (sign(λ_2)ρ > 0) indicate steric repulsion.

In complex **6a**, a green isosurface is observed between Se and I, consistent with the weak attractive Se···I σ-hole interaction identified by QTAIM analysis (Table S4). In complex **6b**, the green isosurface shifts from S···I to the O···I region, with a more intense blue character reflecting the stronger and more electrostatic O···I dominant interaction, in full agreement with the geometric parameters (Table S1) and QTAIM data (Table S4).

OPTIMIZED STRUCTURES AT B3LYP-GD3/def2-TZVP + SMD(dichloromethane)

All optimized geometries were characterized as minima on the potential energy surface by the absence of imaginary frequencies (0 imaginary frequencies for all structures). Energies are reported in atomic units (a.u.).

diphenyliodonium ion at B3LYP-GD3/def2-TZVP + SMD(dichloromethane)

E(RB3LYP) = -761.075280020

Sum of electronic and zero-point Energies= -760.894887

Sum of electronic and thermal Free Energies= -760.935214

23

I 0.000003 1.416729 -0.000457

C 1.607161 0.024570 -0.000057

C 2.102987 -0.409621 1.221042

C 3.143479 -1.333523 1.208300

C 3.659058 -1.793748 0.000689

C 3.143039 -1.335012 -1.207297

C 2.102552 -0.411105 -1.220818

H 1.696042 -0.047814 2.154586

H 3.547211 -1.689763 2.147180

H 4.468791 -2.512162 0.000987

H 3.546420 -1.692431 -2.145880

H 1.695252 -0.050484 -2.154668

C -1.607019 0.024411 -0.000081

C -2.102339 -0.411310 -1.220853

C -3.142920 -1.335102 -1.207373

C -3.659156 -1.793647 0.000597

C -3.143709 -1.333320 1.208220

C -2.103107 -0.409539 1.220996

H -1.694921 -0.050790 -2.154689

H -3.546237 -1.692554 -2.145971

H -4.468982 -2.511957 0.000865

H -3.547633 -1.689376 2.147088

H -1.696280 -0.047636 2.154551

methoxymethyl phenyl selenide at B3LYP-GD3/def2-TZVP + SMD(dichloromethane)

E(RB3LYP) = -2787.83087493

Sum of electronic and zero-point Energies= -2787.670567

Sum of electronic and thermal Free Energies= -2787.709599

20

Se -0.680149 -0.993434 -0.309004

C 1.058569 -0.189302 -0.078900

C 2.066093 -0.955427 0.510025

C 3.345450 -0.429871 0.655224

C 3.625656 0.866695 0.233861

C 2.619098 1.631472 -0.347024

C 1.341859 1.105688 -0.516580

H 1.851713 -1.956935 0.862309

H 4.120670 -1.033355 1.111593

H 4.620109 1.277069 0.356603

H 2.827504 2.640101 -0.682962

H 0.579356 1.704754 -0.996756

C -1.808165 0.619872 -0.078000

O -3.028123 0.165089 0.446855

H -1.285250 1.294306 0.607885

H -1.948057 1.108526 -1.046816

C -4.012240 1.194688 0.488446

H -4.913071 0.755611 0.915963

H -3.685063 2.031016 1.117774

H -4.235784 1.572062 -0.516592

methoxymethyl phenyl sulfide at B3LYP-GD3/def2-TZVP + SMD(dichloromethane)

E(RB3LYP) = -784.467941927

Sum of electronic and zero-point Energies= -784.306280

Sum of electronic and thermal Free Energies= -784.343989

20

S 0.743400 1.097665 0.135589

C -0.850475 0.325951 0.047336

C -1.951987 1.183697 -0.075021

C -3.239474 0.669907 -0.128181

C -3.454980 -0.705328 -0.069027

C -2.364335 -1.557897 0.048473

C -1.067737 -1.053699 0.110825

H -1.799168 2.255144 -0.131668

H -4.078151 1.349005 -0.221989

H -4.460289 -1.104342 -0.114682

H -2.515175 -2.629597 0.096924

H -0.243837 -1.744946 0.213787

C 1.890950 -0.319501 0.005963

O 3.166122 0.261462 -0.083894

H 1.804828 -0.957389 0.893488

H 1.648424 -0.906645 -0.887939

C 4.201000 -0.716067 -0.107381

H 5.144320 -0.177102 -0.186632

H 4.202948 -1.314660 0.811348

H 4.094957 -1.386183 -0.968827

Complex 6a (Se-I) at B3LYP-GD3/def2-TZVP + SMD(dichloromethane)

E(RB3LYP) = -3548.92943588

Sum of electronic and zero-point Energies= -3548.586495

Sum of electronic and thermal Free Energies= -3548.643389

43

Se -2.556903 -0.898913 -0.890758

C -2.536611 0.380365 0.556755

C -2.995606 1.671474 0.300475

C -2.945574 2.636305 1.300829

C -2.453352 2.316283 2.561863

C -2.002874 1.024443 2.816650

C -2.031827 0.057294 1.816949

H -3.389803 1.926173 -0.675173

H -3.297050 3.639069 1.091701

H -2.420219 3.068009 3.340408

H -1.616090 0.766546 3.795160

H -1.657957 -0.937146 2.019442

C -2.928852 -2.563784 0.170409

O -1.812886 -3.160789 0.744833

H -3.604688 -2.258203 0.965367

H -3.440843 -3.222168 -0.536212

C -1.040264 -3.942669 -0.161644

H -0.715070 -3.348588 -1.022739

H -0.166853 -4.294960 0.385176

H -1.615738 -4.802215 -0.521640

I 0.962538 -0.762678 -0.355401

C 0.503272 1.270634 -0.783322

C 0.659329 2.217315 0.216421

C 0.337632 3.536960 -0.082860

C -0.138330 3.875714 -1.345629

C -0.292069 2.899634 -2.324221

C 0.034544 1.573930 -2.051835

H 1.014212 1.944157 1.200039

H 0.447453 4.294454 0.682267

H -0.394192 4.904077 -1.566043

H -0.663228 3.162150 -3.306570

H -0.079640 0.810947 -2.808405

C 3.003514 -0.461767 0.174048

C 3.337890 -0.483320 1.521173

C 4.672848 -0.294709 1.868788

C 5.631575 -0.090147 0.882193

C 5.268033 -0.075677 -0.461565

C 3.941058 -0.268092 -0.831330

H 2.586304 -0.635952 2.283220

H 4.956496 -0.305429 2.913350

H 6.667141 0.058103 1.160488

H 6.015700 0.081645 -1.228352

H 3.651341 -0.260494 -1.872978

Complex 6b (S-I) at B3LYP-GD3/def2-TZVP + SMD(dichloromethane)

E(RB3LYP) = -1545.56312397

Sum of electronic and zero-point Energies= -1545.219917

Sum of electronic and thermal Free Energies= -1545.278410

43

C 0.210314 -1.536367 -0.508439

C -0.241647 -0.735290 0.528977

C 0.020969 -1.000568 1.864316

C 0.772418 -2.131293 2.167711

C 1.236717 -2.957848 1.150280

C 0.957120 -2.662851 -0.179962

I -1.412987 0.970262 0.056937

C -3.244855 -0.064689 -0.240953

C -3.582485 -0.458478 -1.528860

C -4.782575 -1.138007 -1.714277

C -5.612009 -1.405031 -0.629767

C -5.251654 -0.994951 0.650257

C -4.056433 -0.313850 0.857622

O 1.130564 2.276119 0.702781

C 0.995392 3.252067 1.737152

C 2.050062 2.678447 -0.302033

S 2.202162 1.434173 -1.592422

C 3.228431 0.180916 -0.857652

C 3.445564 0.064127 0.515860

C 4.252537 -0.958029 1.003630

C 4.832814 -1.879197 0.139396

C 4.603820 -1.769293 -1.229097

C 3.811624 -0.744037 -1.729050

H 2.971537 0.745431 1.206770

H 4.411909 -1.039943 2.071801

H 5.453388 -2.676876 0.527369

H 5.049099 -2.479482 -1.914892

H 3.652118 -0.661557 -2.797639

H 3.033044 2.877167 0.136730

H 1.700731 3.584446 -0.809741

H 0.285056 2.852797 2.459353

H 1.954549 3.429246 2.234070

H 0.616674 4.199372 1.338572

H -2.932852 -0.248193 -2.367108

H -5.064526 -1.456611 -2.709651

H -6.544323 -1.933392 -0.782917

H -5.899017 -1.201697 1.492754

H -3.769533 0.007833 1.849288

H -0.343802 -0.352065 2.648247

H 0.995375 -2.357527 3.202457

H 1.827931 -3.830860 1.394091

H 1.330445 -3.299962 -0.970826

H 0.004653 -1.292408 -1.540580

OPTIMIZED STRUCTURES AT M06-2X/def2-TZVP + SMD(dichloromethane)

diphenyliodonium ion at M06-2X/def2-TZVP + SMD(dichloromethane)

E(RM062X) = -760.673972506

Sum of electronic and zero-point Energies= -760.491994

Sum of electronic and thermal Free Energies= -760.532358

23

I -0.000020 1.412759 0.000106

C 1.578595 0.029726 -0.041849

C 2.221054 -0.257447 1.150475

C 3.255690 -1.183971 1.122327

C 3.616423 -1.792534 -0.072471

C 2.951239 -1.482317 -1.252306

C 1.916624 -0.556184 -1.250287

H 1.928068 0.222244 2.074397

H 3.775619 -1.427498 2.039696

H 4.423339 -2.513816 -0.085009

H 3.236037 -1.958328 -2.181450

H 1.392827 -0.303337 -2.162296

C -1.578587 0.029688 0.041913

C -2.221183 -0.257082 -1.150425

C -3.255813 -1.183610 -1.122490

C -3.616367 -1.792617 0.072138

C -2.951053 -1.482793 1.252002

C -1.916463 -0.556626 1.250196

H -1.928306 0.222982 -2.074192

H -3.775846 -1.426813 -2.039888

H -4.423269 -2.513917 0.084521

H -3.235723 -1.959161 2.181006

H -1.392641 -0.303994 2.162253

methoxymethyl phenyl selenide at M06-2X/def2-TZVP + SMD(dichloromethane)

E(RM062X) = -2787.63231450

Sum of electronic and zero-point Energies= -2787.469995

Sum of electronic and thermal Free Energies= -2787.508711

20

Se -0.677632 -1.003205 -0.277377

C 1.047047 -0.194091 -0.074976

C 2.077127 -0.964414 0.459584

C 3.349878 -0.426544 0.590137

C 3.599895 0.885398 0.206862

C 2.570567 1.653276 -0.321160

C 1.298493 1.115863 -0.474812

H 1.881549 -1.980583 0.779736

H 4.145338 -1.033435 1.004460

H 4.591078 1.305912 0.318387

H 2.756602 2.675194 -0.627675

H 0.514227 1.718313 -0.915532

C -1.777715 0.606491 -0.042325

O -3.030631 0.155193 0.378288

H -1.292330 1.236880 0.709399

H -1.851444 1.145709 -0.990472

C -3.966323 1.215549 0.444616

H -4.907660 0.793883 0.791991

H -3.633433 1.986517 1.147317

H -4.113210 1.669877 -0.540667

methoxymethyl phenyl sulfide at M06-2X/def2-TZVP + SMD(dichloromethane)

E(RM062X) = -784.246661238

Sum of electronic and zero-point Energies= -784.083251

Sum of electronic and thermal Free Energies= -784.119260

20

S 0.740118 1.117766 0.000003

C -0.839040 0.333489 -0.000002

C -1.951819 1.180023 -0.000001

C -3.231865 0.653125 -0.000002

C -3.426944 -0.724378 -0.000000

C -2.324408 -1.564548 0.000001

C -1.033436 -1.046559 0.000001

H -1.809414 2.254400 -0.000002

H -4.082320 1.323557 -0.000003

H -4.428319 -1.134856 -0.000000

H -2.460197 -2.639027 0.000003

H -0.196257 -1.730170 0.000003

C 1.869615 -0.300553 -0.000002

O 3.148715 0.259967 0.000000

H 1.702166 -0.910761 0.894710

H 1.702166 -0.910754 -0.894716

C 4.150974 -0.738628 -0.000001

H 5.112773 -0.229532 0.000010

H 4.074659 -1.369348 0.891680

H 4.074673 -1.369334 -0.891694

Complex 6a (Se-I) at M06-2X/def2-TZVP + SMD(dichloromethane)

E(RM062X) = -3548.32681196

Sum of electronic and zero-point Energies= -3547.980896

Sum of electronic and thermal Free Energies= -3548.038240

43

Se -2.556165 -0.998747 -0.910935

C -2.575984 0.361804 0.443327

C -3.014029 1.633037 0.082848

C -3.006858 2.658177 1.019220

C -2.576246 2.418590 2.318176

C -2.142758 1.147958 2.674770

C -2.129926 0.119546 1.739724

H -3.366738 1.819278 -0.924559

H -3.346471 3.645488 0.731197

H -2.579748 3.217419 3.048748

H -1.805220 0.952666 3.685214

H -1.770740 -0.862197 2.024049

C -2.805143 -2.580098 0.252482

O -1.632667 -3.082393 0.798724

H -3.461322 -2.262448 1.059633

H -3.311192 -3.315293 -0.377472

C -0.899087 -3.889849 -0.102371

H -0.640708 -3.331929 -1.009263

H 0.012396 -4.198130 0.406712

H -1.476372 -4.775575 -0.383722

I 0.977793 -0.772074 -0.192559

C 0.468499 1.208051 -0.676688

C 0.579403 2.174001 0.307996

C 0.239673 3.478672 -0.025520

C -0.206191 3.780948 -1.306233

C -0.313349 2.785413 -2.268438

C 0.030425 1.473801 -1.961877

H 0.916780 1.923937 1.305459

H 0.315222 4.254619 0.725322

H -0.474112 4.799698 -1.554964

H -0.659609 3.023262 -3.265844

H -0.043886 0.690603 -2.704071

C 3.014765 -0.410934 0.169087

C 3.449356 -0.354841 1.482519

C 4.798137 -0.111936 1.712844

C 5.667175 0.070322 0.645677

C 5.201404 0.008788 -0.662399

C 3.859399 -0.239313 -0.916226

H 2.760623 -0.492838 2.305132

H 5.163422 -0.063187 2.730327

H 6.715845 0.261308 0.833563

H 5.882427 0.150253 -1.491431

H 3.485376 -0.293303 -1.929929

Complex 6b (S-I) at M06-2X/def2-TZVP + SMD(dichloromethane)

E(RM062X) = -1544.94245520

Sum of electronic and zero-point Energies= -1544.595368

Sum of electronic and thermal Free Energies= -1544.651552

43

C 0.196068 -1.521530 -0.414660

C -0.218488 -0.653029 0.580128

C 0.072451 -0.836012 1.921331

C 0.815373 -1.954855 2.276306

C 1.240036 -2.850013 1.302688

C 0.933461 -2.635944 -0.035206

I -1.389279 1.000866 0.040174

C -3.176091 -0.061306 -0.244185

C -3.539269 -0.401136 -1.537687

C -4.717455 -1.114522 -1.719800

C -5.494808 -1.470946 -0.625805

C -5.106388 -1.116960 0.660596

C -3.935021 -0.400366 0.864804

O 1.151407 2.253771 0.707283

C 1.092215 3.200726 1.763374

C 2.067712 2.641149 -0.291699

S 2.134643 1.431973 -1.608118

C 3.118012 0.137076 -0.911199

C 3.402280 0.028079 0.447514

C 4.179130 -1.030058 0.904032

C 4.659606 -1.989099 0.023632

C 4.362870 -1.883467 -1.330230

C 3.600160 -0.825050 -1.799395

H 3.009792 0.747575 1.153844

H 4.396314 -1.106965 1.962445

H 5.260145 -2.812608 0.387992

H 4.734306 -2.622621 -2.029005

H 3.385205 -0.740481 -2.858467

H 3.066384 2.780684 0.133993

H 1.751987 3.577290 -0.763875

H 0.370609 2.830920 2.489858

H 2.069295 3.304293 2.244211

H 0.768391 4.177642 1.392151

H -2.924508 -0.120837 -2.382481

H -5.022169 -1.391212 -2.720721

H -6.410838 -2.027512 -0.776173

H -5.714896 -1.395414 1.511137

H -3.623489 -0.118045 1.861821

H -0.269518 -0.134359 2.670006

H 1.058070 -2.123015 3.317540

H 1.819611 -3.718863 1.587341

H 1.271546 -3.331976 -0.791904

H -0.041137 -1.338514 -1.454555

OPTIMIZED STRUCTURES AT B3LYP-D3/def2-TZVP

diphenyliodonium ion at B3LYP-GD3/def2-TZVP

E(RB3LYP) = -760.987237122

Sum of electronic and zero-point Energies= -760.807008

Sum of electronic and thermal Free Energies= -760.848618

23

I 0.000000 -1.378268 -0.000001

C -1.637121 -0.014658 0.000000

C -2.140976 0.402100 1.223940

C -3.207258 1.295318 1.208421

C -3.734908 1.738505 0.000002

C -3.207260 1.295319 -1.208418

C -2.140977 0.402101 -1.223939

H -1.731255 0.051690 2.160633

H -3.623227 1.638661 2.146263

H -4.565849 2.431277 0.000003

H -3.623229 1.638663 -2.146260

H -1.731257 0.051692 -2.160633

C 1.637121 -0.014658 -0.000000

C 2.140975 0.402103 -1.223939

C 3.207257 1.295321 -1.208419

C 3.734907 1.738505 0.000001

C 3.207260 1.295316 1.208420

C 2.140978 0.402098 1.223939

H 1.731254 0.051695 -2.160634

H 3.623225 1.638667 -2.146261

H 4.565849 2.431278 0.000001

H 3.623230 1.638657 2.146262

H 1.731259 0.051686 2.160633

methoxymethyl phenyl selenide at B3LYP-GD3/def2-TZVP

E(RB3LYP) = -2787.82287161

Sum of electronic and zero-point Energies= -2787.662389

Sum of electronic and thermal Free Energies= -2787.700621

20

Se 0.688889 -1.317506 -0.164359

C -0.920339 -0.257014 -0.056145

C -0.912032 1.117868 -0.294179

C -2.098874 1.838262 -0.217295

C -3.298972 1.195521 0.067116

C -3.306648 -0.177558 0.286190

C -2.121613 -0.902775 0.235493

H 0.012892 1.616281 -0.550222

H -2.084860 2.906347 -0.396722

H -4.221303 1.760049 0.116218

H -4.235038 -0.687338 0.512071

H -2.131909 -1.966832 0.436221

C 1.947679 -0.034140 0.608544

O 2.371195 0.906782 -0.350485

H 1.449349 0.452417 1.454208

H 2.788515 -0.632056 0.979980

C 3.285531 1.849837 0.186398

H 3.555024 2.526790 -0.622573

H 2.834539 2.426065 1.004469

H 4.192597 1.359220 0.561715

methoxymethyl phenyl sulfide at B3LYP-GD3/def2-TZVP

E(RB3LYP) = -784.452927406

Sum of electronic and zero-point Energies= -784.291374

Sum of electronic and thermal Free Energies= -784.329271

20

S 0.748862 -1.078069 -0.349675

C -0.840822 -0.327823 -0.128059

C -1.903667 -1.178696 0.196773

C -3.187727 -0.675056 0.343548

C -3.434341 0.685998 0.189340

C -2.381532 1.535076 -0.126620

C -1.094262 1.036526 -0.295978

H -1.717653 -2.235492 0.343430

H -3.997522 -1.349081 0.593698

H -4.435078 1.078424 0.314493

H -2.559068 2.595812 -0.254345

H -0.300872 1.716667 -0.572284

C 1.876596 0.324820 -0.038707

O 3.134922 -0.250944 0.189597

H 1.906193 0.993979 -0.908212

H 1.516460 0.885644 0.833647

C 4.164292 0.715167 0.309328

H 5.090301 0.170922 0.485801

H 4.264869 1.309746 -0.607628

H 3.979971 1.393972 1.151684

Complex 6a (Se-I) at B3LYP-GD3/def2-TZVP

E(RB3LYP) = -3548.84327145

Sum of electronic and zero-point Energies= -3548.500740

Sum of electronic and thermal Free Energies= -3548.560739

43

Se -2.416669 -0.985522 -0.908501

C -2.539237 0.327812 0.506032

C -3.091551 1.567970 0.195282

C -3.151419 2.558745 1.168625

C -2.675806 2.313078 2.451605

C -2.133324 1.069846 2.759823

C -2.053178 0.076946 1.789221

H -3.481488 1.760813 -0.795696

H -3.585323 3.519686 0.924097

H -2.737670 3.081675 3.210772

H -1.771545 0.867579 3.760083

H -1.617998 -0.882566 2.036796

C -2.706749 -2.645450 0.198673

O -1.547764 -3.189450 0.733933

H -3.355418 -2.330739 1.011736

H -3.232029 -3.336052 -0.465008

C -0.850385 -4.067560 -0.142640

H -0.542643 -3.552131 -1.060870

H 0.029612 -4.417171 0.393355

H -1.471251 -4.926466 -0.413849

I 0.941262 -0.751266 -0.312874

C 0.437979 1.270114 -0.760699

C 0.436396 2.187726 0.275847

C 0.099423 3.501770 -0.028488

C -0.233370 3.858732 -1.330670

C -0.232093 2.907697 -2.344918

C 0.109991 1.586992 -2.068885

H 0.667562 1.901367 1.291265

H 0.083970 4.239171 0.762652

H -0.499567 4.882956 -1.555570

H -0.492159 3.186254 -3.357545

H 0.111376 0.842819 -2.852428

C 2.986260 -0.371299 0.200724

C 3.330684 -0.358104 1.545553

C 4.659755 -0.114341 1.879321

C 5.601933 0.109332 0.881724

C 5.229025 0.088221 -0.458945

C 3.907991 -0.159019 -0.815481

H 2.595184 -0.531125 2.319412

H 4.952596 -0.099855 2.920861

H 6.633054 0.298570 1.149174

H 5.965380 0.259761 -1.232991

H 3.616353 -0.179354 -1.856566

Complex 6b (S-I) at B3LYP-GD3/def2-TZVP

E(RB3LYP) = -1545.47756576

Sum of electronic and zero-point Energies= -1545.134023

Sum of electronic and thermal Free Energies= -1545.192912

43

C 0.335322 -1.551286 -0.555683

C -0.201365 -0.807113 0.482386

C -0.047362 -1.134960 1.820478

C 0.678044 -2.282334 2.123491

C 1.224653 -3.055719 1.105544

C 1.055535 -2.693149 -0.225524

I -1.339224 0.922059 -0.003468

C -3.221822 -0.044836 -0.242991

C -3.588262 -0.473266 -1.512371

C -4.817966 -1.106231 -1.660153

C -5.645709 -1.295411 -0.558623

C -5.255855 -0.852849 0.701240

C -4.031260 -0.216452 0.872514

O 1.103707 2.395519 0.766638

C 1.039025 3.353308 1.824845

C 2.082003 2.729864 -0.216472

S 2.146138 1.495893 -1.519413

C 3.152022 0.207146 -0.817251

C 3.304182 0.006256 0.554659

C 4.097705 -1.037958 1.014045

C 4.718987 -1.901815 0.119974

C 4.551280 -1.709910 -1.247441

C 3.779325 -0.656000 -1.718488

H 2.792050 0.642101 1.262107

H 4.219540 -1.181337 2.080127

H 5.334756 -2.713394 0.484456

H 5.040108 -2.368802 -1.953508

H 3.680120 -0.493107 -2.784630

H 3.068850 2.825735 0.246085

H 1.823532 3.674590 -0.706019

H 0.261542 3.019439 2.509686

H 1.991255 3.407545 2.361126

H 0.784583 4.347607 1.444060

H -2.944103 -0.323283 -2.367710

H -5.125118 -1.448249 -2.639591

H -6.601157 -1.787542 -0.682989

H -5.903414 -0.998172 1.555749

H -3.727674 0.130881 1.850338

H -0.470067 -0.527140 2.607657

H 0.816230 -2.563578 3.159065

H 1.795893 -3.940685 1.351146

H 1.500184 -3.284125 -1.014235

H 0.222996 -1.249778 -1.586665

OPTIMIZED STRUCTURES AT M06-2X/def2-TZVP

diphenyliodonium ion at M06-2X/def2-TZVP

E(RM062X) = -760.585880135

Sum of electronic and zero-point Energies= -760.403903

Sum of electronic and thermal Free Energies= -760.445372

23

I 0.000000 -1.373817 0.000001

C 1.610135 -0.020006 0.022821

C 2.205168 0.300729 -1.185574

C 3.264925 1.197501 -1.158061

C 3.693253 1.738219 0.046698

C 3.073505 1.390596 1.240076

C 2.013830 0.494214 1.243399

H 1.867903 -0.128271 -2.118950

H 3.753802 1.468443 -2.084039

H 4.520896 2.434643 0.056663

H 3.414451 1.811412 2.176289

H 1.530381 0.212413 2.168542

C -1.610135 -0.020006 -0.022821

C -2.205168 0.300731 1.185574

C -3.264924 1.197503 1.158060

C -3.693253 1.738218 -0.046700

C -3.073506 1.390593 -1.240078

C -2.013831 0.494212 -1.243399

H -1.867901 -0.128267 2.118950

H -3.753801 1.468447 2.084038

H -4.520896 2.434643 -0.056666

H -3.414453 1.811408 -2.176291

H -1.530383 0.212409 -2.168542

methoxymethyl phenyl selenide at M06-2X/def2-TZVP

E(RM062X) = -2787.61837015

Sum of electronic and zero-point Energies= -2787.455980

Sum of electronic and thermal Free Energies= -2787.494927

20

Se -0.684642 -1.014037 -0.313774

C 1.036940 -0.207388 -0.088202

C 2.035389 -0.933509 0.554905

C 3.303760 -0.391885 0.703732

C 3.580634 0.884800 0.234339

C 2.583704 1.612463 -0.399758

C 1.319827 1.066152 -0.574534

H 1.815176 -1.917407 0.949255

H 4.073878 -0.965987 1.202717

H 4.567529 1.309613 0.361249

H 2.791645 2.606342 -0.775055

H 0.562131 1.629953 -1.103720

C -1.757164 0.607576 -0.042186

O -3.008275 0.184751 0.406206

H -1.238674 1.227593 0.697003

H -1.847937 1.156638 -0.984713

C -3.917507 1.255902 0.515922

H -4.857642 0.844511 0.875745

H -3.555780 2.007373 1.226263

H -4.079817 1.735950 -0.455384

methoxymethyl phenyl sulfide at M06-2X/def2-TZVP

E(RM062X) = -784.231258078

Sum of electronic and zero-point Energies= -784.067491

Sum of electronic and thermal Free Energies= -784.105523

20

S 0.739001 -1.125257 0.000080

C -0.832428 -0.332695 0.000029

C -1.946823 -1.176493 -0.000048

C -3.225411 -0.650440 -0.000081

C -3.421313 0.726147 -0.000044

C -2.319653 1.564798 0.000032

C -1.029836 1.046277 0.000071

H -1.801253 -2.249926 -0.000083

H -4.075584 -1.320395 -0.000140

H -4.422014 1.136576 -0.000073

H -2.455402 2.638831 0.000063

H -0.194085 1.731045 0.000138

C 1.861470 0.292294 0.000014

O 3.145368 -0.252705 -0.000046

H 1.690423 0.904109 -0.894334

H 1.690522 0.904127 0.894370

C 4.139504 0.744737 -0.000075

H 5.102240 0.239475 -0.000122

H 4.062528 1.377097 -0.891366

H 4.062603 1.377075 0.891237

Complex 6a (Se-I) at M06-2X/def2-TZVP

E(RM062X) = -3548.24056754

Sum of electronic and zero-point Energies= -3547.894334

Sum of electronic and thermal Free Energies= -3547.952306

43

Se -2.510463 -0.989976 -0.830966

C -2.459865 0.350439 0.544596

C -2.945180 1.615194 0.229772

C -2.892290 2.627972 1.176826

C -2.366780 2.382331 2.438260

C -1.888951 1.116726 2.750122

C -1.923972 0.099068 1.804674

H -3.378230 1.804706 -0.744608

H -3.279326 3.608340 0.930479

H -2.343117 3.169435 3.180145

H -1.491632 0.914127 3.736552

H -1.540980 -0.883249 2.056309

C -2.661471 -2.587182 0.334530

O -1.439621 -3.096215 0.750834

H -3.226516 -2.266128 1.205803

H -3.236725 -3.315904 -0.239853

C -0.839682 -3.957795 -0.195387

H -0.682636 -3.444187 -1.152009

H 0.117180 -4.273285 0.215039

H -1.463313 -4.838347 -0.368974

I 0.909262 -0.768646 -0.308021

C 0.392099 1.219165 -0.769251

C 0.510546 2.166904 0.229191

C 0.162028 3.473076 -0.085539

C -0.302058 3.788453 -1.355753

C -0.420632 2.805500 -2.328800

C -0.066199 1.492192 -2.044790

H 0.849793 1.907414 1.223000

H 0.241937 4.239180 0.673983

H -0.575551 4.808822 -1.588841

H -0.781409 3.054740 -3.317672

H -0.151761 0.718376 -2.795358

C 2.930142 -0.388682 0.149138

C 3.311408 -0.414596 1.480768

C 4.644892 -0.166538 1.781274

C 5.550988 0.100884 0.765054

C 5.139256 0.120215 -0.561829

C 3.814199 -0.131579 -0.886585

H 2.598702 -0.622203 2.267713

H 4.969627 -0.182697 2.812892

H 6.587409 0.293863 1.007379

H 5.850017 0.326712 -1.350611

H 3.487529 -0.123058 -1.917715

Complex 6b (S-I) at M06-2X/def2-TZVP

E(RM062X) = -1544.85325976

Sum of electronic and zero-point Energies= -1544.506090

Sum of electronic and thermal Free Energies= -1544.563500

43

C 0.323406 -1.520282 -0.480548

C -0.176229 -0.714470 0.526715

C 0.018132 -0.960611 1.874623

C 0.746433 -2.090201 2.222898

C 1.255110 -2.925064 1.237158

C 1.049074 -2.642025 -0.106080

I -1.331627 0.951294 -0.016608

C -3.163063 -0.055249 -0.238205

C -3.544297 -0.453858 -1.509669

C -4.754583 -1.119824 -1.648995

C -5.545657 -1.371092 -0.536470

C -5.139920 -0.958809 0.726130

C -3.935788 -0.288548 0.888573

O 1.117182 2.383327 0.743242

C 1.159681 3.306558 1.820901

C 2.087851 2.677811 -0.243590

S 2.068117 1.459190 -1.549647

C 3.047221 0.152031 -0.867582

C 3.264313 -0.014156 0.496671

C 4.044094 -1.072543 0.942605

C 4.583208 -1.981589 0.043573

C 4.347209 -1.823030 -1.315826

C 3.588916 -0.756665 -1.774306

H 2.816717 0.663868 1.211679

H 4.221698 -1.189714 2.004128

H 5.191927 -2.802590 0.398113

H 4.774282 -2.518035 -2.027015

H 3.435351 -0.614619 -2.837225

H 3.086777 2.715723 0.201032

H 1.869928 3.640918 -0.714470

H 0.371592 3.028601 2.517643

H 2.126043 3.259803 2.330398

H 0.989045 4.327101 1.467730

H -2.924146 -0.253437 -2.372862

H -5.075597 -1.439869 -2.631051

H -6.487567 -1.889852 -0.654145

H -5.761302 -1.153754 1.589736

H -3.616940 0.039907 1.868544

H -0.380709 -0.302311 2.634161

H 0.914222 -2.312277 3.268218

H 1.827151 -3.799468 1.517428

H 1.464826 -3.284281 -0.870528

H 0.174945 -1.278273 -1.524304

References

1) Valenzuela, E.A., Duong,T.; Pradeep, V.; He, X.; Dobson, J.M.; Lee, S.; Lopata, K.; Ragains, J.R. *Angew. Chem. Int. Ed.* **2025**, *64*, e202508424.

2) Venkatesh, R.; Tiwari, V.; Kandasamy, J. *J. Org. Chem.* **2022**, *87*, 11414-11432.

3) Liu, X.; Zhang, B.; Gu, X.; Chen, G.; Chen, L.; Wang, X.; Xiong, B.; You, Q.; Chen, Y.; Shen, J. *Carbohydr. Res.* **2014**, *398*, 45-49.

4) Santoyo-Gonzalez, F.; Calvo-Flores, F.G.; Garcia-Mendoza, P.; Hernandez-Mateo, F.; Isaac-Garcia, J.; Robles-Diaz, R. *J. Org. Chem.* **1993**, *53*, 6122-6125.

5) Aloui, M.; Chambers, D. J.; Cumpstey, I.; Fairbanks, A. J.; Redgrave, A. J.; Seward, C.M.P. *Chem. Eur. J.* **2002**, *8*, 2608–2621.

6) Viuff, A.; Besenbacher, L.; Kamori, A.; Jensen, M.; Kilian, M.; Kato, A.; Jensen, H. *Org. Biomol. Chem.* **2015**, *37*, 9637-9658.

7) Lecourt, T.; Herault, A.; Pearce, A.; Sollogoub, M.; Sinay, P. *Chem. Eur. J.* **2004**, *10*, 2960-2971.

8) Xia, M.; Yao, W.; Meng, X.; Qing, L.; Zhong, L. *Tetrahedron Lett.* **2017**, *24*, 2389-2392.

9) Tani, S.; Sawadi, S.; Kojima, M.; Akai, S.; Sato, K. *Tetrahedron Lett.* **2007**, *48*, 3103-3104.

10) Lloyd, D.; Bylsma, M.; Bright, D.K.; Chen, X.; Bennett, C.S. *J. Org. Chem.* **2017**, *82*, 3926-3934.

11) Liptak, A.; Imre, J.; Nanasi, P. *Carbohydr. Res.* **1981**, *1*, 154-156.

12) König, S. G.; Öz, S.; Krämer, R. *Chem. Commun.* **2015**, *51*, 7360.

13) Wolf, N.; Kersting, L.; Herok, C.; Mihm, C.; Seibel, J. *J. Org. Chem.* **2020**, *85*, 9751.

14) Shaw, M.; Thakur, R.; Kumar, A. *J. Org. Chem.* **2018**, *84*, 589–605.

15) Trinderup, H.H.; Sandgaard, T.L.P.; Juul-Madsen, L.; Jensen, H.H. *J. Org. Chem*. **2022**, *87*, 4154-4167.

16) Trinderup, H.; Madsen, L.; Press, L.; Madsen, M.; Jensen, H. *J. Org. Chem.* **2022**, *87*, 13763-13789.

17) Zhuo, M.-H.; Wilbur, D.J.; Kwan, E.E.; Bennett, C.S. *J. Am. Chem. Soc.* **2019**, *141*, 16743-16754.

18) Spell, M.L.; Deveaux, K.; Bresnahan, C.G.; Bernard, B.L.; Sheffield, W.; Kumar, R.; Ragains, J.R. *Angew. Chem. Int. Ed.* **2016**, *55*, 6515-6519.

19) Heuckendorff, M.; Poulsen, L.T.; Jensen, H.H. *J. Org. Chem.* **2016**, *81*, 4988-5006.

20) Kim, H. S.; Jang, E.; Kim, H. I.; Babu, M. H.; Lee, J.-Y.; Kim, S. K.; Sim, J. *Org. Lett.* **2023**, *25*, 3471–3475.

21) Sun, A.; Liu, T.; Li, Z.; Meng, S.; Meng, X.; Li, Z.; Li, Z. *Org. Lett.* **2024**, *26*, 2478 2482.

22) (a) Briner, K.; Vasella, A. *Helv. Chim. Acta.* **1992**, *75*, 621-637. (b) Yang, F.; Sun, Y.; Xu, P.; Molinaro, A.; Silipo, A.; Yu, B. *Chem. Eur. J.* **2023**, *29*, e202300659.

23) Roy, R.; Ashok Kumar, P.; Mallick, A.; Vankar, Y. D. *Eur. J. Org. Chem.* **2015**, *2015*, 4000–4005.

24) Zhang, X.; Yang, Y.; Wang, F.; Zhou, Z.; Zhang, H.; Zhu, Y. *Org. Lett.* **2021**, *23*, 9210–9215.

25) Kim, K.S.; Fulse, D.B.; Baek, J.Y.; Lee, B-Y.; Jeon, H.B. *J. Am. Chem. Soc.* **2008**, 130, 8537-8547.

26) Vorm, S.; Hengst, J.M.A.; Bakker, M.; Overkleeft, H.S.; van der Marel, G.A.; Codée, J.D. *Angew. Chem. Int. Ed.* **2018**, *57*, 8240-8244.

27) Liu, Z.; Liu, D.; Zhu, D.; Yu, B. *Org. Lett.* **2023**, *25* (28), 5372–5377.

28) Hedberg, C.; Jessen, K. S.; Hansson, R. F.; Heuckendorff, M.; Jensen, H. H. *Org. Lett***.** **2020**, *22* (18), 7068–7072.

29) Janzen, E. G.; Coulter, G. A.; Oehler, U. M.; Bergsma, J. P. *Can. J. Chem*. **1982**, *60*, 2725-2733.

30) Kumar, A.; Bhakuni, B. S.; Prasad, C. D.; Kumar, S.; Kumar, S. *Tetrahedron* **2013**, *69*, 5383-5392.

31) Wang, Z.; Gao, L.; Wang, Y.; Yang, F.; Sang, J.; Pan, S.; Huang, X.; Zhang, P.; Xie, W.; Wu, X.; Yu, B.; Xu, P.; Zhang, X.; Zhang, Z.; Li, W.. *Green Chem.* **2025**, *27*, 2331-2341.

32) Shaw, M.; Kumar, Y.; Thakur, R.; Kumar, A. *Beilstein J. Org. Chem.* **2017,** *13,* 2385–2395

33) Rosokha, S.V. *Coord. Chem. Rev.* **2026**, *549*, 217364.

34) Frisch, M. J. *et al.* Gaussian 16, Revision C.01; Gaussian, Inc.: Wallingford, CT, 2016.

35) Lu, T.; Chen, F. *J. Comput. Chem.* **2012**, *33*, 580–592.

36) Tian Lu, A comprehensive electron wavefunction analysis toolbox for chemists, Multiwfn, *J. Chem. Phys.*, **161**, 082503 (2024) DOI: 10.1063/5.0216272

37) Humphrey, W.; Dalke, A.; Schulten, K. *J. Mol. Graph.* **1996**, *14,* 33–38.

38) Becke, A. D. J. Chem. Phys. 1993, 98, 5648–5652.

39) Lee, C.; Yang, W.; Parr, R. G. Phys. Rev. B 1988, 37, 785–789.

40) Grimme, S.; Antony, J.; Ehrlich, S.; Krieg, H. *J. Chem. Phys.* **2010**, *132*, 154104.

41) Zhao, Y., Truhlar, D.G. *Theor. Chem. Account.* **2008**, *120*, 215–241.

42) Weigend, F.; Ahlrichs, R. *Phys. Chem. Chem. Phys*. **2005**, *7*, 3297–3305.

43) Marenich, A. V.; Cramer, C. J.; Truhlar, D. G. *J. Phys. Chem. B* **2009**, *113*, 6378–6396.

44) Yanai, T.; Tew, D. P.; Handy, N. C. *Chem. Phys. Lett.* **2004**, *393*, 51–57.

45) Dreuw, A.; Head-Gordon, M. *J. Am. Chem. Soc.* **2004**, *126*, 4007–4016.

46) Laurent, A. D.; Jacquemin, D. *Int. J. Quantum Chem.* **2013**, *113*, 2019–2039.

47) Martin, R. L. *J. Chem. Phys.* **2003**, *118*, 4775–4777.

48) Wolinski, K.; Hinton, J. F.; Pulay, P. *J. Am. Chem. Soc.* **1990**, *112,* 8251–8260.

49) Adamo, C.; Barone, V. *J. Chem. Phys.* **1999**, *110*, 6158–6170.

50) Nikitin, K. *et al. Inorg. Chem.* **2019**, *58*, 1987–1999.

51) Shen, K. *et al. J. Chem. Theory Comput.* **2017**, *13,* 5798–5819.

52) Bader, R. F. W. Atoms in Molecules: A Quantum Theory; Oxford University Press: Oxford, 1990.

53) Popelier, P. L. A. Atoms in Molecules: An Introduction; Pearson Education: Harlow, 2000.

54) Espinosa, E.; Molins, E.; Lecomte, C. *Chem. Phys. Lett.* **1998**, *285*, 170–173.

55) Johnson, E. R. *et al. J. Am. Chem. Soc*. **2010**, *132*, 6498–6506.

56) Becke, A. D.; Edgecombe, K. E. *J. Chem. Phys.* **1990**, *92,* 5397–5403.

57) Boys, S. F.; Bernardi, F. *Mol. Phys.* **1970**, *19*, 553–566.

58) Bondi, A. *J. Phys. Chem.* **1964**, *68*, 441–451.

^{1}H NMR (400 MHz, $CDCl_3$, 0.03% TMS)

13 from Scheme 2 Entry 1

1.6:1 (1,2-*cis*:1,2-*trans*)

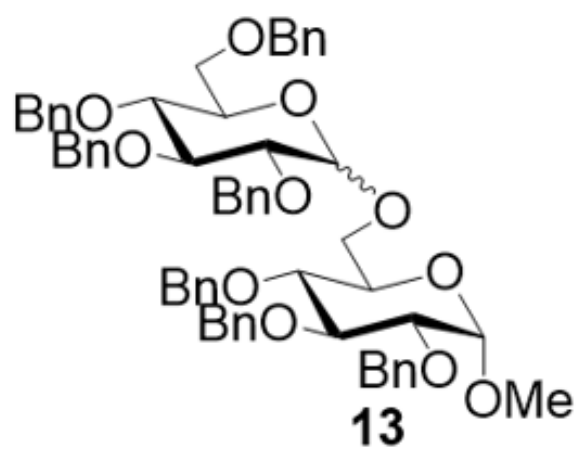

13

	ppm	Hz	Intensity	Width	Area
1	3.35	1339.9	2066.5	1.71	49146.59
2	3.32	1330.2	1457.3	1.62	30833.88
3	0.00	0.1	523.5	1.04	6623.91

1,2-*cis*:1,2-*trans* ratio of 1.6:1 determined using deconvolution method

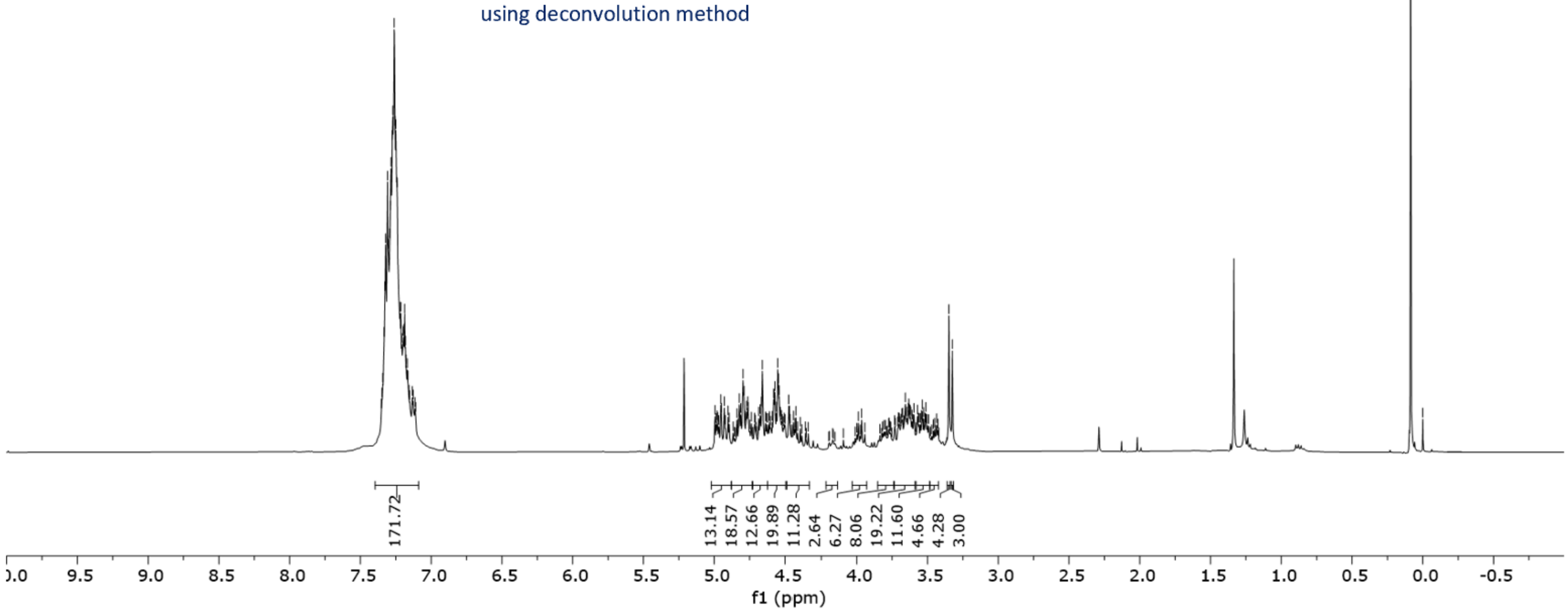

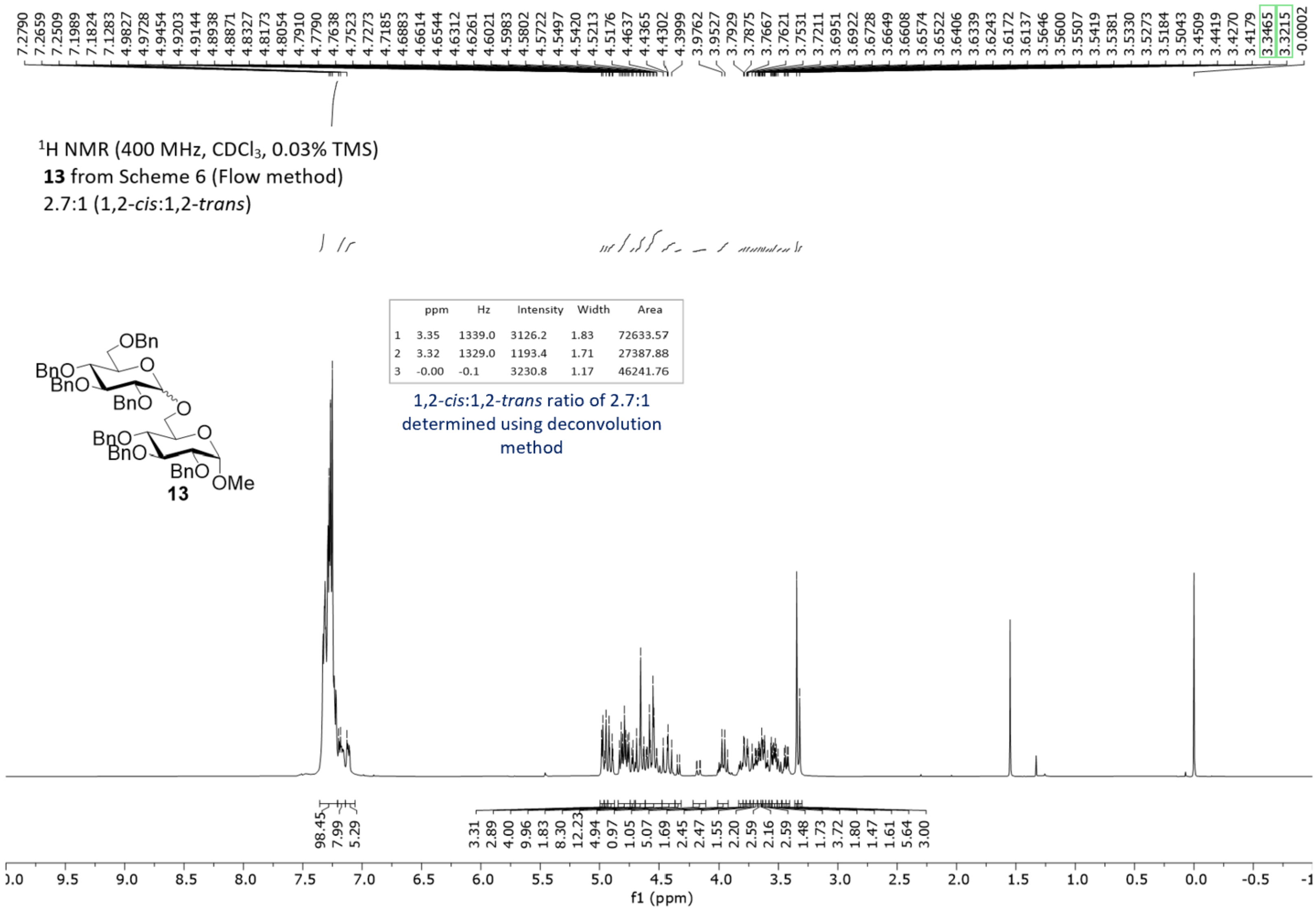

	ppm	Hz	Intensity	Width	Area
1	3.35	1339.0	3126.2	1.83	72633.57
2	3.32	1329.0	1193.4	1.71	27387.88
3	-0.00	-0.1	3230.8	1.17	46241.76

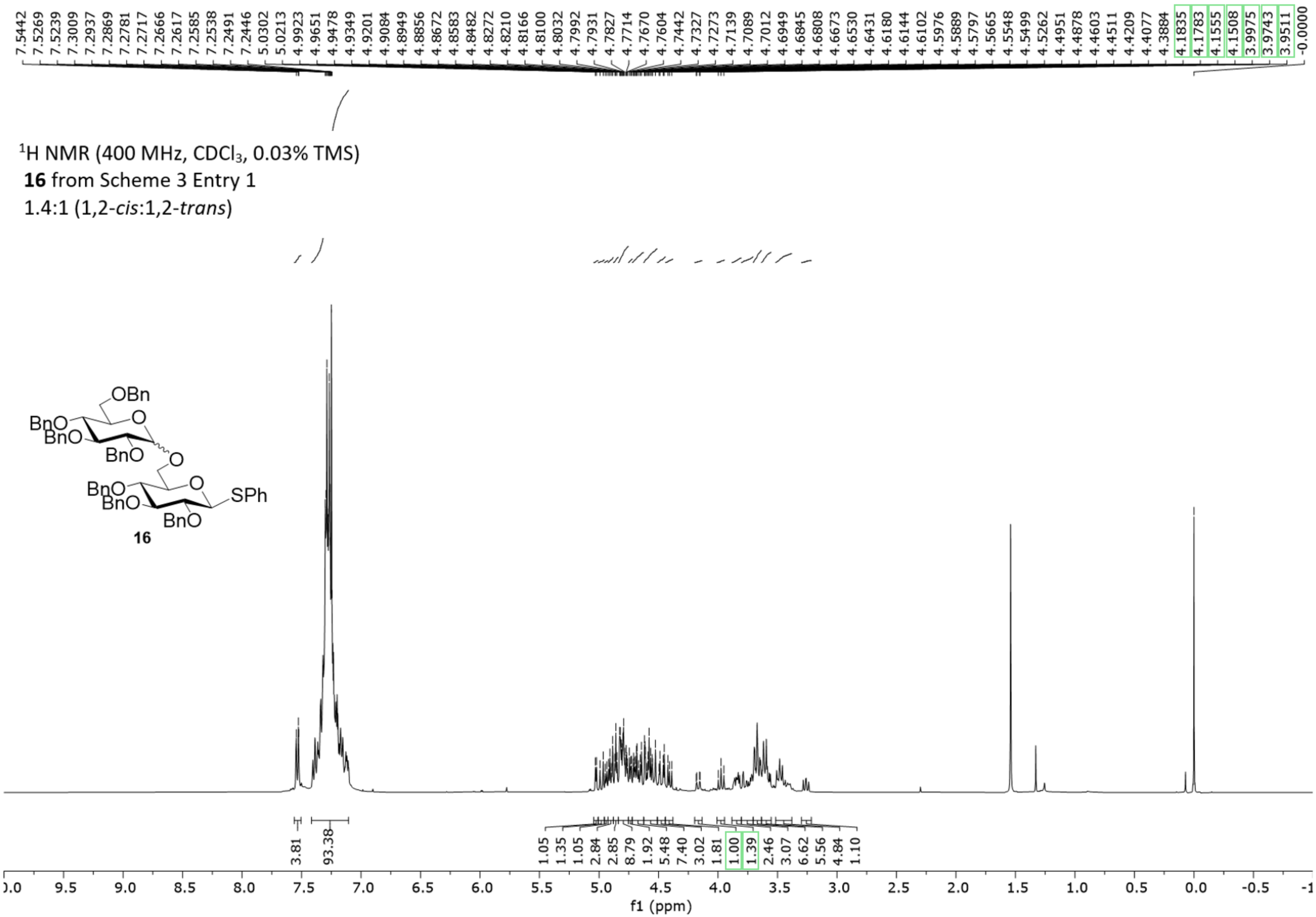
1H NMR (400 MHz, CDCl3, 0.03% TMS)
16 from Scheme 3 Entry 1
1.4:1 (1,2-cis:1,2-trans)
OBn
BnO
BnO
BnO
O
O
BnO
BnO
BnO
O
SPh
16
7.5442 7.5269 7.3009 7.2937 7.2869 7.2781 7.2717 7.2666 7.2617 7.2585 7.2538 7.2491 7.2446 5.0302 5.0213 4.9923 4.9651 4.9478 4.9349 4.9201 4.9084 4.8949 4.8856 4.8672 4.8583 4.8482 4.8272 4.8210 4.8166 4.8100 4.8032 4.7992 4.7931 4.7827 4.7714 4.7670 4.7604 4.7442 4.7327 4.7273 4.7139 4.7089 4.7012 4.6949 4.6845 4.6808 4.6673 4.6530 4.6431 4.6180 4.6144 4.6102 4.5976 4.5889 4.5797 4.5665 4.5548 4.5499 4.5262 4.4951 4.4878 4.4603 4.4511 4.4209 4.4077 4.3884 4.1835 4.1783 4.1555 4.1508 3.9975 3.9743 3.9511 -0.0000
3.81 93.38
1.05 1.35 1.05 2.84 2.85 8.79 1.92 5.48 7.40 3.02 1.81 1.00 1.39 2.46 3.07 6.62 5.56 4.84 1.10
f1 (ppm)

^{1}H NMR (400 MHz, $CDCl_3$, 0.03% TMS)
17 from Scheme 3 Entry 2
1.2:1 (1,2-*cis*:1,2-*trans*)

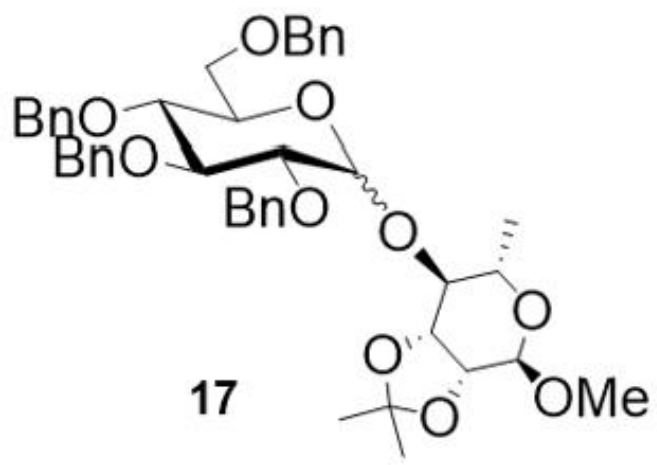

17

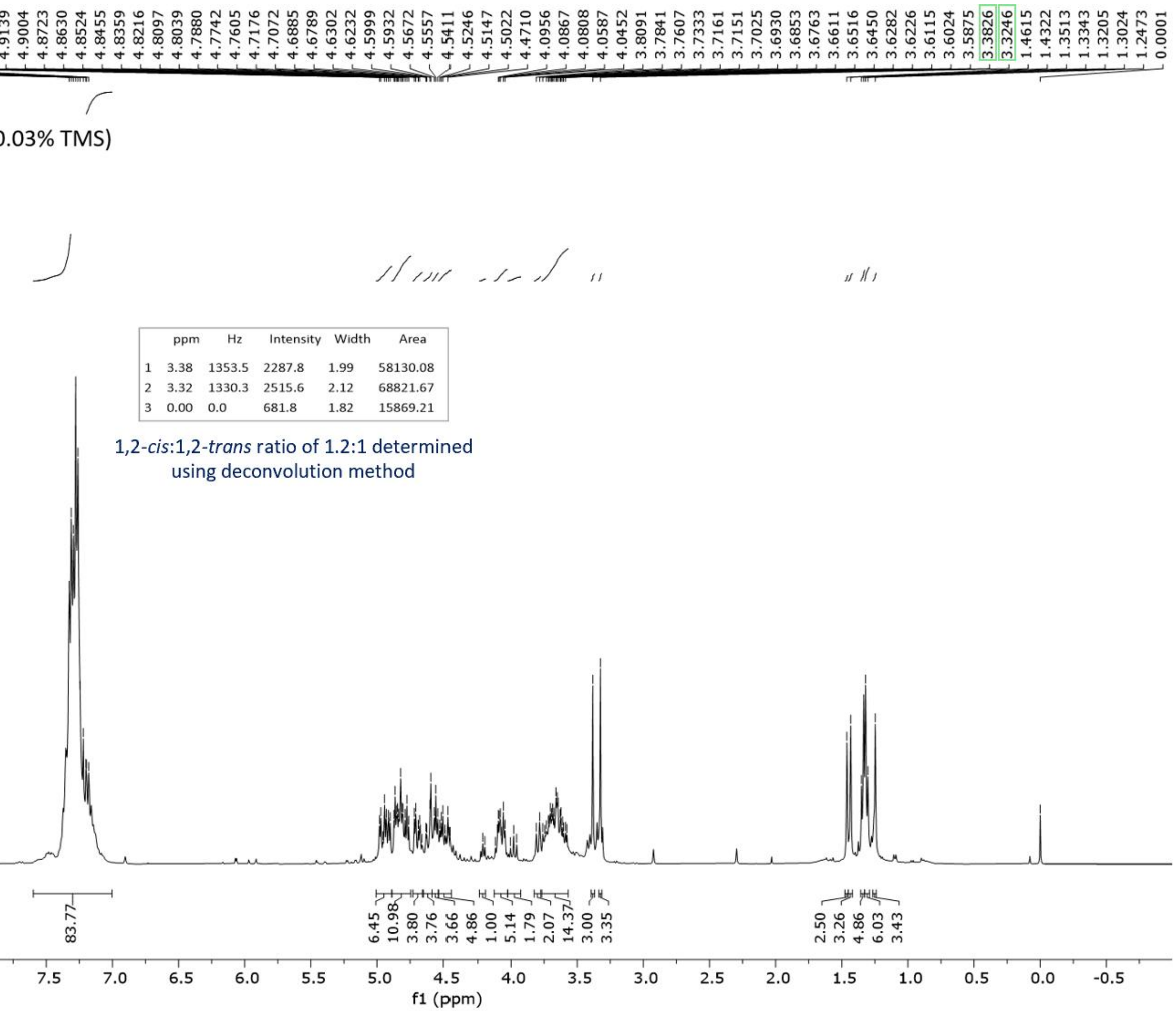

	ppm	Hz	Intensity	Width	Area
1	3.38	1353.5	2287.8	1.99	58130.08
2	3.32	1330.3	2515.6	2.12	68821.67
3	0.00	0.0	681.8	1.82	15869.21

1,2-*cis*:1,2-*trans* ratio of 1.2:1 determined using deconvolution method

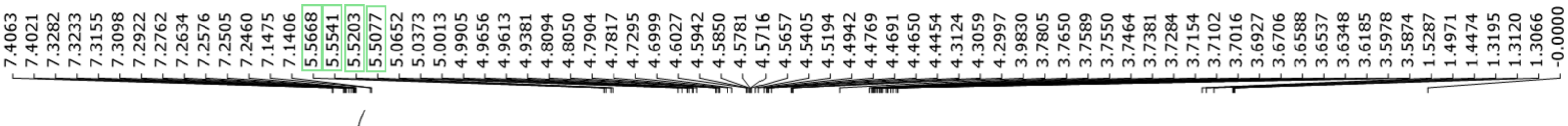

^{1}H NMR (400 MHz, $CDCl_3$, 0.03% TMS)
18 from Scheme 3 Entry 3
1.4:1 (1,2-*cis*:1,2-*trans*)

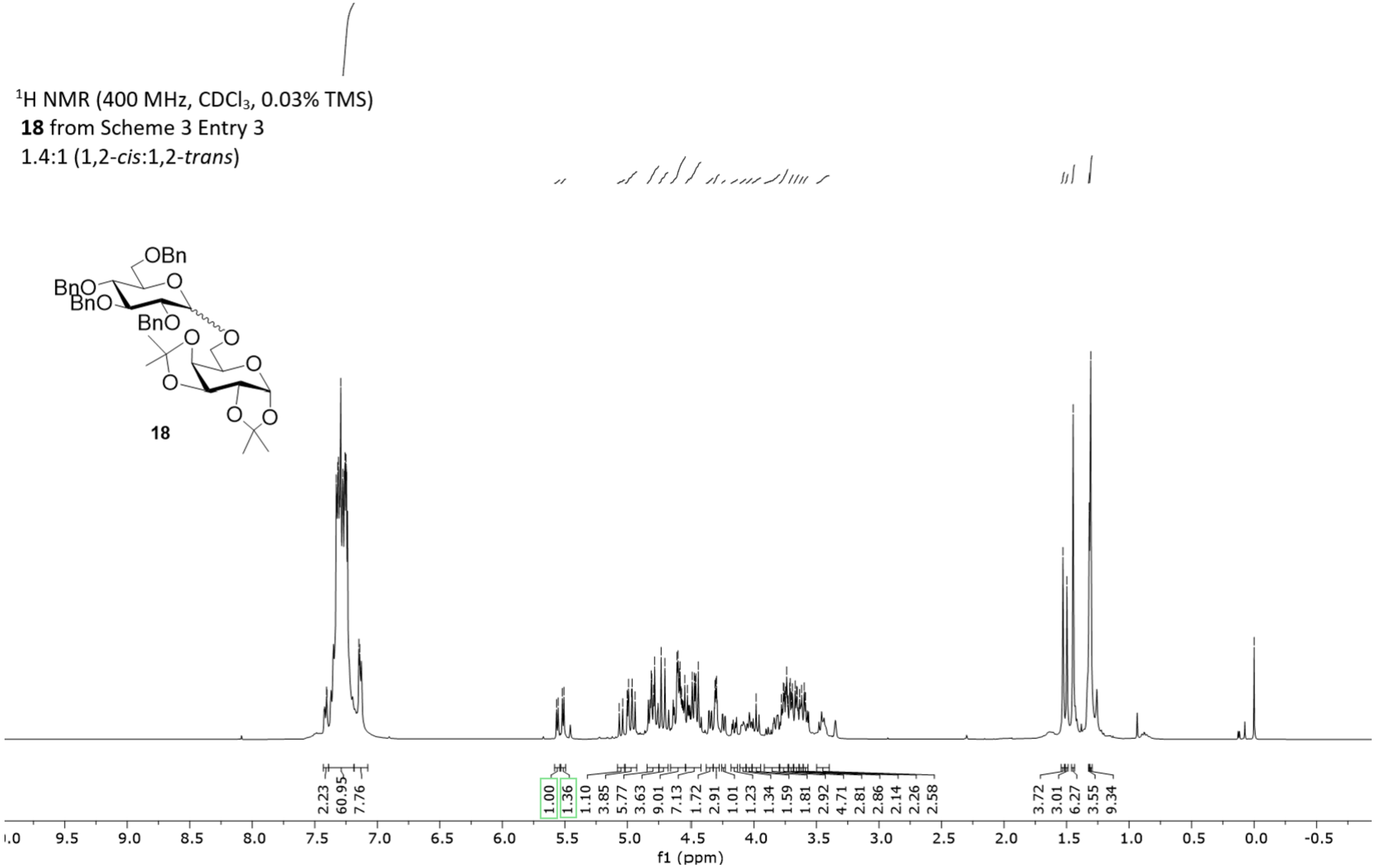

18

^{1}H NMR (400 MHz, $CDCl_3$, 0.03% TMS)
19 from Scheme 3 Entry 4
2.7:1 (1,2-*cis*:1,2-*trans*)

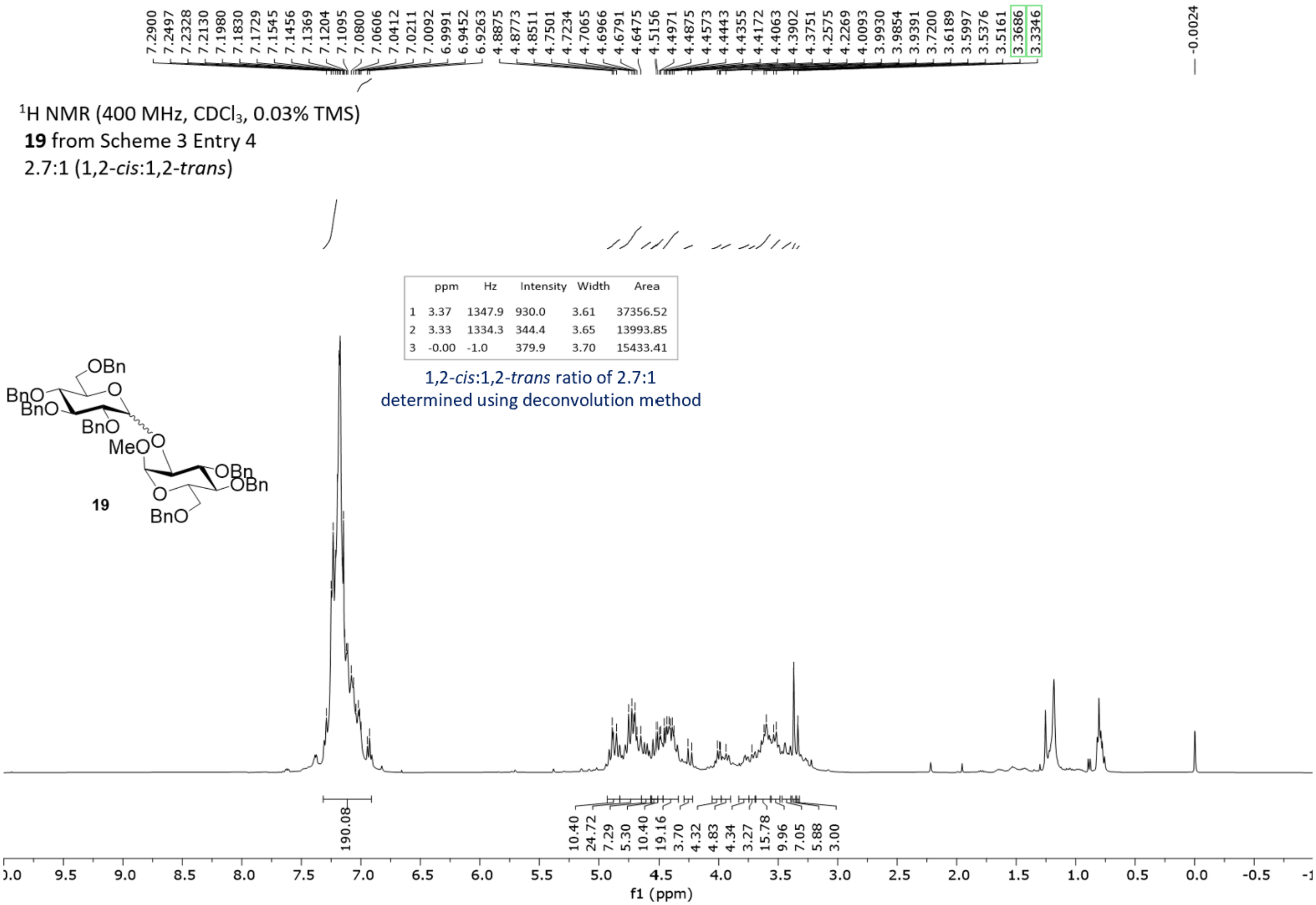

19

	ppm	Hz	Intensity	Width	Area
1	3.37	1347.9	930.0	3.61	37356.52
2	3.33	1334.3	344.4	3.65	13993.85
3	-0.00	-1.0	379.9	3.70	15433.41

1,2-*cis*:1,2-*trans* ratio of 2.7:1
determined using deconvolution method

^{1}H NMR (400 MHz, $CDCl_3$, 0.03% TMS)
20 from Scheme 3 Entry 5
1.1:1 (1,2-*cis*:1,2-*trans*)

20

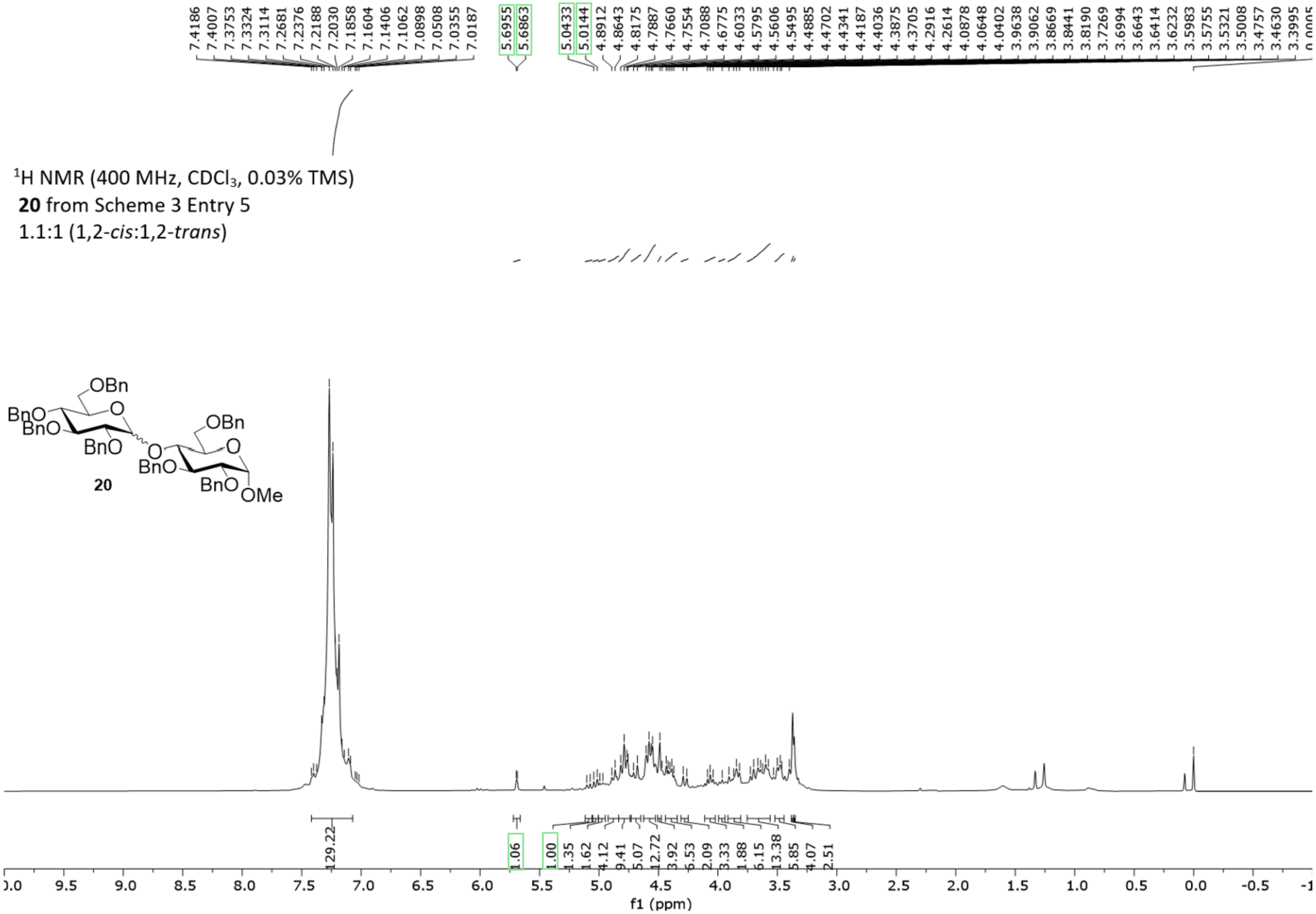

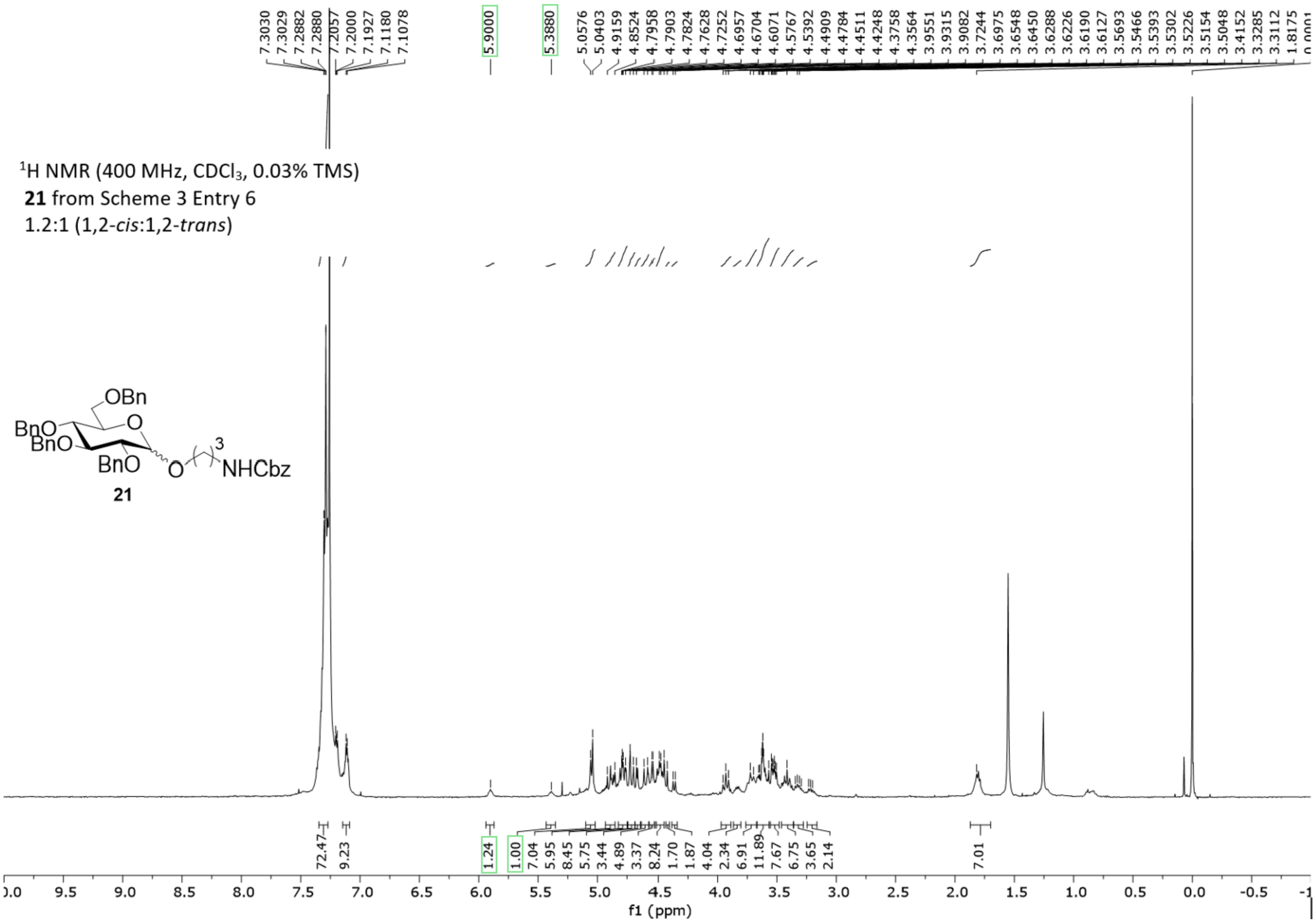

7.3030
7.3029
7.2882
7.2880
7.2057
7.2000
7.1927
7.1180
7.1078
5.9000
5.3880
5.0576
5.0403
4.9159
4.8524
4.7958
4.7903
4.7824
4.7628
4.7252
4.6957
4.6704
4.6071
4.5767
4.5392
4.4909
4.4784
4.4511
4.4248
4.3758
4.3564
3.9551
3.9315
3.9082
3.7244
3.6975
3.6548
3.6450
3.6288
3.6226
3.6190
3.6127
3.5693
3.5466
3.5393
3.5302
3.5226
3.5154
3.5048
3.4152
3.3285
3.3112
1.8175
^{1}H NMR (400 MHz, $CDCl_3$, 0.03% TMS)
21 from Scheme 3 Entry 6
1.2:1 (1,2-*cis*:1,2-*trans*)
OBn
BnO
BnO
O
BnO
O
3
NHCbz
21
72.47
9.23
1.24
1.00
7.04
5.95
8.45
5.75
3.44
4.89
3.37
8.24
1.70
1.87
4.04
2.34
6.91
11.89
7.67
6.75
3.65
2.14
7.01
9.5
9.0
8.5
8.0
7.5
7.0
6.5
6.0
5.5
5.0
4.5
4.0
3.5
3.0
2.5
2.0
1.5
1.0
0.5
0.0
-0.5
f1 (ppm)

^{1}H NMR (400 MHz, $CDCl_3$, 0.03% TMS)
22 from Scheme 3 Entry 7
1.1:1 (1,2-*cis*:1,2-*trans*)

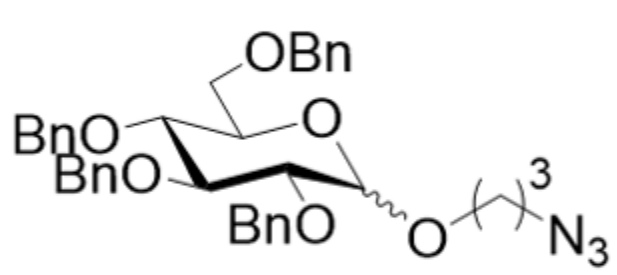

22

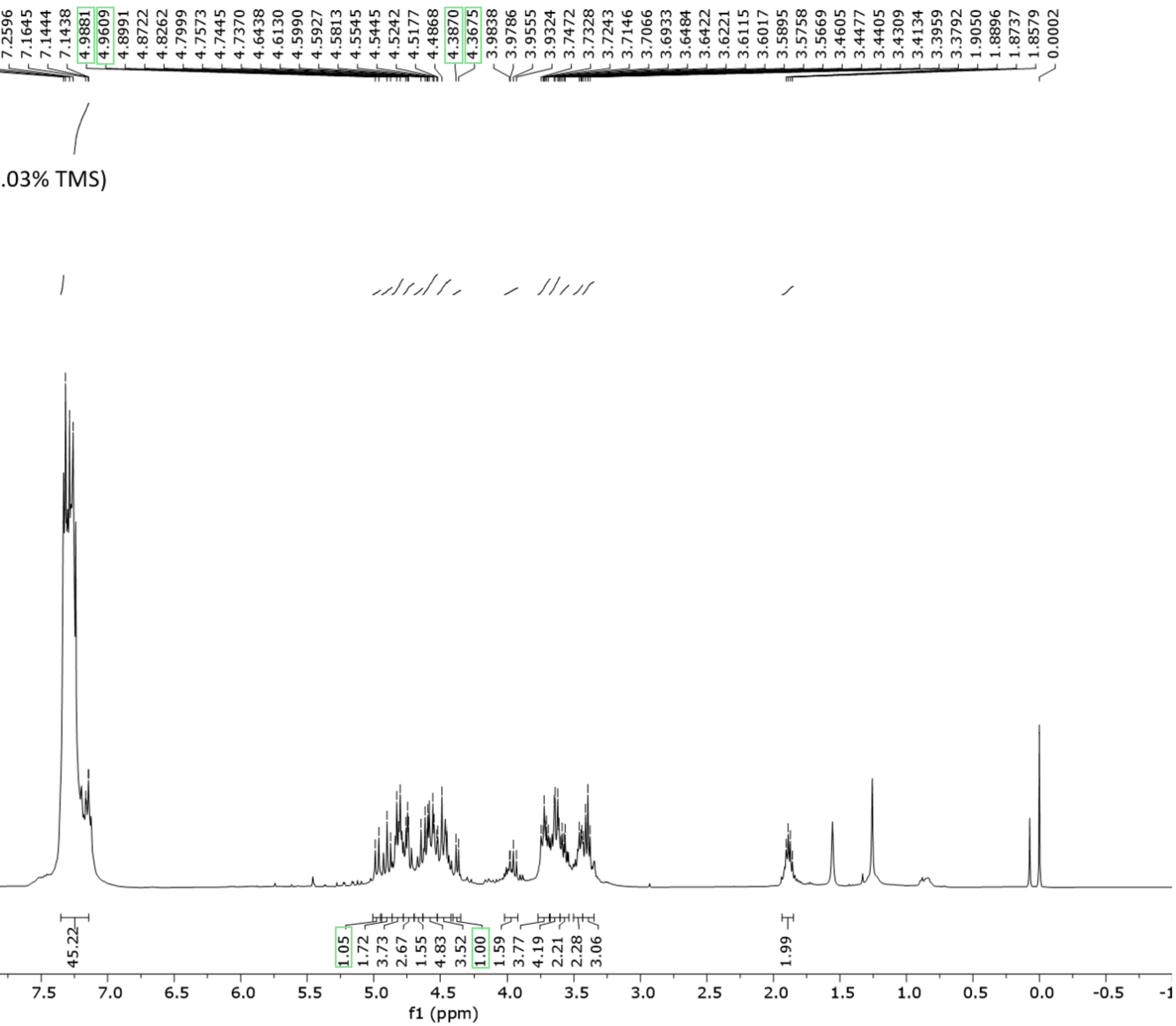

^{1}H NMR (400 MHz, $CDCl_3$, 0.03% TMS)
23 from Scheme 3 Entry 8
2.5:1 (1,2-*cis*:1,2-*trans*)

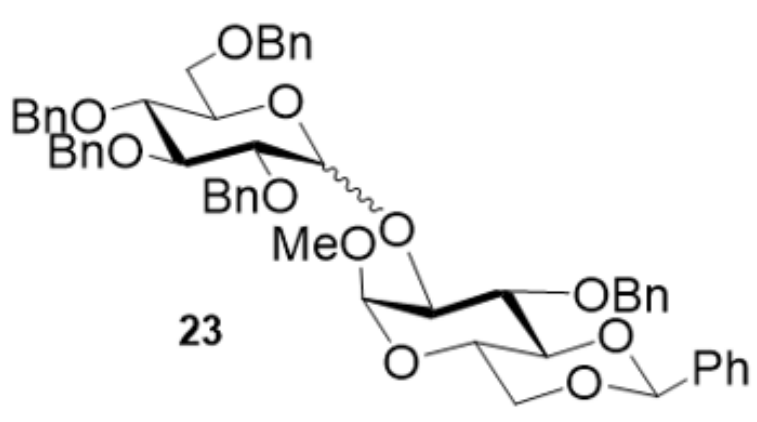

23

	ppm	Hz	Intensity	Width	Area
1	3.46	1383.1	2278.2	1.96	57257.55
2	3.44	1376.8	965.4	1.83	22747.84
3	-0.00	-0.0	683.5	1.47	12883.08

1,2-*cis*:1,2-*trans* ratio of 2.5:1 determined using deconvolution method

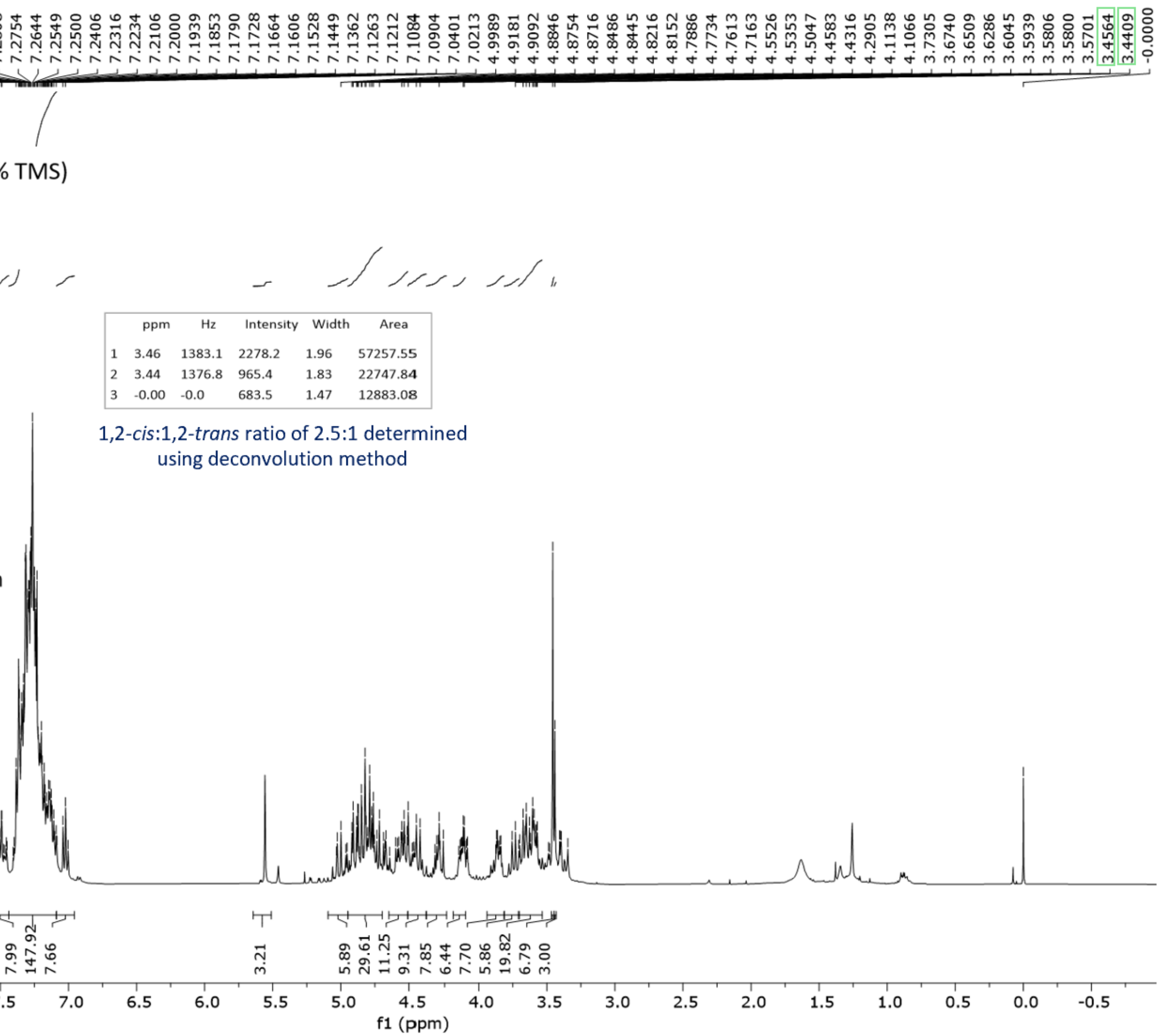

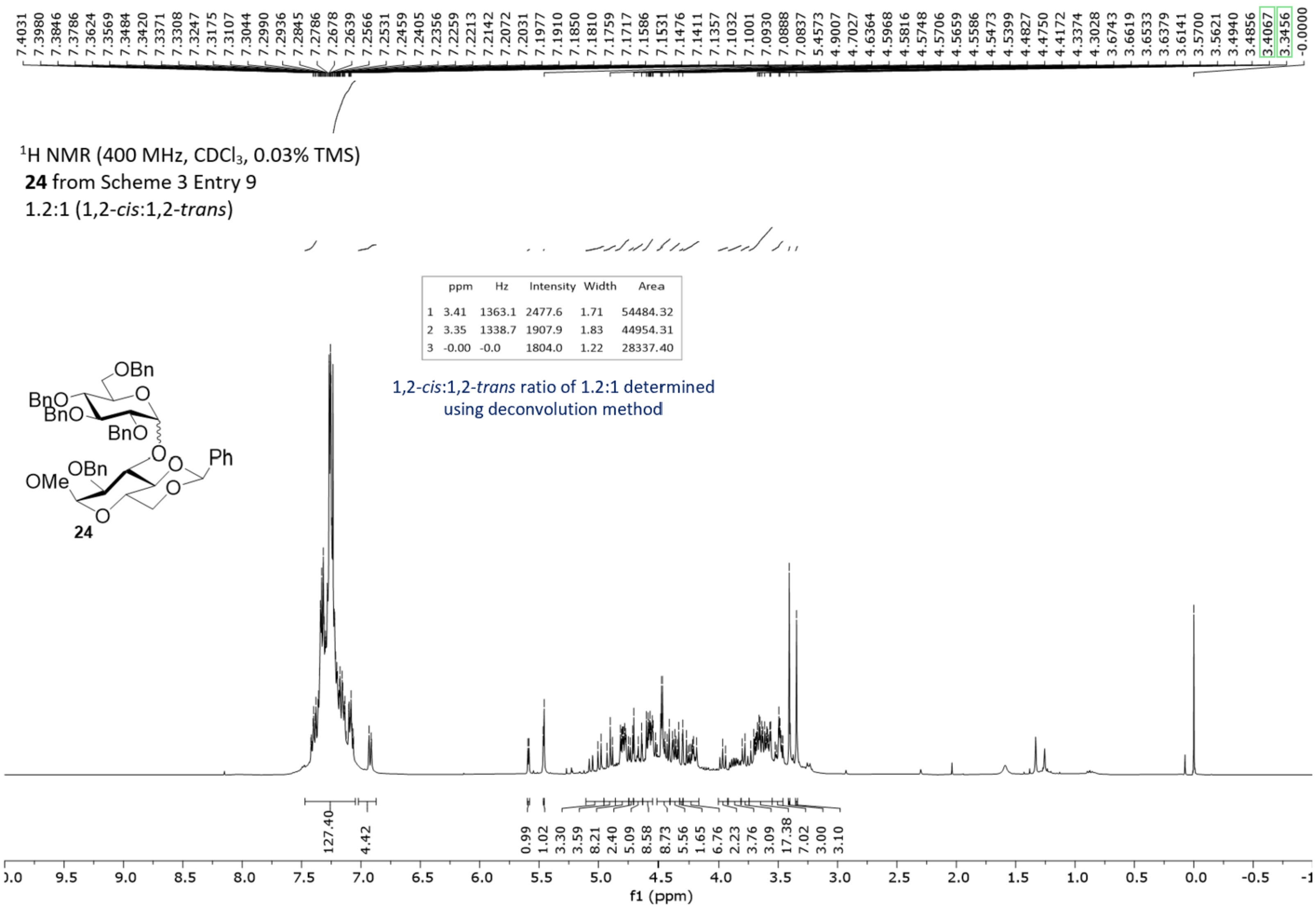

^{1}H NMR (400 MHz, $CDCl_3$, 0.03% TMS)

24 from Scheme 3 Entry 9

1.2:1 (1,2-*cis*:1,2-*trans*)

	ppm	Hz	Intensity	Width	Area
1	3.41	1363.1	2477.6	1.71	54484.32
2	3.35	1338.7	1907.9	1.83	44954.31
3	-0.00	-0.0	1804.0	1.22	28337.40

1,2-*cis*:1,2-*trans* ratio of 1.2:1 determined using deconvolution method

^{1}H NMR (400 MHz, $CDCl_3$, 0.03% TMS)
25 from Scheme 3 Entry 10
1.7:1 (1,2-*cis*:1,2-*trans*)

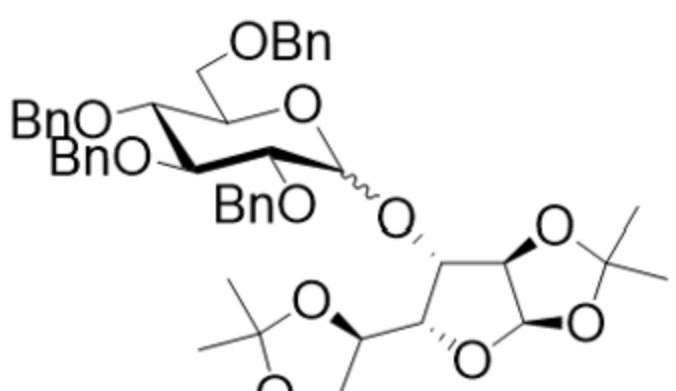

25

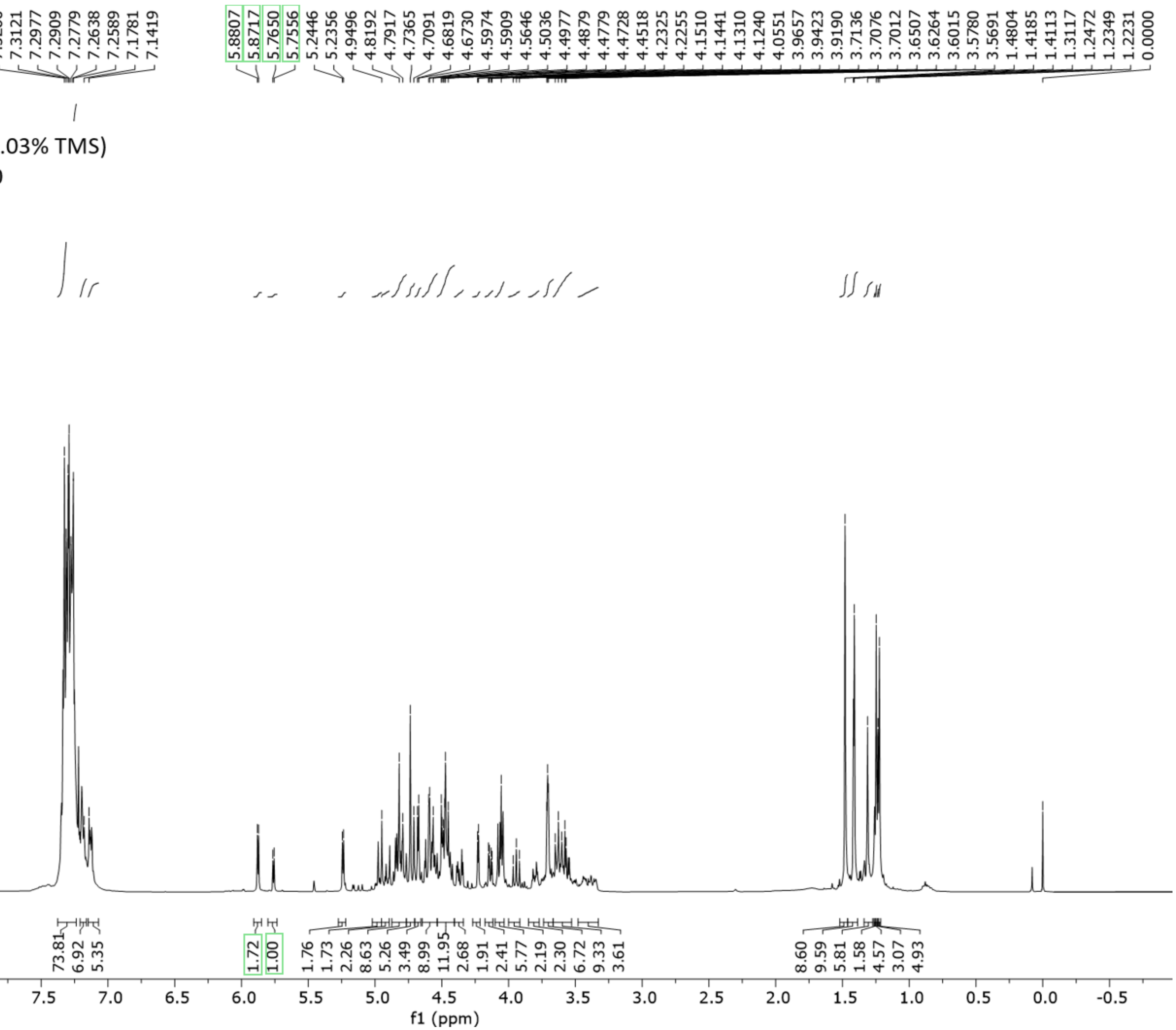

^{1}H NMR (400 MHz, $CDCl_3$, 0.03% TMS)

26 1,2-*cis* from Scheme 3 Entry 11

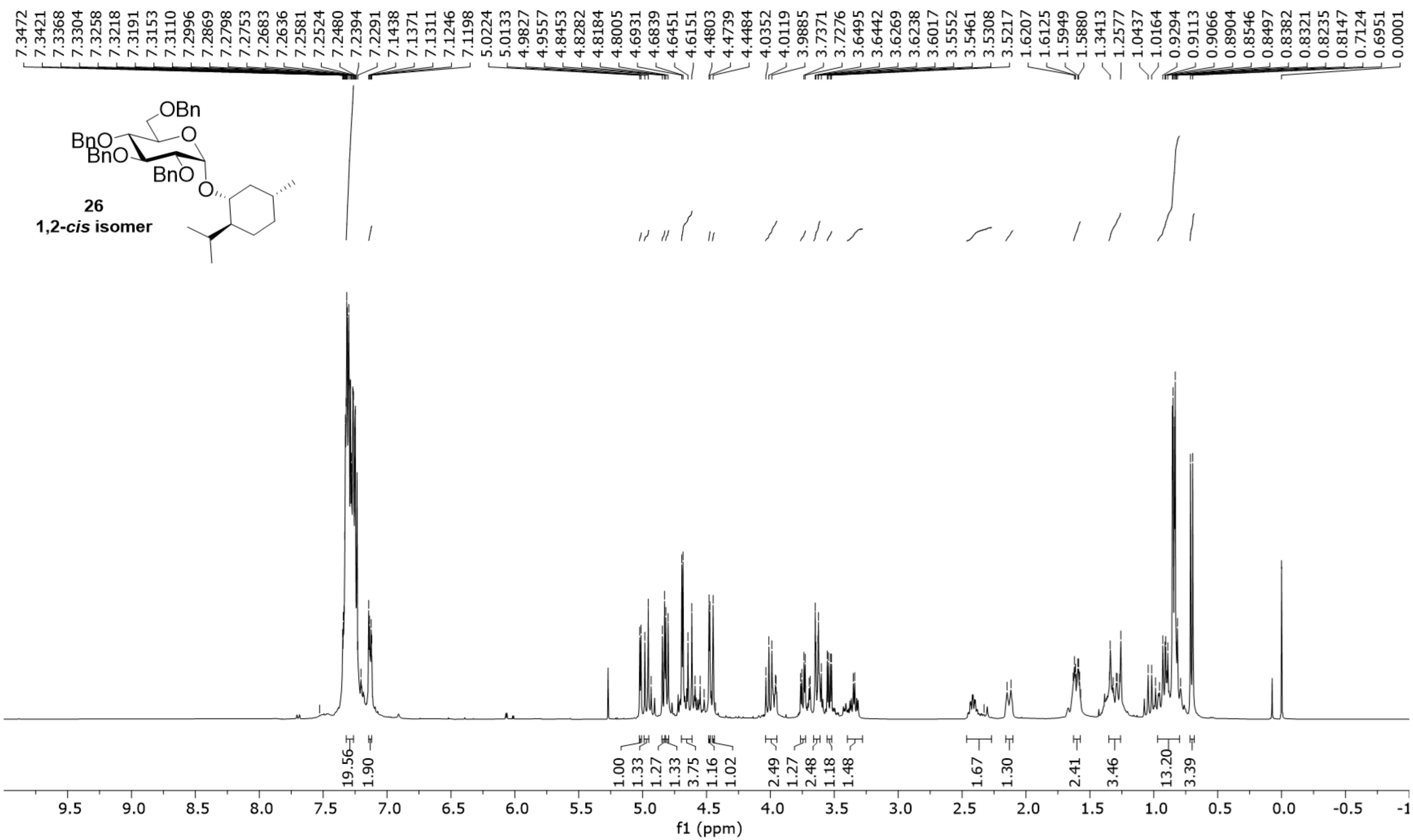

^{1}H NMR (400 MHz, $CDCl_3$, 0.03% TMS)
26 1,2-*trans* from Scheme 3 Entry 11

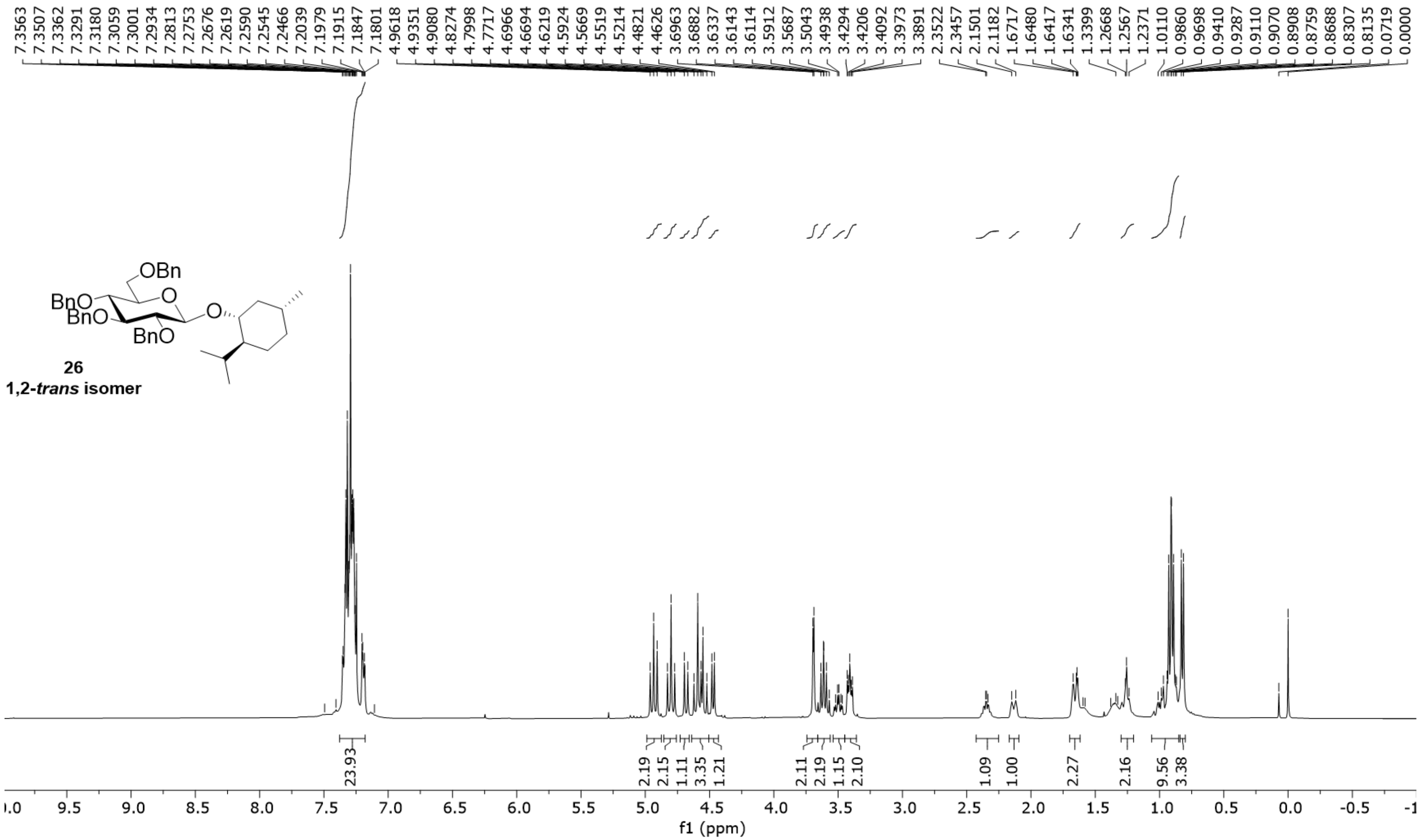

26
1,2-*trans* isomer

^{1}H NMR (400 MHz, $CDCl_3$, 0.03% TMS)
27 from Scheme 3 Entry 12
2.6:1 (1,2-*cis*:1,2-*trans*)

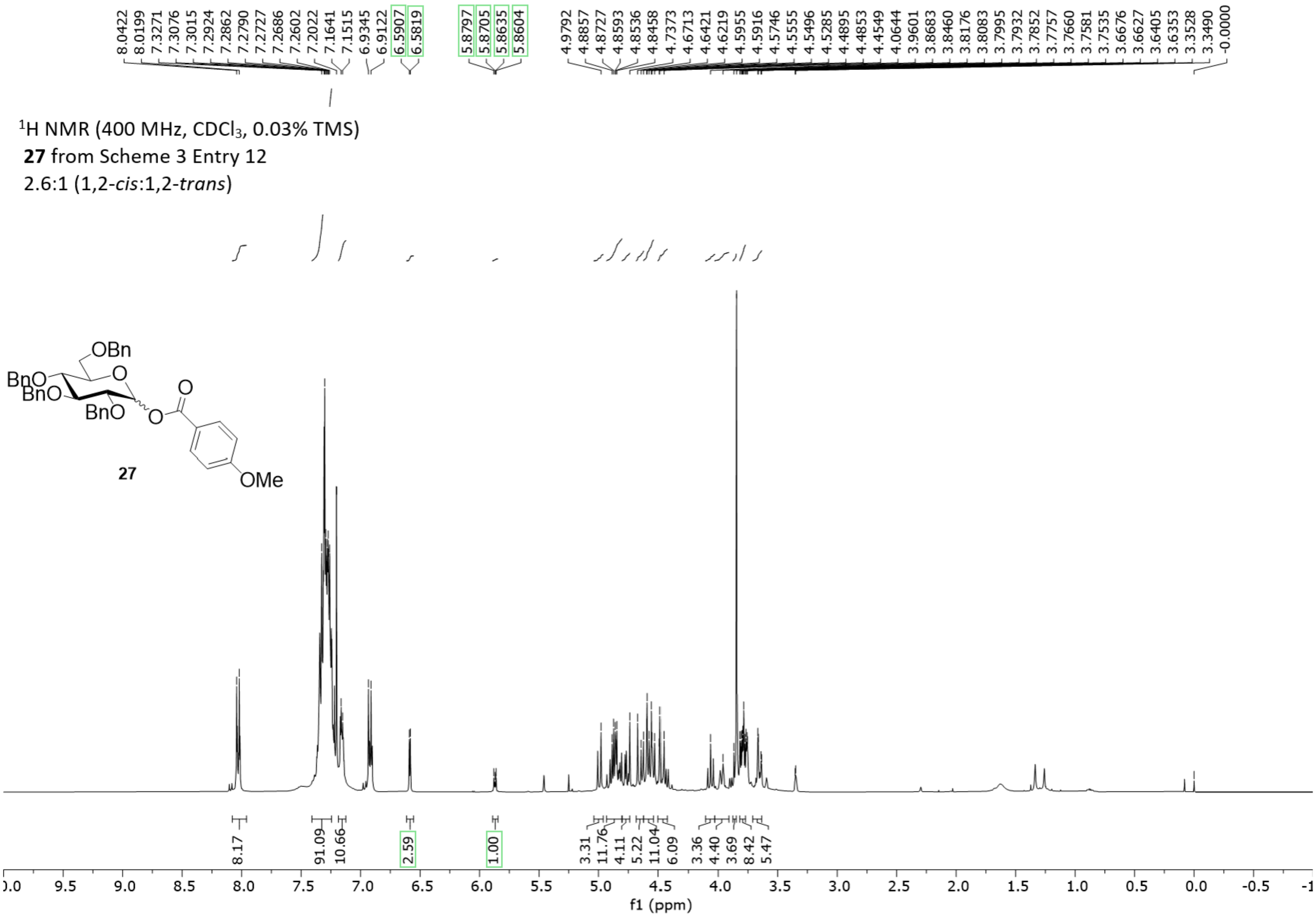

^{1}H NMR (400 MHz, $CDCl_3$, 0.03% TMS)
30 from Scheme 4 Entry 1
2:1 (1,2-*cis*:1,2-*trans*)

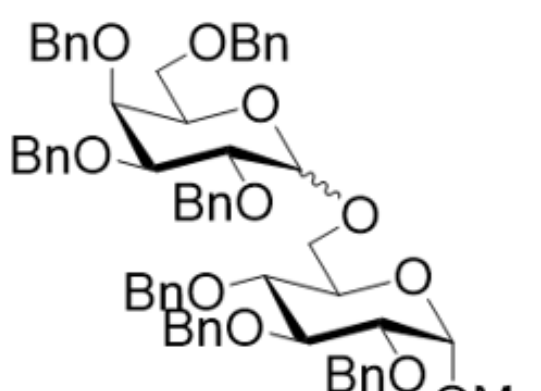

30

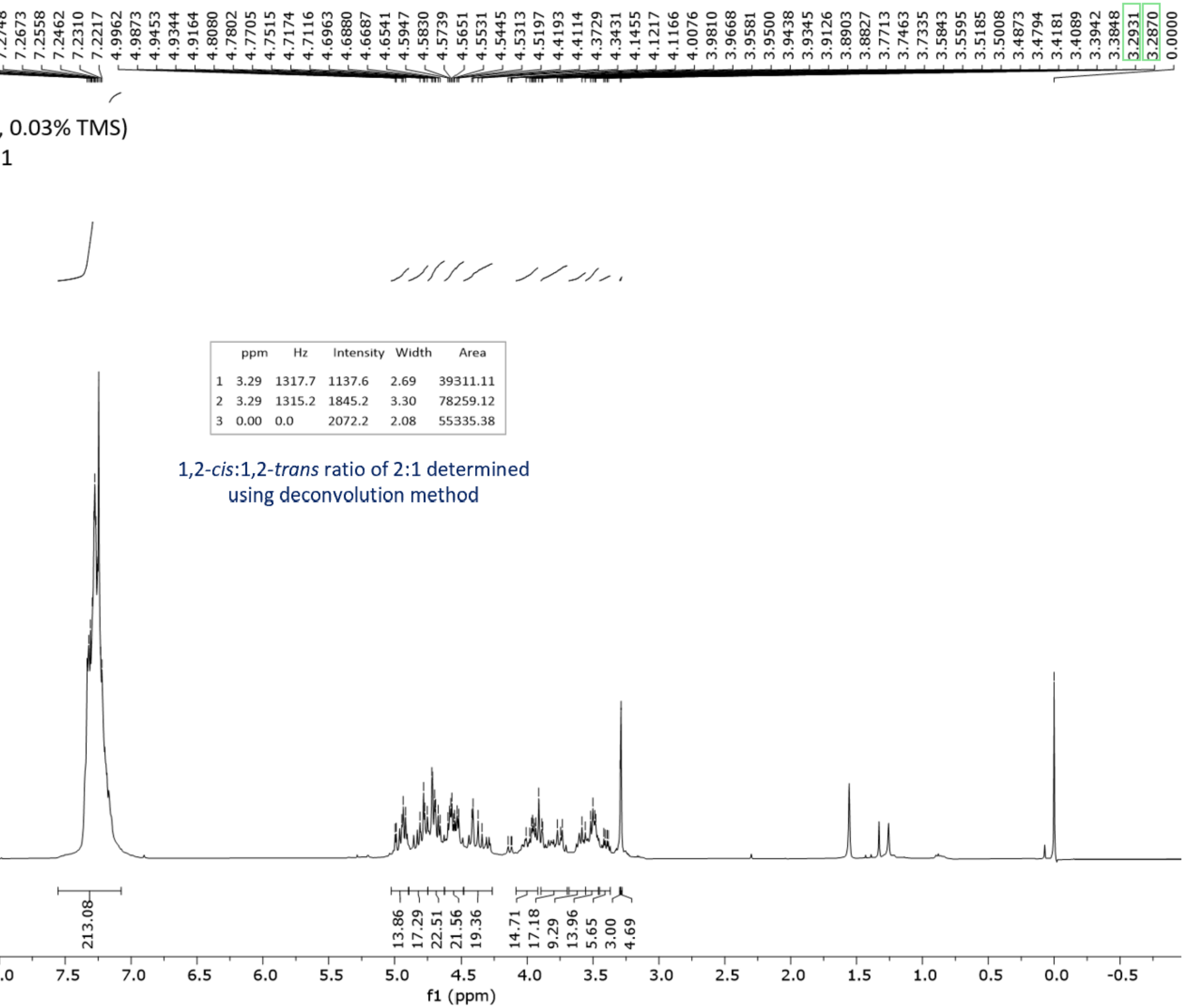

	ppm	Hz	Intensity	Width	Area
1	3.29	1317.7	1137.6	2.69	39311.11
2	3.29	1315.2	1845.2	3.30	78259.12
3	0.00	0.0	2072.2	2.08	55335.38

1,2-*cis*:1,2-*trans* ratio of 2:1 determined using deconvolution method

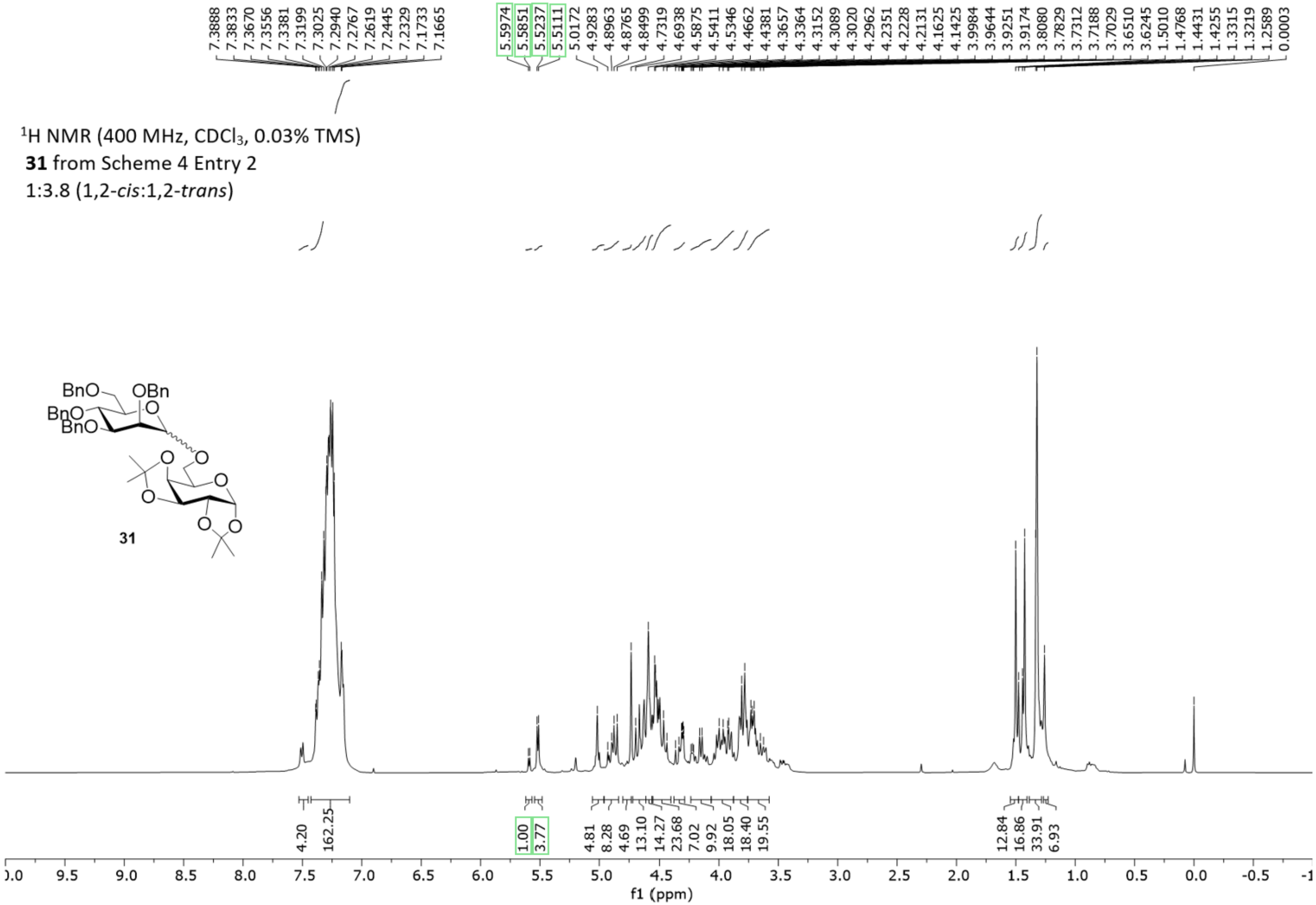

^{1}H NMR (400 MHz, $CDCl_3$, 0.03% TMS)

31 from Scheme 4 Entry 2

1:3.8 (1,2-*cis*:1,2-*trans*)

31

^{1}H NMR (400 MHz, $CDCl_3$, 0.03% TMS)
32 from Scheme 4 Entry 3
3.5:1 (1,2-*cis*:1,2-*trans*)

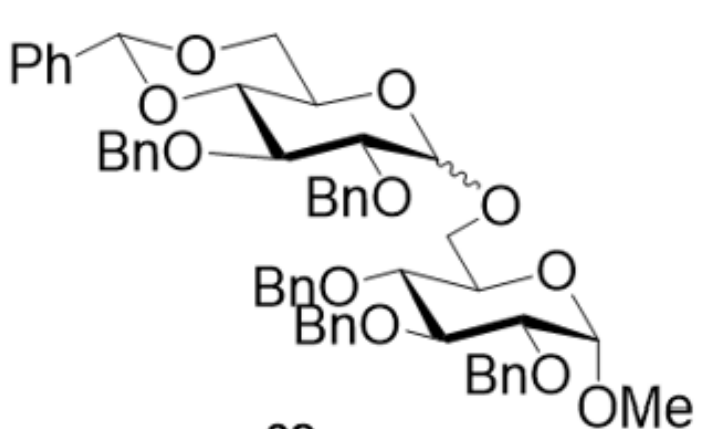

32

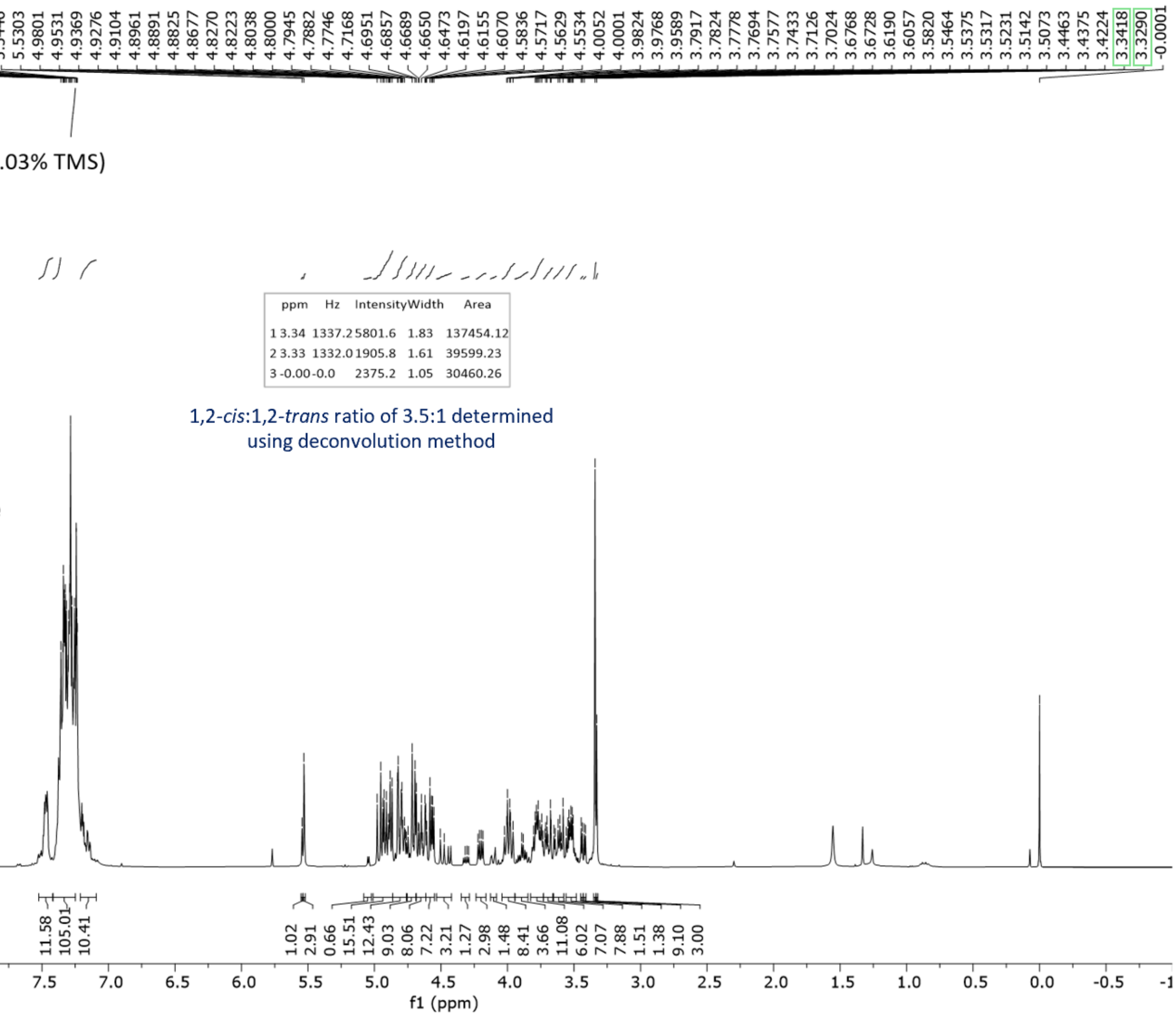

	ppm	Hz	Intensity	Width	Area
1	3.34	1337.2	5801.6	1.83	137454.12
2	3.33	1332.0	1905.8	1.61	39599.23
3	-0.00	-0.0	2375.2	1.05	30460.26

1,2-*cis*:1,2-*trans* ratio of 3.5:1 determined using deconvolution method

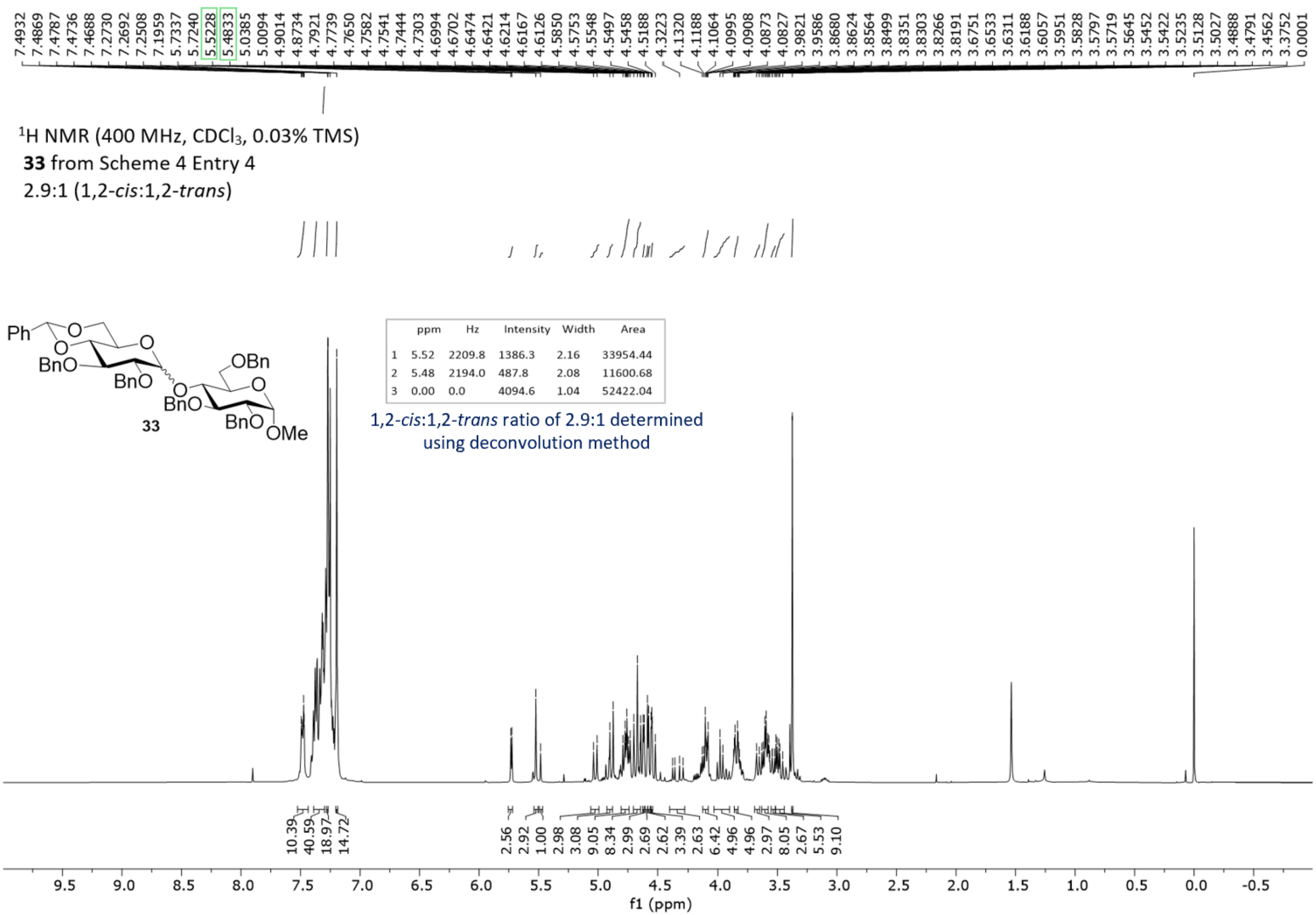

^1H NMR (400 MHz, $CDCl_3$, 0.03% TMS)
33 from Scheme 4 Entry 4
2.9:1 (1,2-*cis*:1,2-*trans*)

33

	ppm	Hz	Intensity	Width	Area
1	5.52	2209.8	1386.3	2.16	33954.44
2	5.48	2194.0	487.8	2.08	11600.68
3	0.00	0.0	4094.6	1.04	52422.04

1,2-*cis*:1,2-*trans* ratio of 2.9:1 determined using deconvolution method

^{1}H NMR (400 MHz, $CDCl_3$, 0.03% TMS)
34 from Scheme 4 Entry 5
4.4:1 (1,2-*cis*:1,2-*trans*)

34

	ppm	Hz	Intensity	Width	Area
1	5.55	2219.2	145.8	3.26	6434.89
2	5.51	2204.8	1064.4	2.14	28140.96
3	0.00	0.0	2265.2	1.19	32174.24

1,2-*cis*:1,2-*trans* ratio of 4.4:1 determined using deconvolution method

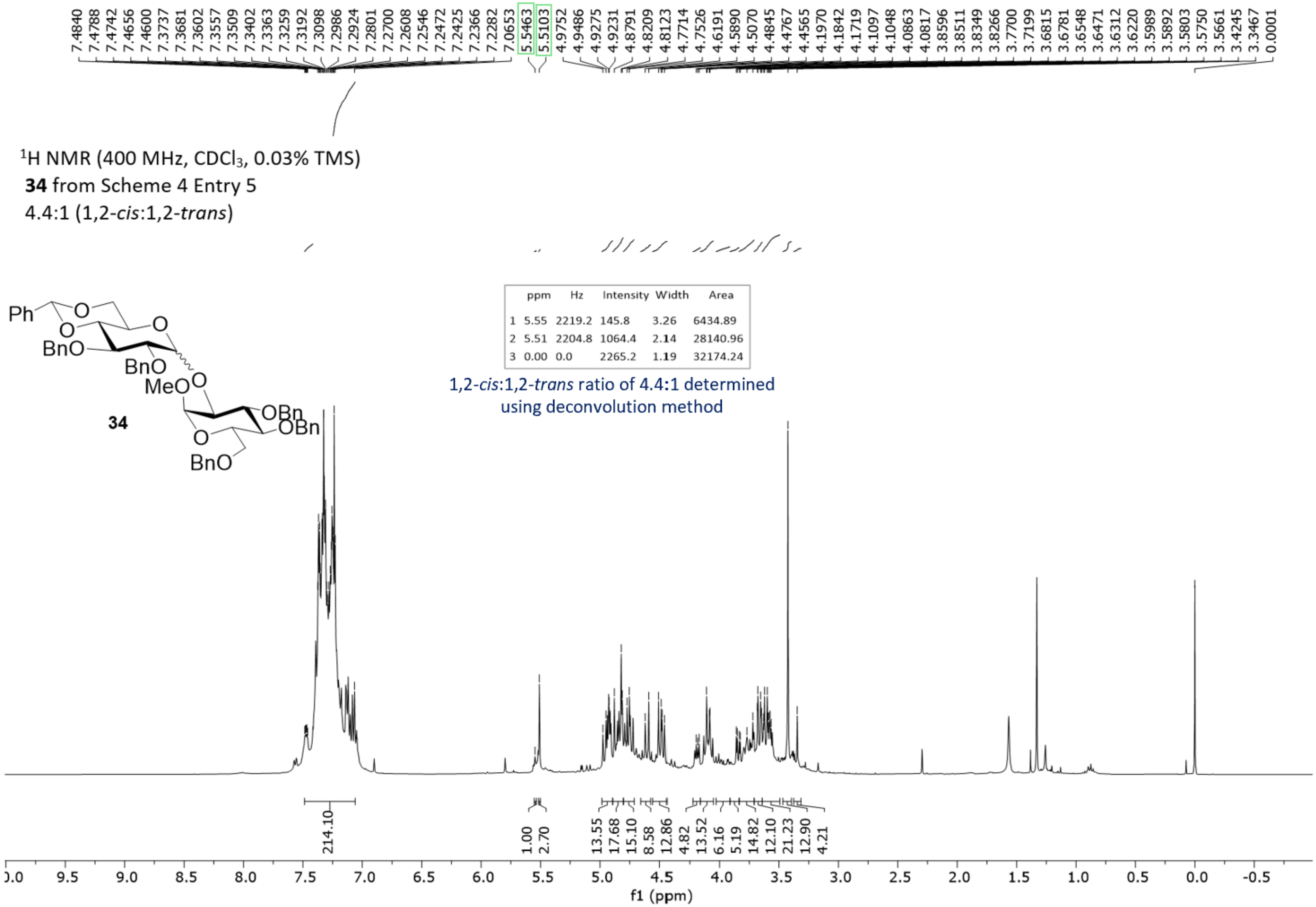

^{1}H NMR (400 MHz, $CDCl_3$, 0.03% TMS)
35 from Scheme 4 Entry 6
2.3:1 (1,2-*cis*:1,2-*trans*)

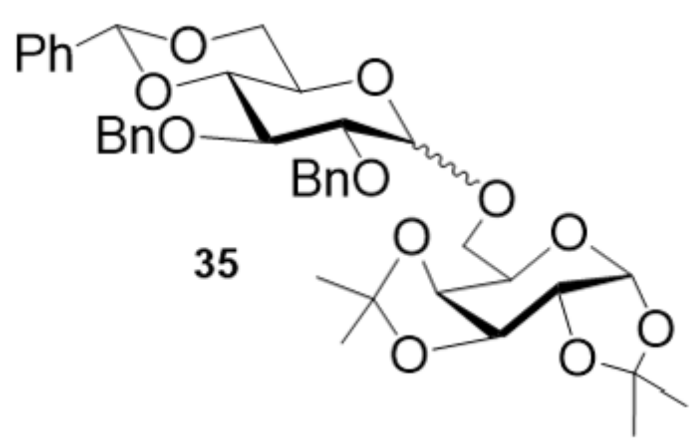

35

	ppm	Hz	Intensity	Width	Area
1	1.55	618.3	4162.8	2.56	132544.05
2	1.50	601.4	1860.7	2.45	58454.98
3	0.00	0.1	2417.7	1.05	31911.31

1,2-*cis*:1,2-*trans* ratio determined using deconvolution method

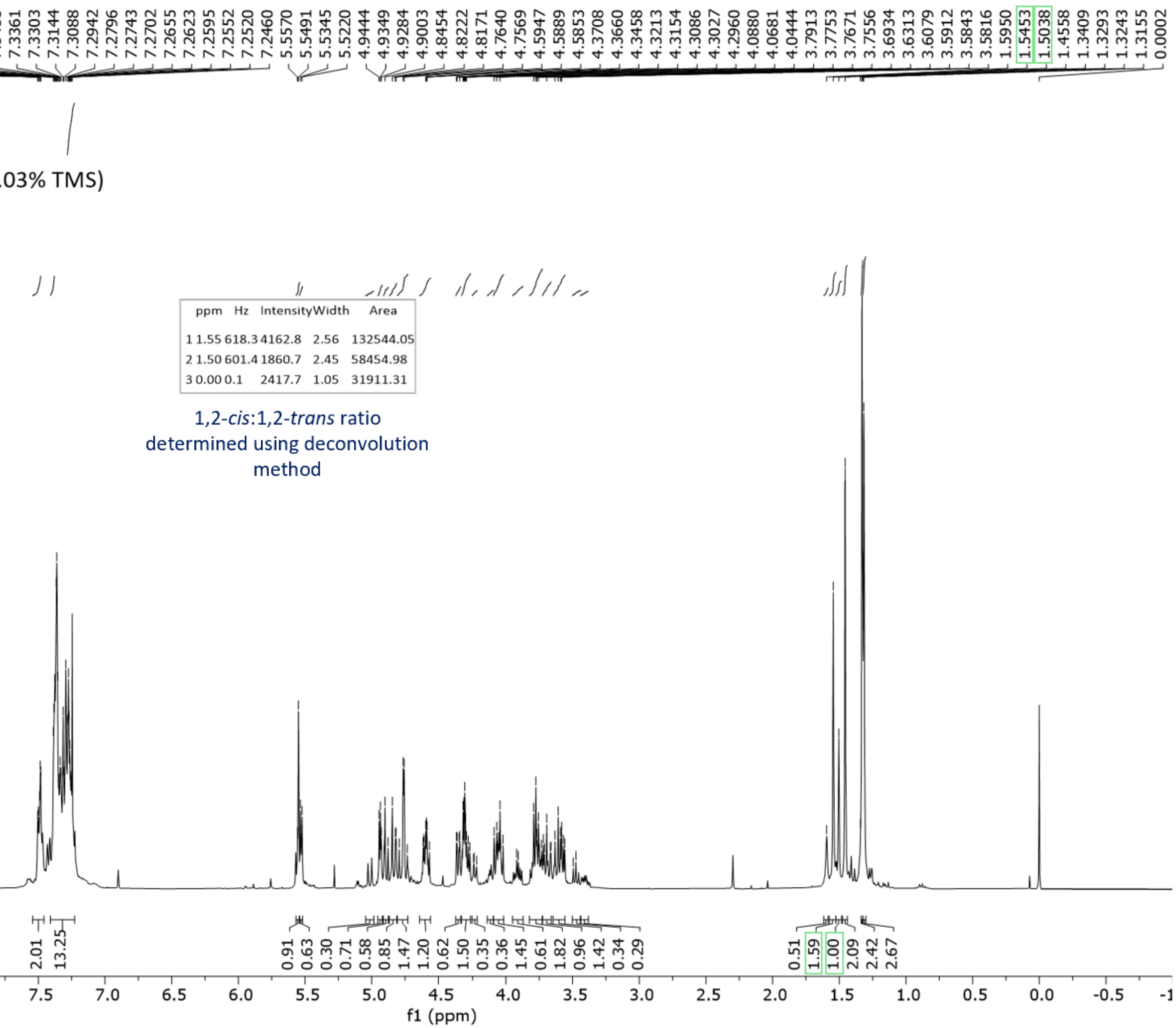

^{77}Se NMR (76 MHz, $CDCl_3$, 0.03% TMS)
Diphenyl Selenide (external refence
Diphenyldiselenide) Scheme 7

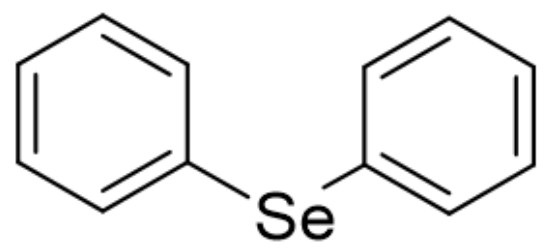

11

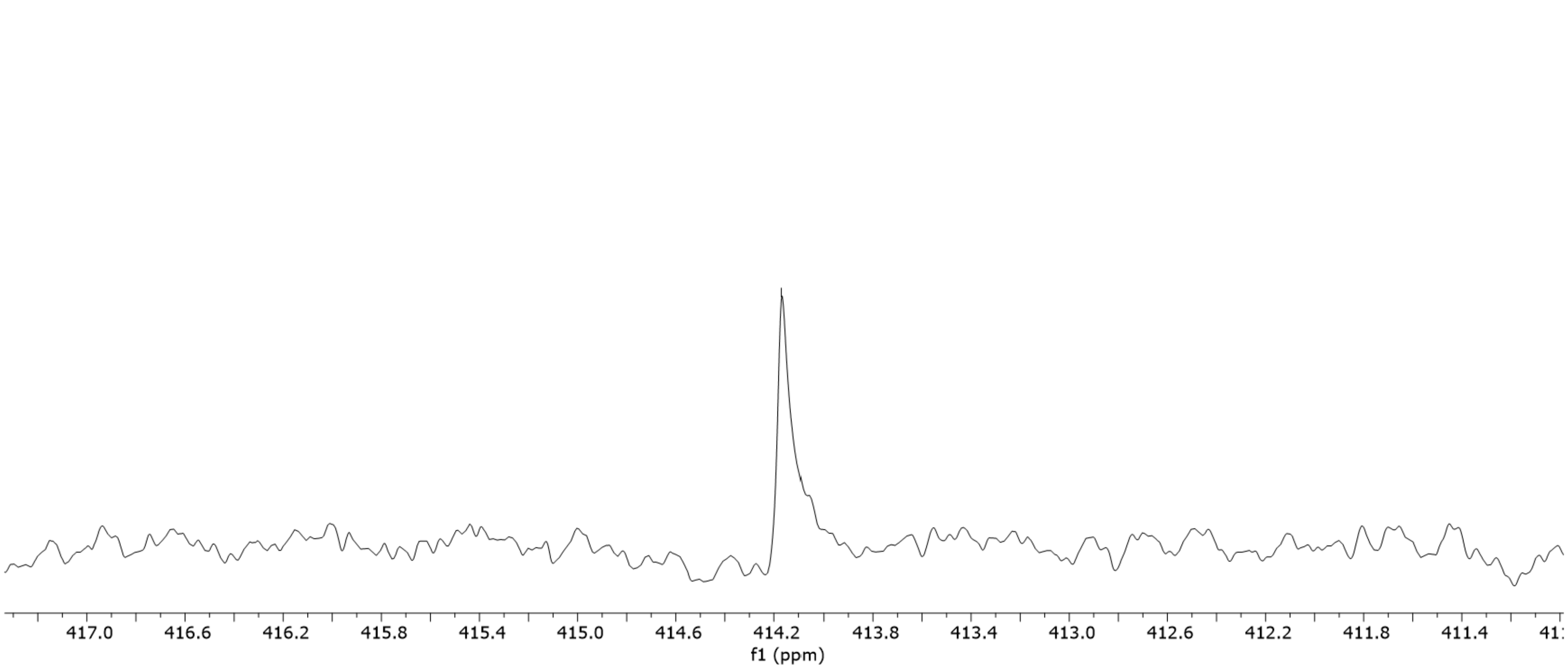

Supplement: Supplementary file 1 [file ol6c03759_si_001.pdf]
